# Acylated Ghrelin as a Multi-Targeted Therapy for Alzheimer's and Parkinson's Disease

**DOI:** 10.3389/fnins.2020.614828

**Published:** 2020-12-14

**Authors:** Niklas Reich, Christian Hölscher

**Affiliations:** ^1^Biomedical & Life Sciences Division, Lancaster University, Lancaster, United Kingdom; ^2^Neurology Department, A Second Hospital, Shanxi Medical University, Taiyuan, China; ^3^Research and Experimental Center, Henan University of Chinese Medicine, Zhengzhou, China

**Keywords:** growth hormone secretagogue receptor 1 alpha, mitochondrial dysfunction, inflammation, autophagy, insulin resistance, dopamine, neurodegeneration, ghrelin

## Abstract

Much thought has been given to the impact of Amyloid Beta, Tau and Alpha-Synuclein in the development of Alzheimer's disease (AD) and Parkinson's disease (PD), yet the clinical failures of the recent decades indicate that there are further pathological mechanisms at work. Indeed, besides amyloids, AD and PD are characterized by the culminative interplay of oxidative stress, mitochondrial dysfunction and hyperfission, defective autophagy and mitophagy, systemic inflammation, BBB and vascular damage, demyelination, cerebral insulin resistance, the loss of dopamine production in PD, impaired neurogenesis and, of course, widespread axonal, synaptic and neuronal degeneration that leads to cognitive and motor impediments. Interestingly, the acylated form of the hormone ghrelin has shown the potential to ameliorate the latter pathologic changes, although some studies indicate a few complications that need to be considered in the long-term administration of the hormone. As such, this review will illustrate the wide-ranging neuroprotective properties of acylated ghrelin and critically evaluate the hormone's therapeutic benefits for the treatment of AD and PD.

## Introduction

Alzheimer's disease (AD) and Parkinson's disease (PD) are multi-faceted neurodegenerative diseases that reach far beyond the accumulation and aggregation of Amyloid Beta (Aß), Tau and Alpha (α)-synuclein. Indeed, the last decades of research have indicated that cognitive decline is driven by the interplay of various pathologic processes, involving insulin-associated bioenergetic impairments and the reduced cerebral metabolization of glucose (Neth and Craft, 2017), mitochondrial defects (Bose and Beal, 2016; Onyango et al., 2016), vascular abnormalities, reduced blood flow, blood brain barrier (BBB) damage (Kisler et al., 2017; Sweeney et al., 2018), dysfunctional autophagy and mitophagy (Kerr et al., 2017; Fujikake et al., 2018; Liu J. et al., 2019), oxidative stress (Cenini et al., 2019), chronic systemic inflammation, pathological immune cell infiltration into the brain (Amor and Woodroofe, 2014; Anderson et al., 2014; Stephenson et al., 2018), inflammasome activation (Ising et al., 2019; Stancu et al., 2019), demyelination (Wang S. S. et al., 2018), the degeneration of axons (Kandan et al., 2013), the development of type 2 diabetes mellitus (T2DM), the cerebral desensitization of growth and neurotrophic factors, in particular insulin (Gault and Holscher, 2018; Holscher, 2019), as well as alterations of the dopaminergic system, including the extensive atrophy of substantia nigra pars compacta (SNpc)-located dopaminergic neurons and dopamine depletion in the striatum in PD (Martorana and Koch, 2014; Poewe et al., 2017).

Especially in the case of AD, countless Aβ and a handful of Tau-directed therapies have been tested in clinical trials, yet none of them, including a very recent phase II trial with the anti-Tau antibody semorinemab, have come to fruition. Such discouraging findings have come to question the amyloid hypothesis, as reflected by the notably diminished numbers of Aβ-based and elevated quantities of neuroprotection-focussed and anti-inflammatory approaches in the clinic in 2019. Moreover, while Aβ may be the undisputed culprit in familial AD patients with respective genetic mutations (<1%), sporadic AD patients (>99%) may endure several other risk factors, such as secondary inflammatory conditions, head injuries, the APOE_4_ allele, T2DM/insulin resistance and brain glucose hypometabolism, the presence of metabolic and vascular syndrome and presumably many more. This is paralleled in the varying clinical profile, as sporadic AD patients may exhibit high or low Aβ_1−42_ burden, with or without the prevalence of Tau of Lewy body biomarkers, in the cerebrospinal fluid. This suggests that multiple pathologic, but also protective, factors cooperate in the progression of AD and that that a differential treatment regimen, which commonly necessitates the use of multiple drugs for chronically advancing disorders, might be necessary for individual patients. Therefore, monotherapies are presumably an ineffective way of approaching AD and PD and are more likely to fail, supporting the concept that multi-targeted therapies are more profitable (Iqbal and Grundke-Iqbal, 2010; Adams, 2020; Huang et al., 2020).

## The Ghrelin System and Its Physiological Role

Belonging to a group of physiologically secreted hormones, ghrelin serves numerous important functions. Ghrelin is predominantly produced by gastric X/A-like cells that are located in the oxyntic gland of the stomach (Date et al., 2000), although a lower degree of the hormone is also expressed in various peripheral tissues, in lymphocytes and in the CNS (Ferrini et al., 2009). In a serious of catalytic steps, the precursor preproghrelin is expressed, cleaved to proghrelin and transported to the Golgi body, where it may be acylated by the linkage of an O-linked octanoyl lipid group (C:8.0) at Ser^3^ via ghrelin O-acyltransferase (GOAT). Ultimately, following translocation to the endoplasmic reticulum (ER), proghrelin is further processed by prohormone convertase 1/3 to generate the 28 amino acid-long anorexigenic hormone ghrelin. Mature ghrelin is stored within secretory granules of X/A-like cells and released into the bloodstream upon fasting to stimulate appetite (Cummings et al., 2001; Yanagi et al., 2018). Conversely, increased circulatory levels of glucose and long-chain fatty acids (LCFA) following meal intake as well as the postprandial release of insulin block the secretion of ghrelin (Gagnon and Anini, 2012; Lu et al., 2012; Sakata et al., 2012). Depending on the presence or absence of the acyl group at Ser^3^, mature ghrelin can be further distinguished into its active form, acylated ghrelin (AG), and desacylated ghrelin (DAG) (Hosoda et al., 2000; Yanagi et al., 2018). The acylation state of ghrelin is transient, however, as liberated AG is continually deacetylated by acyl-protein thioesterase 1 and butyrylcholinesterase in the blood stream (De Vriese et al., 2004; Satou et al., 2010; Schopfer et al., 2015). Through the circulatory system, AG is able to reach and cross the blood brain barrier (BBB) in either direction through the recognition of the lipophilic acyl/octanyloid side chain and saturable systems, whereas DAG obtains brain entry through non-saturable diffusion through the BBB (Banks et al., 2002; Diano et al., 2006). Furthermore, although still unidentified, the liberation of fasting-associated plasma factors appear to further stimulate the BBB translocation of AG (Banks et al., 2008). Notably, the presence of GOAT has been detected in human serum, the hippocampus and the temporal gyrus (Gahete et al., 2010; Goebel-Stengel et al., 2013; Murtuza and Isokawa, 2018). It has been verified that DAG can be locally modified by GOAT, which presumably allows ghrelin to exert centralized effects in selected tissues and brain areas, such as the hippocampus (Murtuza and Isokawa, 2018). Ultimately, AG stimulates intracellular downstream signaling through its cognate G-protein coupled receptor (GPCR), known as the growth hormone secretagogue receptor type 1α (GHS-R1α). Importantly, DAG is incapable of interacting with GHS-R1α, yet the existence of distinct DAG-binding receptors has been postulated (Howard et al., 1996; Yanagi et al., 2018).

GHS-R1α is widely transcribed in multiple key areas of the brain, such as the hippocampus, hypothalamus, cortex, ventral tegmental area (VTA), SN, spinal cord, dorsal and median raphe nuclei, sympathetic preganglionic nerves and endothelial cells of the cerebral vasculature, yet it is also expressed by various immune cells and in peripheral tissue (Guan et al., 1997; Hosoda et al., 2000; Gnanapavan et al., 2002; Jiang et al., 2006; Pan et al., 2006; Ferens et al., 2010). Notably, only GHS-R1α, but not its truncated and non-functional splicing variant GHS-R1ß, is capable of interacting with AG. In contrast, a dominant-negative role for GHS-R1ß has been suggested, in which heterodimerization of GHS-R1ß with GHS-R1α encourages receptor endocytosis to obstruct intracellular signaling (Leung et al., 2007).

As a major metabolic hormone, AG elevates the secretion of growth hormone (GH) by the pituitary gland, reduces insulin, yet increases glucagon secretion by pancreatic cells and promotes the hepatic release of glucose into the blood, thus maintaining steady plasma glucose levels during fasting (Mani et al., 2019). Furthermore, AG induces the expression of the orexigenic peptides neuropeptide Y (NPY) and agouti-related protein (AgRP) in the hypothalamus to stimulate appetite, as extensively described in Yanagi et al. (2018). Other physiological processes that are commanded by ghrelin include the regulation of the gastrointestinal motility and acid secretion, cardiac function, osteoblast proliferation, bone maturation and muscular/myoblast outgrowth, the formation of long-term memory, the control of behaviors such as spontaneity, anxiety, food/reward behavior as well as the navigation of the circadian rhythm (Abdalla, 2015; Shi et al., 2017; Yanagi et al., 2018).

Following receptor stimulation in the brain, AG exerts a broad range of neuroprotective effects and has, thus, emerged as a potential candidate for the treatment of AD and PD. Despite the selectivity of GHS-R1α for AG, DAG has demonstrated its own neuromodulatory effects, although the underlying signaling mechanisms remain a mystery and appear to be limited to the periphery (Yanagi et al., 2018). For the sake of this review and in association to AD and PD, the focus will be placed on the multifarious neuroprotective actions of AG.

## Mitochondria and the Neuronal Energy Metabolism

### Impairments in the Mitochondrial Function and Adenosine Triphosphate Production Are Key Events in Alzheimer's and Parkinson's Disease'

Generally, it has been well-established that mitochondrial dysfunction, originating from genetic mutations of key mitochondrial proteins, environmental toxins, excessive oxidative stress, or aging, is a key driver of PD. Similarly, oxidative stress and pathological Aß, which accumulates in mitochondria, depolarizes the mitochondrial membrane potential, inhibits electron transport chain (ETC) enzymes and provokes the production of reactive oxygen species (ROS), trigger mitochondrial and, thus, bioenergetic defects in AD. The mitochondrial pathology in AD and PD is further exacerbated by impaired mitochondrial biogenesis, a mechanism that leads to the generation of additional mitochondria to meet greater energetic demands in cells, and mitophagy, which is a form of autophagy that mediates the degradation of malfunctional, ROS-overproducing mitochondria (covered in chapter 4) (Bose and Beal, 2016; Onyango et al., 2016; Fang et al., 2019; Liu J. et al., 2019).

Importantly, it must be pointed out that all cellular functions necessitate energy and, therefore, the availability of two bioenergetic substrates: ATP and guanosine-5'-triphosphate, which may be converted into ATP. The latter is generated in mitochondria through the ETC. In this oxygen-requiring process, electrons (H^+^) are drawn from the reducing agents NADH and FADH_2_ and funneled through the inner mitochondrial membrane to the outer compartment of the mitochondrion via complex I, III, and IV. This establishes an electrochemical proton gradient (also known as protonmotive force Δ*p*) and, thus, elicits the influx of electrons from the outer to the inner compartment through complex 5 (ATP synthase), which subsequently converts ADP to ATP. To recharge NAD^+^ and FAD^+^ and to resume ATP generation, the brain relies on glucose as major energy substrate as well as its metabolization in the tricarboxylic acid cycle (TCA) as main bioenergetic pathway (Penicaud et al., 2002; Arun et al., 2016; Martinez-Reyes and Chandel, 2020). Generally, functional deficits in complex 1 are associated with PD and defects in complex IV are implicated in AD (Cottrell et al., 2002; Arun et al., 2016). Given the pivotal role of mitochondrial dysfunction in PD, toxins that are selectively taken up by dopaminergic neurons and impair mitochondrial complex 1, such as 1-methyl-4-phenyl-1,2,3,6-tetrahydropyridine (MPTP), kill SN-located, dopaminergic neurons and induce Parkinsonism in humans and rodents (see Figure 2) (Langston et al., 1983; Meredith and Rademacher, 2011).

As reviewed in Muddapu et al. (2020), the neuronal populations in the hippocampal CA1 region as well as the SN are more vulnerable toward metabolic deregulation, which may be linked to the progression of AD (CA1) and PD (SN). Generally, the oxidative phosphorylation of glucose via the TCA poses the primary energy source for neurons and the cellular stress associated with aging and neurodegenerative diseases, for example amyloid aggregation or the prevalence of genetic risk factors, provoke greater bioenergetic demands. As a direct consequence of these higher energetic needs, the neuronal mitochondria are forced to generate excessive amounts of ATP at the cost of the elevated co-production of ROS. The increased oxidative burden, on the other hand, may subsequently spark glial dysfunction and the excessive release of glutamate, NMDA/AMPA receptor activation and aberrant intraneuronal Ca^2+^-amassment, neuroinflammation and astroglial scar formation, inflammation-driven permeabilization of the BBB and pro-inflammatory cytokine-driven insulin resistance. It is incompletely understood whether mitochondrial defects elicit insulin resistance or vice versa, however. It has also been hypothesized that both factors might negatively influence each other (Neth and Craft, 2017). In any way, the desensitization of insulin in the CNS is linked to reduced cerebral glucose uptake, the diminished liberation of lactate, another pivotal energy source for neurons, by astrocytes and chronic glucose hypometabolism (Muddapu et al., 2020), as evident in the brains of both AD (Lyingtunell et al., 1981; Hoyer et al., 1988; Ogawa et al., 1996; Drzezga et al., 2003; Mosconi et al., 2008) and PD patients (Huang et al., 2008; Hosokai et al., 2009; Liepelt et al., 2009; Borghammer et al., 2010; Berti et al., 2012). It has been postulated that the development of cerebral insulin resistance, in the long-term, enforces the utility of energy sources other than glucose and the TCA cycle. Most notably, this bioenergetic shift is thought to preferentially promote the β-oxidation of ketone bodies (lipids) to produce ATP in the brain (see Neth and Craft, 2017).

More implicit, mitochondrial dysfunction is connected to the impairment of key effectors. As indicated in an AD animal model, APPswe/PS1dE9 mice displayed reduced hippocampal levels of the catalytic α2-subunit of 5' adenosine monophosphate-activated protein kinase (AMPK) (Pedros et al., 2014), which is a master effector that upregulates ATP synthesis, curbs ATP utility, maintains the mitochondrial homeostasis and navigates mitophagy when the cellular energy stores are depleted (Herzig and Shaw, 2018). Additionally, the transcription of the biogenesis-mediators PGC1α and mitochondrial nuclear respiratory factor (NRF)1/2 were reduced in the hippocampi of the APPswe/PS1dE9 mice (Pedros et al., 2014). Strikingly diminished levels of the mitochondrial markers PGC1α, succinate dehydrogenase complex A (which participates in the TCA cycle) and translocase of outer mitochondrial membrane 20 have also been observed in the post-mortem-derived SNpc of PD patients. In the context of PD, as further confirmed by genetic deletion in rodents, PGC1α is crucial for the survival of SNpc-located dopaminergic neurons and, thus, dopamine production (Jiang et al., 2016). As such, the function of various mitochondrial master modulators, including AMPK and PGC1α, is disturbed in AD and PD. For more information about AG's influence on insulin resistance and glucose hypometabolism in the CNS (chapter 7) as well as the dopaminergic pathology (chapter 8), please see the respective chapters.

### Acylated Ghrelin Ameliorates Oxidative Stress and Enhances the Mitochondrial Function, Adenosine Triphosphate Generation and Biogenesis

**Figure 1:**
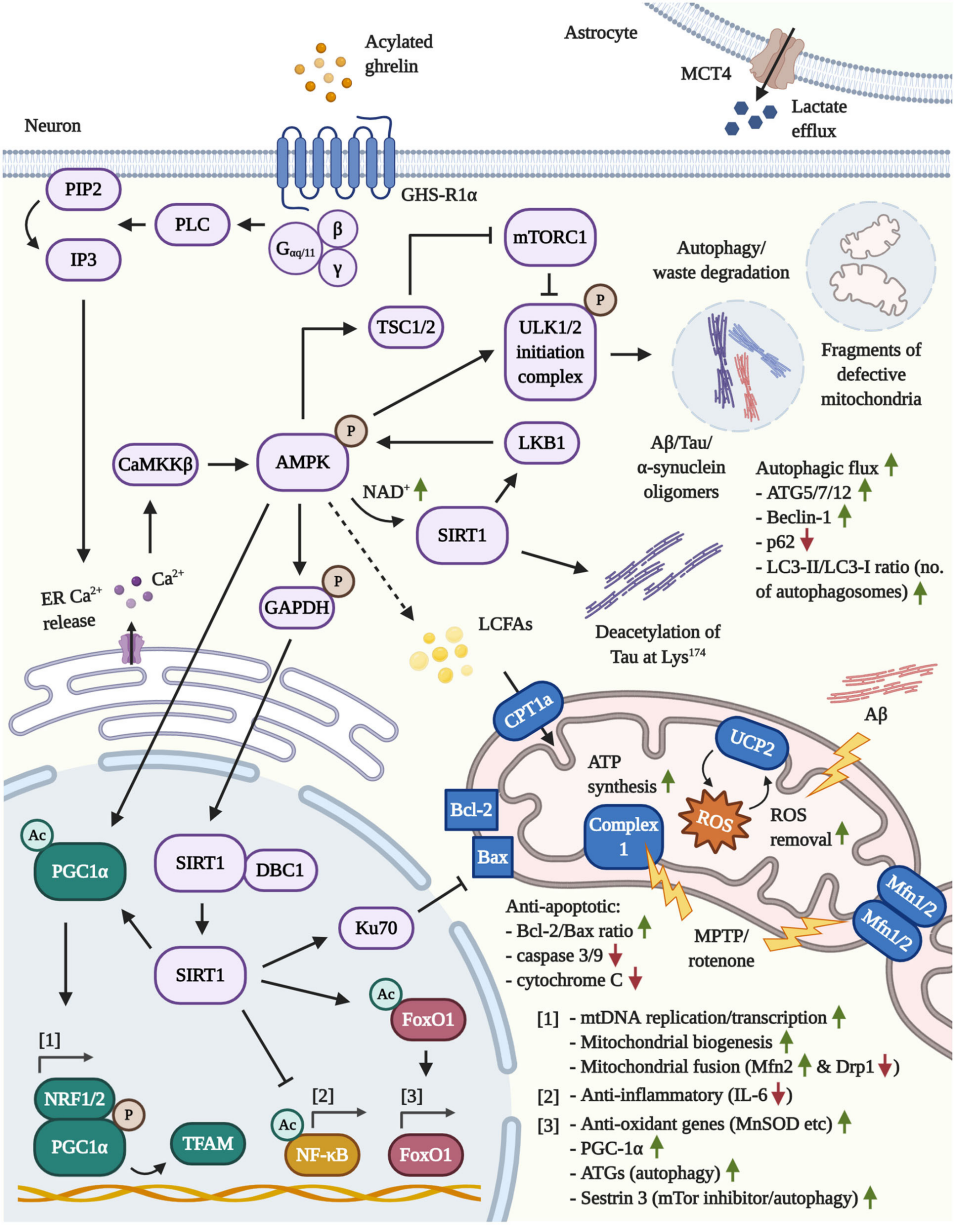
Illustration of the neuroprotective pathways following GHS-R1α activation by AG or ghrelin agonists in neurons and astrocytes. [1] Mitochondrial function: By activating the key mediator AMPK, AG induces the transcriptional co-activator PGC1α. The latter, in concert with NRF1/2, enhances mitochondrial biogenesis, the synthesis of TFAM and TFAM-mediated mtDNA replication/transcription. By increasing the transcription of Mfn2, PGC1α protects from MPTP/rotenone-driven mitochondrial fragmentation. In addition, AMPK/GAPDH-mediated phosphorylation of nuclear SIRT1 frees the latter deacetylase and leads to the inactivation of pro-inflammatory NF-κB, the activation of the Bax-sequestrating Ku70 and the stimulation of FoxO1-regulated anti-oxidant and autophagy genes. Lastly, the induction of the AMPK/CPT1a/UCP2 pathway prevents pathological mitochondrial depolarization (such as by Aβ). Furthermore, UCP2-driven mitochondrial uncoupling increases the mitochondrial respiration, bioenergetic efficiency and mitigates the co-generation of ROS by the ETC, which may protect from the stress-induced hyperproduction of ATP and ROS during early stages of AD. Given that more advanced stages of AD are characterized by neuronal glucose hypometabolism and a chronic shift toward other bioenergetic processes, in particular the β-oxidation of lipids, AG may support the compensatory execution of β-oxidation to generate ATP. Notably, AMPK inhibits ACC, thus depleting the intracellular malonyl-CoA pools and, in turn, elevating the activity levels of the malonyl-CoA-regulated CPT1a (not shown). [2] Autophagy: (Macro)autophagy is primarily driven by the GHS-R1α/AMPK/TSC_1/2_-mediated inactivation of mTOR/mTORC1 and the direct phosphorylation of ULK1 via AMPK, resulting in the degradation of cellular waste, amyloids (Aβ/Tau/α-synuclein) and defective mitochondria. Moreover, by raising the intracellular NAD^+^ levels, AMPK reinforces its activity through the activation of the cytoplasmic, NAD^+^-dependent SIRT1 and the AMPK-kinase LKB1. SIRT1 is also involved in the deacetylation of Tau at Lys^174^, which was reported to abrogate the pathological propagation of Tau throughout the brain. Besides triggering autophagy, AG upregulates various ATGs and Beclin-1, while promoting autophagosome maturation and the autophagic flux. [3] Astrocytes: The stimulation of GHS-R1α encourages the expression of the lactate-efflux transporter MCT4 by astrocytes, leading to the increased secretion of lactate, a potent energy source for neurons.

In the context of mitochondrial dysfunction, AG strengthens the mitochondrial vigor in multiple ways. Notably, as depicted in Figure 1, AG drives mitoprotection and autophagy by activating shared key effectors. While mitochondria-based investigations are limited in the field of AD, it was demonstrated that AG guards primary rat and N42 hypothalamic neurons from Aß oligomer-provoked depolarization of the mitochondrial membrane (Martins et al., 2013; Gomes et al., 2014). Further mechanistic insight can be derived from studies in PD models. In the 1-methyl-4-phenyl-1,2,5,6 tetrahydropyridine (MPTP) mouse model of PD, AG protected from neuronal death in the SNpc, as displayed by the normalized B-cell lymphoma 2 (Bcl-2)/Bax ratio and lowered caspase-3 activity, stimulated the neuronal activity, elevated the production of multiple LCFAs, for instance palmitic acyl CoA (C16:0), and improved the mitochondrial respiration by activating the ROS-buffering, mitochondrial uncoupling protein 2 (UCP2).

Furthermore, AG promoted the mitochondrial biogenesis, resulting in increased numbers of nuclear respiratory factor (NRF)1-positive mitochondria (Jiang et al., 2008; Andrews et al., 2009a; Donadelli et al., 2014). Based on previous propositions, AG may support the execution of the mitochondrial ß-oxidation and evoke the generation of LCFAs as a fuel source for ATP production upon mild negative energy balance in the CNS (Andrews et al., 2009a; Horvath et al., 2009). This idea must be addresses with care, however, since AG differentially navigates lipid metabolism in a tissue-specific manner. In the periphery, for example, independent of the hormone's orexigenic effects, AG stimulated the expression of lipogenic enzymes (fatty acid synthase (FAS), lipoprotein lipase and more) and lessened the transcription of carnitine palmitoyltransferase Ia (CPT1a), a rate-limiting effector necessary to induce fatty acid oxidation, in white adipose tissue to promote fat storage (Theander-Carrillo et al., 2006; Perez-Tilve et al., 2011). In stark contrast, AG enhanced fatty acid oxidation and lipolysis in mouse skeletal muscles (Kraft et al., 2019). In the brain, it was discovered that AG selectively diminishes the expression of the lipogenesis-affiliated FAS in the VMH, which appears to be a site-specific process to promote ß-oxidation and induce the expression of anorexigenic NPY. In the other hand, AG does not modulate FAS in other brain areas, including the amygdala, striatum, hippocampus, several cortical regions and others (Lopez et al., 2008; Yanagi et al., 2018). Moreover, under physiological conditions, AG was shown to discourage, rather than elevate, fatty acid oxidation in the hypothalamic ARC and in the cortex (Lage et al., 2010; Gao et al., 2013; Mir et al., 2018). As such, at least under physiological conditions, AG presumably does not induce β-oxidation in brain areas other than the VMH. On the other hand, it must be noted that the prevalence of cerebral insulin resistance during AD leads to defects in the metabolism of glucose, which is believed to provoke the use of β-oxidation and lipids (ketones) as primary energy sources for neurons (Neth and Craft, 2017). Thus, it is plausible that AG may assist the compensatory execution of ß-oxidation in neurons that may occur during more advanced stages of AD and, possibly, PD. As a word of caution, while the β-oxidation of lipids has been proven in astrocytes, its utility by neurons is yet to be verified (Tracey et al., 2018). In any case, given that neurons enter an initial hyperglycolytic state and overproduce ATP plus, inevitably, ROS in their mitochondria to cope with the additional cellular stress in AD and, presumably also, PD (Neth and Craft, 2017; Muddapu et al., 2020), AG may alleviate glucose hypermetabolism and the associated oxidative stress. By activating the ROS-ablating UCP2 and driving mitochondrial biogenesis, AG enhances the functionality and bioenergetic efficiency of mitochondria during AD and PD, as further outlined below.

Of note, mutational studies in UCP2-modified and MPTP-treated mice revealed that UCP2 is a joint key mediator in the protection of SN-VTA dopaminergic neurons from apoptosis, the decrease of ROS as well as the increase of mitochondrial biogenesis (Andrews et al., 2005; Conti et al., 2005). Cell culture studies have implied that AG increases the steady state levels of UCP2 by preventing its ubiquitination and degradation, resulting in the cellular accumulation of this ROS-quenching protein (Zhang, 2017). Mechanistically, AG induces UCP2 by inhibiting acetyl-CoA carboxylase (ACC), leading to the intracellular depletion of malonyl-CoA stores and, thus, the activation of the LCFA-transporter CPT1a in the outer mitochondrial membrane (Yanagi et al., 2018). In conjunction with Acyl-CoA synthases in the outer and acylcarnitine translocase plus CPT2 in the inner mitochondrial membrane, respectively, CPT1a delivers and processes converts LCFAs into acyl-CoA and delivers the latter into the inner mitochondrial compartment for β-oxidation and ATP production (Schlaepfer and Joshi, 2020). As a direct consequence of β-oxidation, nascent fatty acids are generated in the inner-mitochondrial compartment and used as “flip-flopping” proton translocators by UCP2 to shuttle H^+^ into the inner-mitochondrial compartment. This process results in mitochondrial uncoupling, partially dispels and reduces the electrochemical proton gradient (Δ*p*) that is maintained by the ETC and forestalls the Δ*p*-dependent generation of ROS at complex I and III. As such, the induction of UCP2 buffers the glucose/TCA-exacerbated production of ROS by the ETC (see Jezek et al., 2018 for an extensive description of UCP functions), which may be protective in AD and PD. Similar to the SNpc (Andrews et al., 2009a), AG also augmented the induction of UCP2, mitochondrial respiration and mitochondrial abundance in hypothalamic NPY/AgRP neurons *in vivo* (Andrews et al., 2009b). Moreover, AG rescued neurons from apoptosis and caspase-3 activation in a UCP2-dependent manner, improved the mitochondrial ATP generation plus total ATP levels and alleviated the ROS load in the brains of rodents that were subjected to cardiac arrest and/or traumatic brain injury (Lopez et al., 2012a; Xu et al., 2019). In concert with the animal experiments, AG decreased the ROS burden, rescued the toxin-induced dysfunction of complex 1 in mitochondria, normalized the mitochondrial transmembrane potential and prohibited apoptosis, as shown with improvements in the Bcl-2/Bax ratio, cytochrome C release and caspase-3/9 activity, in various cell models, including MPTP- and rotenone-stressed MES23.5 dopaminergic cells (Dong et al., 2009; Yu et al., 2016), rotenone-burdened rat retinal ganglion cells (Liu et al., 2018) and primary hypothalamic neurons during oxygen and glucose withdrawal (Chung et al., 2007).

To unravel the pathways that drive mitochondrial biogenesis, the missing links can be derived from related studies that center on aging and caloric restriction, yet only indirectly use AG. In this context, AG has been deemed as the main neuroprotective factor that is secreted upon caloric restriction and is responsible for the survival-promoting activation of AMPK (Bayliss et al., 2016). In rodents, dietary restriction reduces oxidative stress, increases ATP production at a reduced cost of total oxygen, lowers mitochondrial membrane potential and drives mitochondrial biogenesis via the activation of peroxisome proliferator-activated receptor gamma coactivator 1-alpha (PGC1α) (Lopez-Lluch et al., 2006). PGC1α acts as a master regulator that induces mitochondrial biogenesis and upregulates the nuclear expression of mitochondria-related genes, such as the transcription factors NRF1, NRF2 and mitochondrial transcription factor A (TFAM). Subsequently, NRF1, NRF2, and TFAM initiate the replication of mtDNA, the transcription of mitochondrial respiratory genes and the synthesis of anti-oxidative proteins, for example glutathione peroxidase 1 and manganese superoxide dismutase (MnSOD). Jointly, the PGC1α-induced transcriptional changes in mitochondria-associated genes protect from MPTP oxidative assault in cell and animal models of PD, whereas the deletion of PGC1α exacerbates MPTP-induced injury and excitotoxicity (Scarpulla, 2002, 2006; Kang and Hamasaki, 2005; St.-Pierre et al., 2006; Mudo et al., 2012; Quan et al., 2020). The pro-mitochondrial impact of PGC1α in PD, whose expression levels were found to be decreased in the brains of PD patients, has been made evident in knockdown studies, in which the suppression or conditional knockdown of PGC1α led to the selective atrophy of dopaminergic neurons in the SNpc and lessened dopamine pools in the striatum of adult rodents (Shin et al., 2011; Jiang et al., 2016).

As illustrated in Figure 1, PGC1α is regulated by the coordinated actions of AMPK plus sirtuin 1 (SIRT1). And indeed, the latter 3 effectors are all activated by AG (Bayliss and Andrews, 2013). Initially, the canonical activation of GHS-R1α involves, but is not limited to, G_α*q*/11_ coupling to GHS-R1α, the induction of phospholipase C (PLC), the PLC-mediated turnover of phosphatidylinositol 4,5-bisphosphate (PIP2) into inositol triphosphate (IP3) and the liberation of calcium (Ca^2+^) from the intracellular ER stores by IP3. Additionally, Ca^2+^ influx by the non-canonical association of Gαs with GHS-R1α, followed by the activation of cAMP, PKA and opening of N-type Ca^2+^ channels have been reported. The induction of the cAMP/PKA pathway is highly debated and appears to be conditional and cell-type specific, however (Kohno et al., 2003; Yin et al., 2014; Yanagi et al., 2018). Furthermore, cAMP/PKA-signaling is evoked by the physiological release of AG-counteracting and growth-promoting hormones that are associated with nutrient abundance and increased glucose metabolism, such as insulin and leptin (Yang and Yang, 2016). Therefore, in general, AG induces intraneuronal Ca^2+^ accumulation in an IP3-mediated manner, leading to the activation of the AMPK-phosphorylating calmodulin-dependent protein kinase kinase-β (CaMKKß) (Hawley et al., 2005; Anderson et al., 2008). Activated AMPK further elevates PGC1α levels, directly phosphorylates PGC1α to promote promotor binding and raises intracellular nicotinamide adenine dinucleotide (NAD^+^) levels, leading to activation of the NAD^+^-sensitive deacetylase SIRT1 (Iglesias et al., 2004; Jager et al., 2007; Canto et al., 2009; Fujitsuka et al., 2016). Furthermore, AMPK phosphorylates glyceraldehyde 3-phosphate dehydrogenase (GAPDH) at Ser^122^ during starvation to encourage the nuclear trafficking of GAPDH and the displacement of SIRT1 from its repressor Deleted in Breast Cancer 1 (DBC1) (Chang et al., 2015). On the other hand, SIRT1 deacetylates and activates liver kinase B1 (LKB1), the second major AMPK-targeting kinase besides CaMKKß, indicating a reciprocal relationship between AMPK and SIRT1 (Lan et al., 2008). Moreover, SIRT1 is capable of shuttling between the cytoplasm and nucleus (Tanno et al., 2007) and deacetylates nuclear PGC1α to initiate mitochondrial biogenesis (Lagouge et al., 2006) as well as the transcription factor forkhead box protein O1 (FoxO1) to amplify the expression of PGC1α (Daitoku et al., 2003; Frescas et al., 2005; Nakae et al., 2008). AG was shown to induce the synthesis of FoxO1 in the hypothalamus (Lage et al., 2010) and, under conditions of cellular stress, FoxO1 drives the transcription of various anti-oxidant enzymes, such as the PGC1α-co-activated, mitochondrial MnSOD (St.-Pierre et al., 2006; Hsu et al., 2010; Tong et al., 2012). Besides the activation of PGC1α and FoxO1, SIRT1 also improves stress tolerance through deacetylation of other effector proteins, such as the inflammatory master regulator nuclear factor κB (NF-κB) (Yeung et al., 2004) or DNA repair factor Ku70, which was shown to scavenge pro-apoptotic Bax from mitochondria to support cellular survival (Cohen et al., 2004). The activation of the AMPK/Sirt1/PGC-1α/UCP2 pathway by AG, in a GHSR1α-dependent manner, has also been connected to the amelioration of oxidative stress, neuronal atrophy and functional decline in response to hypoxic-ischemic encephalopathy *in vivo*, emphasizing the neuroprotective impact of this pathway (Huang et al., 2019). As such, AG not only mitigates the stress-provoked ATP hyperproduction and the associated excessive generation of ROS by burdened mitochondria in a UCP2-driven manner, but also re-invigorates mitoprotective and mitochondrial biogenesis-inducing AMPK and PGC-1α signaling in AD and PD.

### A Possible Implication of Acylated Ghrelin in the Enhancement of Mitochondrial Fusion and Fission

Unsurprisingly, in accordance with general mitochondrial dysfunction, the efficiency of mitochondrial fusion and fission gradually declines during the aging process and is disturbed in neurodegenerative diseases (Liu et al., 2020). Cell and animal studies in AD models as well as post-mortem examinations of patients, although not always matching perfectly, signify that the transcription of fusion-enhancers (OPA1, mitofusin (Mfn)1/2) is attenuated and the expression or activity of fission-modulators (dynamin-related protein 1 (Drp1), mitochondrial fission 1 protein (Fis1) and S-nitrosylated Fis1) are aberrantly elevated. These alterations provoked mitochondrial hyperfission, neuronal injury and synaptic degeneration *in vitro* and *in vivo* (Wang et al., 2008, 2009; Cho et al., 2009). On the other hand, in Aβ-based AD models, the genetic deletion of the fission-inducer Drp1 rescued from mitochondrial fragmentation, the drop of mitochondrial membrane potential and ATP production, the generation of ROS *in vitro* and prevented the accumulation of lipid peroxidation products, beta-secretase 1 expression, the formation of amyloid plaques and cognitive decline in APPswe/PSEN1dE9 mice (Baek et al., 2017). Similarly, the pharmacological or genetic interference with Drp-1 or the overexpression of the fusion-enhancers Mfn2 and OPA1 ameliorated excessive mitochondrial fission and impaired ATP production in PINK1/Parkin-mutant cells (Lutz et al., 2009) and shielded against MPTP-driven mitochondrial fragmentation, the stimulation of the pro-apoptotic activity of p53, Bax and PUMA, dopaminergic neuron and nerve terminal loss as well as motor deficits, but not micro- and astrogliosis, in the murine SNpc (Filichia et al., 2016). In opposition to Aβ, the role of PD-associated α-synuclein is less evident. While mutant α-synuclein enhances mitochondrial fragmentation, impairs the mitochondrial respiratory activity and induces neuronal death by inducing the displacement of wt α-synuclein from the inner-mitochondrial membrane (Kamp et al., 2010; Nakamura et al., 2011; Guardia-Laguarta et al., 2014), it must be noted that wt α-synuclein, in fact, promotes fusion and its expression may be a compensatory and protective mechanism to prevent hyperfission in PD (Berthet et al., 2014; Guardia-Laguarta et al., 2014; Menges et al., 2017).

Recent reports suggest a possible role for AG in the regulation of the mitochondrial fission and fusion dynamics (Morgan et al., 2018). In general, caloric restriction, which enhances the plasma release of AG, favors mitochondrial fission, leading to an increase in the expression levels of Drp1 and Fis1, while not altering the transcriptional pools of fusion-modulators, such as Mfn1, Mfn2 or OPA1 (Khraiwesh et al., 2013). Moreover, mitochondrial toxins, such as the PD-poison rotenone, and the pharmacological stimulation of AMPK activity, independent of any mitochondrial damage, provoke mitochondrial fission. In the context of AMPK, mitochondrial fission factor (MFF) has recently been identified as a direct downstream target of AMPK and the AMPK-mediated activation of MFF leads to the induction of the fission-promoting Drp1 (Toyama et al., 2016).

Notably, the stimulation of PGC-1α in response to heightened energy expenditure has been linked to the transcriptional upregulation of the mitochondrial fusion-advocate Mfn2 in the skeletal musculature of mice (Soriano et al., 2006). It was also shown that the overexpression of PGC-1α opposed unloading-associated muscular atrophy in the murine hindlimbs and prevented the transcriptional decline of the fusion-imparting proteins Mfn1, Mfn2 and OPA1, therefore restoring mitochondrial defects by improving fusion (Cannavino et al., 2015). Importantly, in the context of PD, the rotenone-evoked mitochondrial fragmentation and dysfunction have been connected to impairments in the mitochondrial biogenesis, the decreased activity of TFAM and PGC-1α as well as deregulated mitochondrial fusion and fission, which was related to transcriptional alterations in Mfn2, OPA1, Drp1, and Fis1 in PC12 dopaminergic neurons. The application of PGC-1α siRNA as well as the overexpression of this mitochondrial effector confirmed that PGC-1α upregulates the synthesis of Mfn2, while suppressing the transcription of Drp1. On the contrary, the neuronal exposure to rotenone augmented p-Drp1 levels and promoted its translocation toward mitochondria to evoke fragmentation, which was exacerbated by the muting of PGC-1α and prevented through the overexpression of PGC-1α. The results of this study imply a primarily fusion-enhancing and fission-inhibiting function of PGC-1α under physiological conditions, while the induction of PGC-1α protects from stress-driven mitochondrial fragmentation in dopaminergic neurons (Peng et al., 2017).

Considerably, AG stimulates the activity of the fusion/fission-regulators AMPK and PGC-1α in GHS-R1α-expressing cells, including neurons (Bayliss and Andrews, 2013; Huang et al., 2019). Moreover, besides an impressive range of other mitoprotective effects, the ghrelin analogs JMV2894 and/or hexarelin suppressed excessive, cisplatin-triggered mitochondrial fission in the skeletal muscles of rats by reversing the upregulation of Drp1 and the downregulation of Mfn2, thus raising the Mfn2/Drp1 index back to the levels of control rodents (Sirago et al., 2017). This is in line with the Mfn2-upregulating and Drp1-impeding function of PGC-1α (Peng et al., 2017), suggesting that AG stimulates the AMPK/PGC-1α axis to ameliorate mitochondrial fragmentation in response to cellular stress (Sirago et al., 2017). Therefore, AG may guard against pathologic hyperfission in AD and PD. Nonetheless, future studies are necessary to confirm a fusion/fission-navigating function of AG in appropriate models of neurodegeneration.

### Acylated Ghrelin Navigates the Release of Lactate by Astrocytes

Interestingly, AG may coordinate bioenergetic communication between astrocytes and neurons. Using a combination of rodents and primary hypothalamic astrocyte culture, it was discovered that AG downregulates the expression of glucose transporter (GLUT)2, but not GLUT1 or GLUT3, increases the transcription of glutamate-aspartate transporters in a GHS-R1α-dependent manner, enhanced the expression of lactate dehydrogenase and glycogen phosphorylase, diminished the transcriptional levels of glutamine synthase and upregulated the lactate-transporter monocarboxylate transporter 4 (MCT4). Ultimately, the latter changes led to reduced glucose uptake, elevated glutamate uptake and the steadily rising lactate levels in the cell culture medium (Fuente-Martin et al., 2016). Although the latter study showed some inconsistencies, AG appears to trigger a physiological, metabolic switch in astrocytes to preserve glucose and curb its uptake by astrocytes during fasting. In exchange, AG appears to prime astrocytes toward glutamate and possibly glycogen metabolism to generate ATP, while encouraging the liberation of lactate as a powerful alternative energy source for neurons (Schurr et al., 1988). Thus, AG possibly supports the neuronal activity in face of AD/PD-associated bioenergetic deficiencies and glucose hypometabolism in the brain (Neth and Craft, 2017; Sweeney et al., 2018).

## Autophagy and Mitophagy

### Deficiencies in Autophagy and Mitophagy Promote the Accumulation of Amyloids and Defective Mitochondria in Alzheimer's and Parkinson's Disease

Classically, dysfunctional autophagy is a common trait shared by most neurodegenerative diseases. Due to the less efficient removal of waste proteins in neurons, deficits in autophagy are thought to encourage the accumulation of toxic and misfolded proteins, such as Aβ and Tau in AD as well as α-synuclein (Lewy bodies) in PD (Fujikake et al., 2018). As a side note, the genetic deletion of the autophagy modulators autophagy related (ATG)5 and ATG7 evoked the age-dependent formation of ubiquitinated, diffuse inclusions, severe neuronal atrophy and disturbances in motor function and coordination. Thus, impairments in autophagy induce neurodegeneration independent of amyloid accumulation in affected brain areas (Hara et al., 2006; Komatsu et al., 2006).

Immunohistological investigations in the brain tissue of AD patients suggest that early increases in the neuronal rate of autophagy compensate for the accumulation of waste products, whereas the lysosomal function (proteolytic enzyme activity) and the clearance of lysosomal vacuoles is gradually impaired. This results in the intraneuronal accumulation of non-degraded and amyloid-containing autophagosomes, co-localizing strongly with neurons that display intracellular Tau pathology and the relative loss of mitochondria and other organelles (Cataldo et al., 1994; Nixon et al., 2005). An important distinction to make is that the blockade of autophagy, as achieved with the inhibition of mTor, obviously slowed the rate of degradation, yet showed no major consequences. In contrast, the inhibition of lysosome-associated proteolytic enzymes was capable of producing an AD-like phenotype (Boland et al., 2008). Therefore, AD patients appear to show deficits in the fusion of waste-filled autophagic vacuoles with lysosomes and the intra-autophagosomal degradation process. Nonetheless, AD patients showed a massive decline in the transcriptional levels of the autophagy initiator Beclin-1 during early stages of AD and strategies that have aimed to enhance the degree of autophagy, such as the lentivirus-mediated expression of Beclin 1 or the autophagy-inducing blockage of mTor by rapamycin, have been successful in the purging of Aβ and Tau pathology in *in vitro* and *in vivo* models of AD (Pickford et al., 2008; Jaeger et al., 2010; Spilman et al., 2010; Majumder et al., 2011). Such findings indicate that autophagy-enhancing approaches must ensue early, since the mere upregulation of autophagy is insufficient at an advanced stage of AD, when insoluble and proteolysis-resistant aggregates have already formed in the brain (Majumder et al., 2011).

Analogical to AD, late-stage PD patients showed diminished levels of the LAMP1, LAMP2A, and heat shock cognate 70, which execute chaperone-mediated autophagy, yet displayed elevated LC3-II levels (symbolic for autophagosome accumulation) and α-synuclein inclusions in the SN pars compacta (SNpc) and/or the amygdala. This indicates that, similar to AD, autophagosomal efficiency is lost during PD, leading to the amassment of defective, waste-cluttered lysosomes and the failure of amyloid clearance (Chu et al., 2009; Alvarez-Erviti et al., 2010; Dehay et al., 2010). There is also evidence that proteins involved in autophagosome initiation and formation, for example LC3 or ULK1/2, are sequestered into Lewy bodies in the brain of PD patients (Tanji et al., 2011; Miki et al., 2016). However, the lentiviral overexpression of Beclin 1 or the utility of the autophagy-activator and mTor-inhibitor rapamycin rescued the apoptosis of dopaminergic neurons in response to the loss of proteasomal function or the accumulation of α-synuclein in cells and animals (Pan et al., 2008; Spencer et al., 2009). These findings propose that early pharmacological interventions to potentiate the rate of autophagy may be useful to prevent the harmful accumulation of amyloids, although such approaches, due to dysfunctions in the autophagy machinery, are less likely to succeed at more advanced stages of AD and PD. These later defects in autophagy are likely to be the accumulative result of general impairments in the neuronal metabolism, including mitochondrial defects, heightened oxidative stress and amyloid burden, glucose hypometabolism, diminished growth factor and insulin-signaling etc, indicating that multi-targeted therapeutic approaches are advantageous.

Notably, mitophagy poses a specialized form of autophagy that rids cells from defective, ROS-generating mitochondria. As expected, mitophagy is widely impaired in respective models as well as in the brains of AD and PD patients, while the selective pharmacological enhancement of mitophagy can reverse several other pathological aspects, such as the generation of insoluble Aβ, Tau hyperphosphorylation, neuroinflammation, neuronal atrophy and cognitive impediments (Fang et al., 2019; Liu J. et al., 2019).

### A Mitophagy-Enhancing Role of Acylated Ghrelin Has Been Strongly Indicated

Interestingly, ghrelin may improve mitophagy, an autophagy derivate involved in mitochondrial quality control and disposal of damaged mitochondria (Bayliss and Andrews, 2013). While ghrelin is often praised for its ability to promote mitophagy, little mechanistic research has been conducted. To our knowledge, there is only a single study that has truly confirmed a mitophagy-boosting function, showing that the administration of AG enhanced autophagy and led to the emergence of autophagosome-enclosed mitochondria at various stages of degradation in HL-1 cardiac muscle cells (Ruozi et al., 2015). At the time, Bayliss and Andrews also admitted that there is no direct evidence that AG activates or promotes the activity of the main mitophagy modulators Parkin or PTEN-induced kinase 1 (Bayliss and Andrews, 2013). Based on the current lack of studies, it can only be assumed that AG promotes mitophagy indirectly by generally enhancing cellular autophagy (see chapter 4.3 below) and reducing the mitochondrial generation of ROS in a UCP2-conveyed manner, thus avoiding the accumulation of dysfunctional mitochondria in the first place.

### Acylated Ghrelin Induces Autophagy in the Periphery and in the CNS

Indeed, AG's autophagy-enhancing features, as summarized in Figure 1, have only recently emerged in the literature. Nonetheless, there is abundant evidence that highlights ghrelin's autophagy-triggering and tissue-preserving function in peripheral tissue. AG-driven autophagy is dependent on the stimulation of AMPK, leading to increased levels of ATG5, ATG7, ATG12, and Beclin-1, lessened p62 levels (an autophagy marker that is adversely correlated with autophagy), an elevated microtubule-associated protein light chain 3 (LC3)-II/LC3-I ratio, which serves as a marker to quantify mature autophagosomes (Mizushima and Yoshimori, 2007), and improvements in the autophagic flux (demonstrative of the formation and degradation rate of autophagosomes in a given time frame) (Slupecka et al., 2012; Tong et al., 2012; Mao et al., 2015; Ruozi et al., 2015; Ezquerro et al., 2016; Wan et al., 2016; Xu et al., 2017).

In contrast, the cerebral induction of autophagy by ghrelin has only sparsely been investigated. Nonetheless, it was demonstrated that SH-SY5Y cells stably expressing mutant amyloid precursor protein (APP) exhibit elevated anti-apoptotic Bcl-2 levels, decreased caspase-3 and caspase-7 activities, increased proteasome activity and improved autophagy, as marked by increased Beclin-1, LC3-II and normalized p62 levels, upon treatment with AG. The cytoprotective effects of AG were attributed to its ability to improve crosstalk between proteasomal and autophagosomal pathways, leading to the enhanced clearance of the overexpressed APP/Aß fragments in this cell model (Cecarini et al., 2016). Another well-constructed study discovered that caloric restriction raises both mRNA and protein levels of NPY as well as ghrelin in rat cortical neurons, resulting in diminished phospho-mTor levels, increased LC3-II levels, decreased p62 pools and enhanced autophagic flux (Ferreira-Marques et al., 2016). Importantly, autophagy was independently achieved through the use of AG or NPY, respectively, whereas the individual administration of either GHS-R1α or Y_1_, Y_2_, or Y_5_ receptor antagonists were able to attenuate autophagy, suggesting a synergistic effect of both peptides. Although we will not further address the neuroprotective properties of NPY (see Li et al., 2019), it must be noted that AG was shown to raise the synthesis of NPY in hypothalamic and cortical neurons (Wren et al., 2002; Ferreira-Marques et al., 2016). As such, there is the need to clarify which peptide acts in what brain region and which autophagy-promoting pathways are activated by NPY or AG, respectively.

As it is the common consensus, (macro)autophagy is primarily controlled by the activity of mTor or, more precisely, mTOR complex (mTORC)1. In the absence of nutrients and in a cellular effort to maintain the status quo, the deactivation of mTORC1 is linked to the decreased activity of ribosomal protein S6 kinase beta-1 (S6K1)/S6 protein, the elevated activation of the transcription-repressor 4E-binding protein 1, the inactivity of eukaryotic translation initiation factor 4E and, thus, the overall decreased expression of proteins. Shut-down of the growth-facilitating mTORC1 pathway, however, promotes the activity of the unc-51 like autophagy activating kinase (ULK_1/2_) initiation complex, which launches autophagosome maturation and the cellular purging of waste products, such as Aß, Tau or α-synuclein (Huang and Manning, 2008; Ma and Blenis, 2009; Lan et al., 2017; Kaleli et al., 2020). Tuberous sclerosis (TSC)_1/2_ acts as a major regulatory switch for mTORC1-mediated growth vs. autophagy and, typically in response to stressful cellular conditions and starvation, the activating phosphorylation of the cytoplasmic energy-sensor AMPK results in the AMPK-mediated phosphorylation of TSC_1/2_ and the inhibition of mTORC1 (Inoki et al., 2003; Manning and Cantley, 2003; Demetriades et al., 2016). Additionally, a reciprocal connection between mTor and AMPK exists, in which the absence of nutrients promotes the AMPK-driven suppression of mTor and the activating phosphorylation of ULK1 at Ser^317^ and Ser^777^, whereas energetic abundance stimulates the inhibitory phosphorylation of ULK1 at Ser^757^ via mTor (Kim et al., 2011; Lan et al., 2017).

AG-evoked autophagy is mainly connected to the downstream activation of the phospho-AMPK/mTOR axis, as it has been well-described for the initiation of autophagy in peripheral tissue (Tong et al., 2012; Mao et al., 2015; Ruozi et al., 2015; Ezquerro et al., 2016; Xu et al., 2017). In contrast, the limited amount of cerebral studies with AG, at the very least, have verified the induction of autophagy via mTOR inhibition in cortical neurons (Ferreira-Marques et al., 2016). The stimulation of AMPK, which is a highly debated therapeutic option for the treatment of PD, is, in fact, responsible for the large majority of AG's neuroprotective effects, including (macro)autophagy, mitochondrial enhancement as well as the cellular safeguarding from oxidative stress and inflammation (Bayliss and Andrews, 2013; Curry et al., 2018). Besides AMPK, another important positive regulator of autophagy poses SIRT1 (Chen et al., 2020). The activation of SIRT1 via AG has been confirmed in the periphery (Fujimura et al., 2014; Tamaki et al., 2015; Fujitsuka et al., 2016; Yang et al., 2016) as well as the hypothalamus in adult rodents and mouse models of aging (Velasquez et al., 2011; Fujitsuka et al., 2016). Indeed, SIRT1 is not only elevated upon treatment with AG, but interference with AMPK/SIRT1 signaling prevented the induction of autophagy in lymphoblastic leukemia cells (Heshmati et al., 2020). It has also been reported that SIRT1 directly deacetylates Tau protein at Lys^174^ and the viral delivery of SIRT1 to the hippocampus of SIRT1-deficient and P301S Tau transgenic mice attenuated the cerebral propagation of Tau (Min et al., 2018). AG-upregulated and SIRT1-activated and FoxO1 is well-known in aging research, responsible for the transcription of ATG genes and the mTor-suppressor Sestrin 3, therefore encouraging autophagy (see Figure 1 for an illustration of the discussed pathways) (Lage et al., 2010; Zhang et al., 2015).

Interestingly, in some instances, AG induces counterintuitive signaling pathways and stimulates neuroprotective Akt, which is an mTor-activator. The AG-driven induction of these discrepant signaling cascades seem to be highly conditional for preventing neuronal apoptosis during cerebral ischemia and excitotoxicity, however, and may be linked to the upregulation of other growth factors, such as IGF-1 (Frago et al., 2011; Spencer et al., 2013). In any case, the current evidence suggests that AG augments the neuronal rate of autophagy by inducing AMPK-signaling to inhibit mTor and upregulating the expression of various autophagy-implementing effectors to degrade amyloids, such as Aβ, in AD and PD. Since AG improves other pathologic areas, for instance mitochondrial dysfunction (chapter 3.2), insulin resistance and glucose hypometabolism (chapter 7.2) in neurons, AG may further ameliorate the functional deficits in autophagy that occur during later stages of AD and PD.

## Inflammation

### Systemic Inflammation in Alzheimer's and Parkinson's Accelerates Disease Progression

The detrimental impact of the neuroinflammatory pathology, which is believed to commence decades before the appearance of any symptoms, is widely acknowledged in AD and PD. While beneficial in the healthy brain, neurodegenerative conditions provoke a chronic shift of microglia as well as astrocytes from the supportive M2 to the pro-inflammatory M1 state, resulting in the release of various pro-inflammatory cytokines, including TNF-α, IFN-γ, IL-1ß, IL-6, and IL-12, and chemokines, for example the immune cell-recruiting monocyte chemoattractant protein 1 (MCP-1), the generation of excessive amounts of ROS and nitric oxide (NO) as well as the secretion of glutamate. Over time, prolonged neuroinflammation encourages various other secondary complications, such as impairments in protein degradation, amyloid misfolding, Tau hyperphosphorylation (in conjunction with the inflammation-perpetuating activation of the inflammasome), permeabilization of the BBB, peripheral immune cell infiltration into the CNS, mitochondrial dysfunction, cerebral insulin resistance, injury of the axonal myelin sheath and oligodendrocytes (evident in AD, yet less clear in PD), axonal transport deficiencies, synaptic damage, and, ultimately, widespread neuronal apoptosis (Gonzalez et al., 2014; Najem et al., 2014; Chen et al., 2016; Wang S. S. et al., 2018; Ising et al., 2019). Microglia may be stimulated by the Toll-like receptor (TLR)-mediated recognition of bacterial and viral particles, for example lipopolysaccharides (LPS) (Boche et al., 2013), the TLR2-driven interaction with α-synuclein (Kim et al., 2013), TLR2/4-binding to Aß (Reed-Geaghan et al., 2009), and serum-derived or locally released TNF-α and IFN-γ, whose combinatorial action was shown to be a crucial inflammatory mediator of dopaminergic cell death in a rodent model of PD (Mir et al., 2008; Barcia et al., 2011). In this context, some genetic variants of TLR4 have been linked to AD and the increased expression of TLR2 has been identified in AD models (Balistreri et al., 2009; Letiembre et al., 2009), whereas the enhanced transcription of TLR2 and TLR4 have been detected in α-synuclein and MPTP mouse models of PD (Panaro et al., 2008; Letiembre et al., 2009), indicating that immune regulation is harmfully altered in AD and PD. Likewise, astrocytes may be provoked by TLR2/4/5/6 receptor ligands, Aß or α-synuclein as well as microglia-derived cytokines, in particular the key stimulatory agents IFN-γ and TNF-α (Johnstone et al., 1999; Bezzi et al., 2001; Lee H. J. et al., 2010; Barcia et al., 2011; Ma et al., 2013).

Notably, there are other inflammatory triggers besides amyloids in AD and PD. More precisely, fragments derived from apoptotic neurons, termed damage-associated molecular patterns (DAMPs), are capable of stimulating inflammatory cascades via interaction with TLRs or receptors for advanced glycation endproducts on microglia. DAMPs, of course, include Aß, Tau and α-synuclein, but also encompass many more, such as myelin debris, neuron-specific enolase (a glycolytic enzyme), S100 calcium-binding protein β (S-100ß) (an astroglial modulator), advanced glycation end products and many more. Furthermore, pathogen-associated molecular patterns (PAMPs) that originate from cerebral infections, such as LPS, or, in the case of AD, infections with members of the Herpesviridae family and Hepatitis C virus, may further potentiate neuroinflammation (Morales et al., 2014; Sochocka et al., 2017; Cortes et al., 2018; Stephenson et al., 2018).

Importantly, inflammation is not limited to the brain in AD and PD, but is potentiated by multiple inflammatory mechanisms in the periphery. First, the presence of heightened levels of pro-inflammatory cytokines in the blood stream can be sensed by the CNS through the so-called gut-brain axis, also known as the “vagal reflex.” The latter involves the intestinal monitoring of the peripheral inflammatory status by the efferent ends of the vagus nerve. In the presence of abnormally elevated levels of pro-inflammatory cytokines in the blood stream or following gut microbial inflammation, the vagal nerve signals to the nucleus tractus solitarius (NTS), a major signaling hub located in the brain stem, that receives input from multiple peripheral organs. The NTS, on the other hand, further projects across the entirety of the CNS, ultimately leading to the intestinal return of immune-suppressing signals through the afferent ends of the vagal nerve. It has been proposed that chronic inflammation provokes NTS dysfunction, which, in turn, propagates neuroinflammation and death across the brain in AD (Daulatzai, 2012; Wang J. T. et al., 2018). Moreover, intestinal inflammation and injury are strikingly pronounced prior to the onset of AD and PD, which, besides the additional inflammatory burden, induce the leakage of Aβ- and α-synuclein-like amyloids that may cross the enteric nervous system (ENS), enter the brain and stimulate cross-seeding (Ambrosini et al., 2019). Second, blood-borne pro-inflammatory cytokines/chemokines, PAMPs and DAMPs may access the CNS directly or indirectly, by promoting BBB damage and leakage. In cooperation, the cerebral and blood stream-derived inflammatory agents induce neuronal death, kill oligodendrocytes, injure the axonal myelin sheath, evoke atrophy of the neuronal projections and lead to the assassination of astrocytes, further weakening the integrity of the BBB (Sankowski et al., 2015). Notably, metabolic and vascular disorders provoke chronic low-grade inflammation in the peripheral system that, as anticipated, contribute to the development of AD and PD (Chen et al., 2016). Third, as a consequence of BBB permeabilization, immune cell infiltration is encouraged. DAMPs, such as aggregated amyloids or fragments of apoptotic neurons, may reach the circulatory stream through the lymph nodes or following BBB breaching, while Aß may also be drained at perivascular and leptomeningeal spaces. Subsequent peripheral inflammatory responses by antigen-presenting cells and lymphocytes (T-cells) then induce immune entry into the CNS in AD and PD (Fisher et al., 2011; Anderson et al., 2014).

Regarding immune infiltration, CD4^+^ T-cell-deleted mice were shown to be protected from MPTP-triggered neurodegeneration, proposing that the adaptive immune system is heavily involved in PD pathology (Brochard et al., 2009). Further *in vivo* studies in the MPTP model support the idea that, in conjunction with BBB injury and the loss of tight junction proteins in the nigrostriatal area, the pathological infiltration of lymphocytes and other immune cells as well as the T-cell-driven induction of microglia occur in the SN (Kurkowska-Jastrzebska et al., 1999; Chao et al., 2009; Reynolds et al., 2010; Depboylu et al., 2012). Moreover, it has been strongly implied that CD4^+^ T helper (Th)1 and Th17 cells, for instance immunoreactive against α-synuclein, are the main lymphocyte populations that contribute to the death of dopaminergic neurons (Brochard et al., 2009; Reynolds et al., 2010). Moreover, the invasion of CD4^+^ and CD8^+^ T-cells has been confirmed in post-mortem brain tissue of PD patients, co-localizing with lesioned brain regions (Brochard et al., 2009). A clinical investigation concluded that the quantity of serum CD4^+^ T-cells was correlated to the PD disease score and the functional impairment of T-cell suppressing regulatory T-cells (Tregs) was identified in the blood of PD patients (Saunders et al., 2012). In addition, heightened numbers of partially α-synuclein-reactive Th17 cells were discovered in the blood of PD patients (Sulzer et al., 2017); to the degree that PD has been postulated as an α-synuclein-reactive autoimmune disease (Benner et al., 2008; Hu, 2011). Unsurprisingly, in line with the encouraged cerebral trespassing of lymphocytes, BBB leakage has been confirmed in the brains of PD patients (Kortekaas et al., 2005; Pisani et al., 2012). A similar pathologic role for T-cells has been implied in AD. For example, the long-term administration of low-doses of IL-2, believed to assist the activity of Tregs (Klatzmann and Abbas, 2015), enhanced the levels of Tregs in the rodent brain, improved the Aß_42/40_ ratio, stimulated the clearance of Aß plaques, elevated LTP, attenuated spinal degeneration and reversed memory impediments in the APP/PS1ΔE9 mouse model of AD (Alves et al., 2017). Additionally, altered adaptive immune mechanics have been observed in the CNS of several Aß-based *in vivo* models of AD, displaying hippocampal BBB disruption, the infiltration of peripheral monocytes/macrophages, neutrophils and CD4^+^ T-cells (predominantly Th1 and Th17) as well as the increased transcription of pro-inflammatory cytokines, such as IFN-γ and IL-17, and chemokines, including MIP-1α (a macrophage attractant) plus CXCL1 (implicated in neutrophil recruitment) (Browne et al., 2013; Zhang J. et al., 2013; Minogue et al., 2014). In accord with the animal studies, blood profiling of AD patients indicated heightened adaptive immune responses, such as a total reduction in naive T-cells, a tendency of T-cells to differentiate into CD4^+^ subsets or the elevated activity of pro-inflammatory CD4^+^ Th17 cells (Shalit et al., 1995; Richartz-Salzburger et al., 2007; Speciale et al., 2007; Larbi et al., 2009; Saresella et al., 2011). Another study further concluded that CD4^+^ T-cell counts might be correlated to AD severity (Shalit et al., 1995).

**Figure 2:**
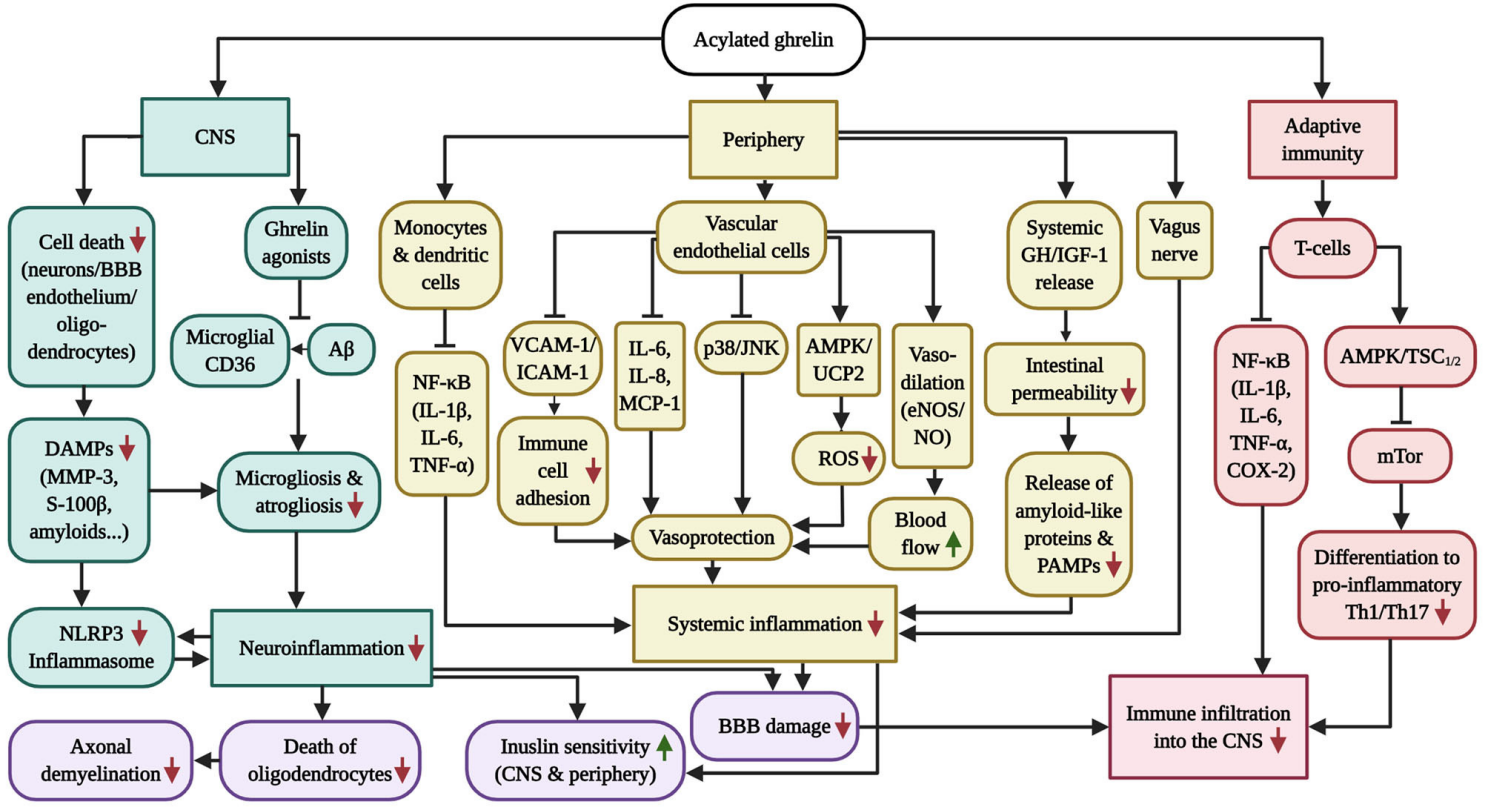
Overview of the anti-inflammatory capabilities of GHS-R1α receptor activation. AD and PD are characterized by chronic systemic inflammation, which includes micro-/astrogliosis and inflammasome activation following the accumulation of amyloids and DAMPs in the CNS, vagus nerve and intestinal (microbiome) inflammation in the periphery as well as pathologic CD4^+^ T-cell infiltration into the brain, which is exacerbated by the inflammation-driven injury of the BBB and vasculature. While AG has successfully prevented neuroinflammation in AD and PD models, the diagram further illustrates the beneficial effects of AG on inflammasome induction, peripheral inflammation and adaptive immunity in other inflammatory disease models, which culminate in vascular protection as well as enhanced blood flow, BBB stability, insulin sensitivity, oligodendrocyte survival and axonal myelination. Of note, GHS-R1α does not appear to be expressed by microglia, suggesting that the anti-inflammatory benefits of AG in the CNS are indirect. Ghrelin agonists offer the additional benefit of blocking microglial CD36, thus inhibiting Aβ-elicited inflammation.

Intriguingly, AG is capable of preventing the latter described, AD/PD-associated cerebral, peripheral and adaptive immune alterations, as condensed in Figure 2. The following chapters will investigate these anti-inflammatory characteristics of AG in greater detail. Although many of the subsequent studies were not conducted in AD or PD models, they serve as a proof of principle to emphasize that AG functions as a potent systemic immunosuppressant, independent of the underlying inflammatory context.

### Neuroinflammation

#### Acylated Ghrelin Abrogates Neuroinflammation Indirectly by Preventing the Apoptosis of Cerebral Cells

Indeed, there is abundant evidence that the utility of AG curbs pro-inflammatory responses in the CNS. A series of *in vivo/ex vivo* studies has given clear indication that AG prevents microgliosis, astrogliosis and/or the cerebral expression of pro-inflammatory cytokines in animal models of AD, in which Aβ was the primary inflammatory stimulator (Moon et al., 2011; Dhurandhar et al., 2013; Santos et al., 2017; Jeong et al., 2018), in the MPTP-induced PD rodent model (Moon et al., 2009a), following various forms of ischemic CNS/spinal cord injury (SCI) in rodents (Ersahin et al., 2010, 2011; Lee J. Y. et al., 2010; Cheyuo et al., 2011; Kenny et al., 2013; Lee et al., 2014b, 2015), as well as drug-induced excitotoxicity (Lee J. et al., 2010; Lee et al., 2012). However, despite AG's well-established, anti-inflammatory actions in the CNS, *in vitro* and *in vivo* investigations have confirmed that neither brain- or spinal cord-resident microglia, cultured BV-2 microglial cells nor primary microglia express GHS-R1α (Moon et al., 2009a; Lee J. Y. et al., 2010; Lee and Yune, 2014). In the case of astrocytes, it was reported that AG directly decreases the secretion of tumor necrosis factor alpha (TNF-α) by cultured hypothalamic astrocytes, although the hormone stimulated the liberation of interleukin (IL)-6 (Garcia-Caceres et al., 2014).

Instead of a direct, immunosuppressive effect on microglia and astrocytes, the majority of studies suggest that AG operates in an indirect manner and restrains neuroinflammation through its cytoprotective properties in neurons and other cerebral cells. For instance, in the MPTP mouse model of PD, AG attenuated microglial induction, reduced the expression of IL-1 and TNF-α and diminished nitrotyrosine and NO levels in the SNpc, which protected local dopaminergic neurons and striatal projections from neurotoxic assault (Moon et al., 2009a). Importantly, while the authors verified the absence of GHS-R1α on microglia, the use of GHS-R1α-antagonists fully ablated the anti-inflammatory and protective effects of AG, highlighting that there must be a GHS-R1α-attributed, yet indirect, mechanism at work that inhibits pro-inflammatory immune responses. Interestingly, a cell culture study demonstrated that the reduced microglial activation following AG-treatment was linked to the downregulation of matrix-metalloproteinase 3 (MMP-3) by co-cultured, dopaminergic neurons (Moon et al., 2009a). Likewise, AG guarded bEnd.3 microvascular endothelial cells from oxygen-glucose deprivation/reoxygenation *in vitro*, hippocampal neurons from kainic acid as well as spinal cord neurons from mechanical injury *in vivo*, thus forestalling the release of MMP-3 by apoptotic cells. This, in a MMP-3-dependent manner, resulted in diminished microglial inflammation (Lee J. et al., 2010; Lee et al., 2015). Indeed, MMP-3, typically originating from apoptotic neurons, but also dying astrocytes and endothelial cells, is a well-known inflammatory stimulator of microglia that evokes superoxide production and the microglial secretion of TNF-α, IL-1β and IL-6. In a reciprocal manner, inflammation incites the microglial expression and liberation of MMP-3, initiating a wicked cycle of neuronal degeneration and neuroinflammation (Kim et al., 2005; Kim and Hwang, 2011). Besides MMP-3, *in vitro* studies have demonstrated that AG suppressed the LPS-induced secretion of IL-6 in mouse dopaminergic SN4741 cells and the MPTP-enforced induction of the inflammatory master regulator NF-κB in mouse dopaminergic MES23.5 cells. The latter was further accompanied by the attenuated formation of the oxidative stress marker malonaldehyde, the normalization of the transcriptional levels of the anti-oxidative enzymes SOD and catalase as well as the upregulation of the Bax/Bcl-2 ratio, symbolic for the protection from neuronal apoptosis (Liu et al., 2010; Beynon et al., 2013). As such, the existing evidence points toward an inflammation-suppressing and survival-enhancing function in non-microglial cerebral cells that is indirectly linked to the reduced liberation of inflammation-stimulating damage associated molecular patterns (DAMPs), such as MMP-3.

#### The Acylated Hormone Ghrelin Rescues Oligodendrocytes and Prevents Demyelination

It must be mentioned that AG guards oligodendrocytes, the exclusively myelinating cell type in the brain. As confirmed with the utility of GHS-R1α, ERK and p38 inhibitors, an *in vitro* study showed that the interaction of AG with GHS-R1α on oligodendrocytes shields the cells from hydrogen peroxide (H_2_O_2_) and apoptosis by potentiating oligoprotective ERK signaling, while attenuating the pro-apoptotic activation of p38 (Lee et al., 2011). Another cell culture study emphasized that AG is capable of blocking the LPS-provoked inflammatory stimulation of the p38 and c-Jun N-terminal kinase (JNK) stress kinase pathways, the release of pro-nerve growth factor and the generation of ROS by BV-2 microglial cells, thus protecting co-cultured oligodendrocytes from death by oxidative assault (Lee and Yune, 2014). Collectively, previous research has demonstrated that AG rescued oligodendrocytes from inflammatory and oxidative damage, therefore protecting the integrity of myelinated axons in *in vivo* models of SCI and MS (Lee J. Y. et al., 2010; Lee et al., 2011; Liu F. et al., 2019). As such, the utility of AG may be useful to ameliorate the age-associated myelin pathology in neurodegenerative diseases (Wang S. S. et al., 2018), yet further investigations in the context of AD and PD are necessary.

#### Ghrelin Agonists May Suppress Microglial Inflammation by Binding to CD36

Notably, human fetal microglia, N9 microglial cells as well as microglia resident in the AD and non-AD brain, along with monocytes, macrophages and endothelial cells, were shown to express a GPCR known as cluster of differentiation 36 (CD36). This receptor has been reported to act as an inflammatory conductor for Aß, leading to the production of ROS and pro-inflammatory cytokines upon the interaction of fibrillar Aß with microglial or macrophage CD36 (Coraci et al., 2002; Bamberger et al., 2003; El Khoury et al., 2003; Demers et al., 2004). Interestingly, a receptor binding site for hexarelin, a synthetic DAG analog, was identified on CD36 (Demers et al., 2004). Furthermore, a study uncovered that DAG, but not AG, was capable of binding to CD36 receptors on cultured N9 cells, preventing fibrillar Aß_25−35_-triggered release of IL-1ß and IL-6 (Bulgarelli et al., 2009). Since anti-CD36 antibodies strongly attenuated N9 microglial H_2_O_2_ production (Coraci et al., 2002), it is likely that the binding of DAG sterically hinders the pro-inflammatory interaction of CD36 with Aß (Bulgarelli et al., 2009).

Intriguingly, some ghrelin agonists show affinity toward both GHS-R1α and CD36, for example hexarelin or GHRS-6 (Demers et al., 2004; Berlanga-Acosta et al., 2017). Hexarelin was demonstrated to interact with both GHS-R1α and CD36 on cultured THP-1 monocytes and primary peritoneal macrophages derived from apoE^−/−^ mice (Avallone et al., 2006). Moreover, the prolonged daily injection of the CD36-favoring ghrelin derivate EP 80317 dramatically ameliorated the development of vascular lesions in the apoE^−/−^ animal model of arteriosclerosis by lessening the CD36-driven endocytosis of oxidized low density lipoprotein (oxLDL) by macrophages (Marleau et al., 2005). Thus, in direct comparison to the GHS-R1α-binding AG, it is tempting to speculate that GHS-R1α/CD36 co-binding ghrelin analogs may be a superior choice for the amelioration of Aβ-driven microglial inflammation and ROS-production in AD.

#### Evidence that Acylated Ghrelin Opposes the Activation of the Inflammasome in the Brain

The stimulation of the inflammasome and the associated pyroptosis, the “fiery death” of microglia, oligodendrocytes and other cells, a relatively recent upbringing, have been identified as major drivers of neuroinflammation, demyelination and degeneration of the spinal cord during MS (McKenzie et al., 2018). The nod-like receptor protein 3 (NLRP3) inflammasome-associated propagation of neuroinflammation has also recently been identified in AD and PD, believed to sequentially involve Aß accumulation, the Aß-triggered inflammasome activation, inflammasome-induced cytokine production and the onset of Tau pathology in AD (Mamik and Power, 2017; Ising et al., 2019; Stancu et al., 2019).

Interestingly, in the experimental autoimmune encephalomyelitis (EAE) mouse model, AG not only inhibited microglial immunoreactivity, the activating phosphorylation of NF-κB and the associated synthesis of various pro-inflammatory cytokines in the spinal cord, but also prevented the activation of the NLRP3 inflammasome complex and pyroptosis in the spinal cord of EAE mice. Indeed, the transcriptional levels of the inflammasome components NLRP3 and caspase-1, the pyroptosis-inducer gasdermin D as well as the inflammasome-derived cytokines IL-1ß and IL-18 were drastically reduced in AG-treated EAE mice, resulting in ameliorated behavioral symptoms (Liu F. et al., 2019). In this context, AG was reported to obstruct the activation of NF-κB in the spinal cord of the EAE animal model, in cultured dopaminergic neurons and in primary human T-cells (Dixit et al., 2009; Liu et al., 2010; Liu F. et al., 2019), with NF-κB driving the synthesis of the inflammasome sensor NLRP3 as well as the pro-inflammatory cytokines pro-IL-1/IL-1, pro-IL-18, TNF-α and many more (Afonina et al., 2017). Moreover, AG downregulated the transcription of IL-1 and/or the inflammasome-activating cytokine TNF-α in face of MPTP-injury (PD), threohydroxyaspartate (THA)/kainic acid-assault (excitotoxicity), subarachnoid hemorrhage and SCI (Moon et al., 2009a; Ersahin et al., 2010; Lee J. et al., 2010; Lee et al., 2012, 2014b; Alvarez and Munoz-Fernandez, 2013). As such, AG is adept in blocking the initial steps necessary for NLRP3 inflammasome induction, as observed in the EAE-based study of Liu F. et al. (2019). While it is not entirely evident how AG inhibits NF-κB signaling in GHS-R1α-negative microglia (Moon et al., 2009a; Lee J. Y. et al., 2010; Lee and Yune, 2014), it can be assumed that the prevention of neuronal and oligodendrocyte death, leading to the reduced liberation of DAMPs, indirectly avert inflammatory processes, the sensing of DAMPs by NLRP3 and other inflammasome conductors and, thus, inflammasome formation (see also chapter 5.2.1).

### Peripheral Inflammation

#### Acylated Ghrelin Suppresses Inflammatory Responses in Mononuclear Phagocytes and Quenches Peripheral Inflammation *in vitro* and *in vivo*

Cell culture studies have indicated that AG exerts direct anti-inflammatory actions in the peripheral mononuclear phagocyte system. The expression of GHS/R1α has been confirmed in the murine RAW264.7 macrophage-like cell line as well as in primary immature and mature monocyte-derived dendritic cells of human origin (Dixit et al., 2004; Waseern et al., 2008). Furthermore, *in vitro* studies have shown that the administration of AG downregulated the synthesis of IL-1β, IL-6, and TNF-α in human peripheral blood mononuclear cells following irritation with the mitogen phytohemagglutinin (Dixit et al., 2004). Also, AG dose-dependently blocked the transcription of pro-inflammatory cytokines via the inhibition of NF-κB in LPS-induced RAW264.7 mononuclear cells in a GHS-R1α-dependent manner. Interestingly, AG evoked NF-κB-independent p38 signaling in these cells as well, promoting the secretion of the anti-inflammatory cytokine IL-10 (Waseern et al., 2008). As such, AG dampens the production of pro-inflammatory mediators by mononuclear cells, while encouraging the liberation of anti-inflammatory cytokines. In concert, AG ameliorated the LPS-driven systemic accumulation of pro-inflammatory IL-1β, IL-6, and TNF-α in the plasma, spleen, liver, lungs and lymph nodes, thus protecting mice from endotoxic shock (Dixit et al., 2004).

Over the previous two decades, AG has consistently performed well in animal models of various inflammatory conditions, guarding against endotoxemia/sepsis, pancreatic, hepatic and kidney disease, cardiovascular conditions, arteriosclerosis, colitis, arthritis, age-induced inflammation and more (Baatar et al., 2011; Deboer, 2011). For instance, the administration of AG succeeded in the animal model of colitis, showing downregulated local and systemic release of pro-inflammatory modulators, reduced inflammatory Th 1 activity, elevated action of immunosuppressive regulatory T-cells (Tregs), diminished oxidative stress, ameliorated intestinal tissue loss and reinvigorated mucosal vitality (Gonzalez-Rey et al., 2006; Konturek et al., 2009; Pamukcu et al., 2013; Matuszyk et al., 2015; Ceranowicz et al., 2017). Anti-inflammatory properties of AG have also been confirmed in various clinical studies (Kodama et al., 2008; Takata et al., 2015; Farokhnia et al., 2020). As concluded elsewhere in the context of colitis, the inflammation-ameliorating mechanisms of AG include (i) the attenuation of systemic innate and adaptive immune responses, which is dependent on the direct suppression of leukocytes; (ii) the AG-stimulated liberation of tissue-strengthening GH and insulin-like growth factor 1 (IGF-1) and (iii) the elevation of the intestinal blood flow and motility, thus reducing the contact time of inflammatory irritants with the intestinal mucosa (Baatar et al., 2011; Deboer, 2011). Considerably, intestinal damage, leakiness and inflammation co-occur in AD and PD, preceding the manifestation of neurodegenerative processes. The early inflammatory shift in the gut encourages the release of pro-inflammatory cytokines and chemokines, bacterial stimulants (i.e. LPS) as well as aggregation-prone amyloid-like proteins into the blood stream. The inflammatory stress, combined with the suspected trafficking of intestinal Aβ and α-synuclein seeds across the ENS into the CNS, is thought to instigate amyloid deposition and neuronal atrophy (Ambrosini et al., 2019). As such, AG's beneficial actions in the gut must not be underestimated.

#### The Vasoprotective and Blood Flow-Enhancing Properties of Acylated Ghrelin

In addition to modulating the monocyte system, AG protects the endothelial vasculature and stimulates vasorelaxation to enhance blood flow. Immunohistochemical examinations of human tissue have confirmed the plentiful presence of GHS-R1α on endothelial cells of various myocardial, but also pulmonary, renal and adrenal blood vessels, whereas the receptor is sparsely expressed by the blood vessel endothelium that supplies nerves and connective tissue (Kleinz et al., 2006). Although to a low degree, GHS-R1α is also expressed throughout the cerebral vasculature and a markedly high density of GHS-R1α has been detected in the microvasculature of the granular layer of the cerebellum (Katugampola et al., 2001; Ku et al., 2015, 2016).

In cell culture studies using human umbilical vein endothelial cells (HUVECs), it was demonstrated that AG inhibited the nuclear translocation of NF-κB even in the absence of inflammatory stimuli, quenched the basal and H_2_O_2_-triggered release of IL-8 and oxLDL-encouraged release of IL-6, blocked the endothelial expression of the immune cell-recruiting monocyte chemoattractant protein 1 (MCP-1) and reduced the TNF-α-incited adhesion of co-cultured monocytes/macrophages to the vascular endothelial cells, which was presumably related to the endothelial downregulation of vascular cell adhesion molecule-1 (VCAM-1) and intercellular adhesion molecule 1 (ICAM-1) (Li et al., 2004; Zhang, 2017). Since DAG failed to modify inflammatory reactions by HUVECs, it was implied that the described inflammation-dampening effects were reliant on GHS-R1α (Li et al., 2004). Moreover, a recent study revealed a cytoprotective function of AG in palmitate- and glucose-stressed human microvascular endothelial cells, in which AG rescued apoptosis and caspase-3 activity by inhibiting the stress kinases p38 and JNK1/2, diminishing the mitochondrial generation of ROS and normalizing the rate of oxygen consumption and ATP production (Liao et al., 2017). Additionally, clinical studies indicate that AG, in an endothelial nitic oxide synthase (eNOS)-mediated and GH-independent manner, enhances the bioavailability of NO and elicits vasorelaxation, hence improving blood flow and decreasing blood pressure (Nagaya et al., 2001, 2004; Shimizu et al., 2003; Tesauro et al., 2005; Kleinz et al., 2006; Virdis et al., 2015). Since AG enhances AMPK activity in endothelial cells (Fang et al., 2013) and the stimulation of AMPK was proven to trigger the AMPK-conveyed activating phosphorylation of eNOS at Ser^1177^ in cultured human and rat endothelial cells, thus strengthening vasodilation *in vivo* (Morrow et al., 2003; Suzuki et al., 2008), it is highly likely that the AG-evoked liberation of NO is AMPK-mediated. Thus, in GHS-R1α-expressing blood vessel endothelial cells in the periphery, AG protects the vasculature by inducing the mitochondrial ROS-scavenger UCP-2 to ameliorate oxidative stress, inflammatory responses and vascular insult by hyperglycemia and hyperlipidemia. Moreover, by increasing blood flow, AG might ameliorate the pathologically diminished cerebral blood flow and deficits in the CNS delivery of glucose that have been detected in the brains of AD (Lyingtunell et al., 1981, Eberling et al., 1992; Ogawa et al., 1996; Roher et al., 2012; de Eulate et al., 2017) and PD (Huang et al., 2008; Hosokai et al., 2009; Liepelt et al., 2009; Borghammer et al., 2010; Berti et al., 2012) patients.

#### Acylated Ghrelin Stimulates Anti-inflammatory Signaling Across the Vagus Nerve

Importantly, various investigations have indicated that AG controls peripheral inflammation via the vagal nerve system. In concert, the area postrema, the nucleus tractus solitarius and the dorsal motor nucleus of the vagus (DMV) form the dorsal vagal complex (DVC) that serves as a commanding platform for the autonomic nervous system, navigating gastrointestinal motility, secretory activity and pancreatic hormone release (Price et al., 2008; Mussa and Verberne, 2013). Interestingly, GHS-R1α is expressed in the DVC and the plasma GH pools and c-Fos immunoreactivity in the DVC were found to decline with age in Fischer344 rats. The injection of GH, on the other hand, was capable of raising the transcriptional levels of GHS-R1α and partially re-established c-Fos immunoreactivity in the DVC of these aged rodents, suggesting that the GH-induced expression of GHS-R1α regulates the vagal sensitivity toward AG. Moreover, the administration of LPS into older animals, which display lessened expression of GHS-R1α in the DVC, evoked the excessive release of TNF-α and IL-6, far greater than in younger littermates (Wu et al., 2009b). In agreement, the utility of a GHS-R1α antagonist exacerbated the endotoxemia-induced liberation of pro-inflammatory cytokines into the blood stream in young rats (Wu et al., 2009b), implying an important immunosuppressive function of AG within the vagal nerve system that gradually deteriorates with age.

Besides the DVC, the presence of GHS-R1α was also discovered on the nodose ganglion of the vagus nerve as well as the nerve terminals of the outgrowing afferent vagal fibers, which innervate the digestive tract and sense the systemic conditions and circulatory hormone levels. The binding of plasma AG to GHS-R1α on the afferent vagal ends mutes vagal firing, contributing to the initiation of feeding and GH-release (Date, 2012). In the context of inflammation, it is broadly accepted that the afferent vagal nerves exert anti-inflammatory (cholinergic) signaling via multiple mechanisms following stimulation (see Bonaz et al., 2016). As confirmed with vagotomy, the interaction of administered AG with the vagal nerve not only quenched systemic inflammation during sepsis (Wu et al., 2007, 2009a), but also suppressed inflammation in *in vivo* models of traumatic brain injury, focal cerebral ischemia and gut ischemia/reperfusion injury, thus attenuating the accumulation of plasma and cerebral inflammatory cytokines (Wu et al., 2008; Bansal et al., 2010, 2012; Cheyuo et al., 2011), inflammation-driven intestinal permeabilization and atrophy (Wu et al., 2008, 2009a; Bansal et al., 2010).

### Adaptive Immunity and CNS Infiltration

#### Acylated Ghrelin Suppresses Pro-inflammatory T-Cells and Blocks Immune Cell Invasion Into the Brain

AG is also involved in the regulation of the adaptive immune system, which is based on the modulation of T-cells. It was confirmed that primary human blood mononuclear cells and human T-cells express GHS-R1α and the treatment with AG counteracted the leptin-induced secretion of the pro-inflammatory cytokines IL-1, IL-6 and TNF-α by these cells *in vitro* (Dixit et al., 2004). In agreement with this, the injection of AG prevented the LPS-stimulated and T-cell-instructed production of IL-1β, IL-6, and TNF-α in various organs and the blood plasma, thus ameliorating anorexia in the *in vivo* endotoxemia model (Dixit et al., 2004). In a follow-up study, the same group demonstrated that AG restrains the production of various cytokines by inhibiting the nuclear translocation of NF-κB and the expression of pro-inflammatory genes. Interestingly, AG was found to be endogenously expressed by T-cells (Dixit et al., 2004) and its synthesis by T-cells declined with age, whereas the infusion of AG reversed the age-correlating increase in a large number of pro-inflammatory cytokines and chemokines in old rats (Dixit et al., 2009). This suggests that the loss of GHS-R1α/AG-signaling in immune cells contributes to the process of immune-senescence during aging, also known as “inflamm-aging.” Briefly, the process of inflamm-aging describes the gradual manifestation of an asymptomatic, chronic, systemic and low-grade inflammatory phenotype in the entire physiological system with age that contributes to development of aging-related diseases, such as insulin resistance, T2DM, AD, and PD (Xia et al., 2016).

Besides managing the inflammatory state, it was shown that AG dose-dependently inhibited the differentiation of isolated lymphocytes into the pro-inflammatory T helper cell (Th)17 subset, while GHS-R1α knockout mice exhibited heightened splenic levels of Th17 cells. It was confirmed that T-cell differentiation is coupled to the induction of the mTor/S6K1 and mTor/signal transducer and activator of transcription (STAT)3 pathways and AG blocked Th17 differentiation by inhibiting mTor activity (Xu Y. H. et al., 2015). This proposes that, as observed in neurons, AG drives the mTor-inactivating AMPK/TSC_1/2_ pathway in T-cells (Bayliss and Andrews, 2013; Peixoto et al., 2017). In agreement, AG elicits AMPK-evoked autophagy in the lymphoblastic Jurkat and Molt-4 cell lines (Heshmati et al., 2020), also considering that autophagy is initiated by the inactivation of mTOR (Lan et al., 2017). Generally, the differentiation toward the major, pro-inflammatory Th1 and Th17 subpopulations is dependent on mTORC1/STAT3 signaling as well as the presence of the cytokines IL-6 and transforming growth factor beta (Th17) and mTORC1/STAT4 plus IL-12 (Th1) (Saleiro and Platanias, 2015). As such, AG induces an anti-inflammatory phenotype in lymphocytes by downregulating the production of pro-inflammatory cytokines and, presumably in an AMPK/TSC_1/2_-mediated manner, limiting mTor-driven differentiation and proliferation.

To investigate the impact on T-cell instructed immune infiltration into the brain, studies in EAE models serve well in the assessment of AG's immunosuppressive capabilities. In the EAE mouse model, AG, but not DAG, improved the overall disease score, reduced lesion size, demyelination, microgliosis, inflammasome induction as well as iNOS and NF-κB activity in the spinal cord and downregulated the production of IL-1ß, IL-6, TNF-α and cyclooxygenase-2 (COX-2) by microglia and spinal cord-invading T-cells. Immune cell invasion into the spinal cord was only blocked by AG in some of these studies, however, which might have been related to the choice of the EAE-initiating antigen used for immunization (Theil et al., 2009; Souza-Moreira et al., 2013; Liu F. et al., 2019). Therefore, while highly implied, a T-cell suppressing function of AG needs to be confirmed in animal models of AD and PD.

#### Acylated Ghrelin Guards Against Blood Brain Barrier Damage by Reducing Inflammation

The BBB is a continuous, selective cell barrier in cerebral microvessels that separates the periphery (the circulating blood) from the brain. The vascular BBB is composed of an initial layer of endothelial cells, which seal off the paracellular gaps through the expression of tight junction proteins, and is further strengthened by pericytes and astrocyte end-feet. The breaching of the BBB/blood vessels, the leakage of peripheral material into the brain, fluid influx (edema), ion disbalance, the trespassing of peripheral immune cells as well as interrupted cerebral blood flow, are not only a concern in response to mechanical CNS injury, but are also major pathological features of AD and PD (Sweeney et al., 2018).

Importantly, inflammation augments the cerebral entry of peripheral immune cells by provoking the disruption of the BBB. For example, the genetic deletion of TNF-α and the utility of the microglial inhibitor minocycline attenuated the MPTP-induced permeabilization of the BBB in this PD animal model (Zhao et al., 2007). Furthermore, the cerebral infusion of LPS, in a mostly MMP-3-conyeyed manner, injured the BBB through the upregulation of MMP-3, MMP9, and the MMP-driven degradation of various tight junction proteins in rodents (Gurney et al., 2006). MMP-3 is also implicated in BBB damage in PD (Chung et al., 2013) and activates MMP-9 (Lee et al., 2014a), the MMP family member that directly proteolyzes BBB components (Lakhan et al., 2013).

AG not only quenches cerebral and systemic inflammation, as exemplified in the previous pages, but also protected endothelial cells of the BBB and neurons from apoptosis in various contexts, leading to the reduced release of the microglial inflammatory activator MMP-3 (Kim et al., 2005; Moon et al., 2009a; Lee J. et al., 2010; Lee et al., 2015). During various forms of CNS injury, AG was further shown to suppress systemic inflammation by stimulating anti-inflammatory signaling across the vagus nerve (Cheyuo et al., 2011), which led to reduced BBB damage and permeabilization, the transcriptional maintenance of the BBB tight junction proteins occludin and zonula occludens by vascular endothelial cells, the decreased death of neurons and astrocytes as well as the reduced spillage of DAMPs by apoptotic cells, such as neuron-specific enolase and S100β, into the blood stream. Thus, by diminishing total inflammation, the secretion of pro-inflammatory DAMPs and inflammation-driven BBB injury, AG was capable of preventing neutrophil infiltration into the CNS (Ersahin et al., 2010, 2011; Lopez et al., 2012a,b, Mohaddes et al., 2017).

## Acylated Ghrelin Induces the Release and Synthesis of Neuroprotective Insulin-Like Growth Factor 1

It must not be neglected that AG is involved in the expression and release of other powerful agents, such as the neuroprotective growth factor IGF-1 (reviewed in Costales and Kolevzon, 2016). In the periphery, AG has been deemed as the most powerful stimulator of the GH/IGF-1 axis (Nass et al., 2011). A clinical trial, although slightly underpowered, has indicated that the injection of the ghrelin analog MK-677 proved to sustain IGF-1 release in the long-term, which led to enhancements in the lean body mass of healthy, aged and non-obese subjects after a 1-year treatment period (Nass et al., 2008). Interestingly, AG equally appears to elevate the synthesis of IGF-1 in some brain regions. The injection of the ghrelin agonist GHRP-6 into healthy, adult rodents elevated the transcription levels of IGF-1 in the hypothalamus, the cerebellum and the hippocampus, but not the cortex (Frago et al., 2002). Furthermore, in IGF-1-positive brain areas, the increased phosphorylation of Akt, enhanced levels of the apoptosis-suppressing protein Bcl-2 as well as the inactivation of the apoptosis-mediator Bad were detected (Datta et al., 1997). This suggests that the AG-driven upregulation of IGF-1 in various brain areas occurs in the absence of any toxic insults, encouraging anti-apoptotic signaling in neurons. And indeed, the administration of GHRP-6 to old rats ameliorated the age-associated decline in IGF-1 levels in the cerebellum, inhibited caspase 9/3 and reduced cerebellar apoptosis (Paneda et al., 2003). The elevated hypothalamic synthesis of IGF-1 was also observed in AG or GHRP-6-injected obese rodents that were placed on a high fat dietary regiment (Garcia-Caceres et al., 2014).

## Insulin Resistance

### Early Cerebral Insulin Resistance Is Linked to Glucose Hypometabolism, Amyloid Pathology, and Cognitive Decline in Alzheimer's and Parkinson's Disease

While insulin is well-known for its metabolic role in the periphery, the insulin receptor is also widely expressed in the CNS. Indeed, insulin regulates various pivotal processes in neurons, such as the expression of glycolysis-associated enzymes and, thus, glucose metabolism, mitochondrial function and biogenesis, memory, gene and protein synthesis, cellular growth, functional autophagy, the protection from oxidative and ER stress and the induction of survival pathways. Given the pivotal and neuroprotective role of insulin-signaling in the brain and that T2DM is one of the greatest known risk factors for AD and PD, it is no surprise that its early desensitization in the CNS, believed to predominantly occur in response to chronic inflammation, promotes the development of AD and PD (see Holscher, 2019 for further information) (Blazquez et al., 2014; Werner and LeRoith, 2014; Holscher, 2020).

The negative effects of desensitized insulin on the brain are very apparent, especially in AD. For instance, a high-fat diet, which induces systemic insulin resistance, was shown to attenuate neuroprotective brain-derived neurotrophic factor levels, long-term potentiation (LTP) and dendritic spine density in the hippocampus of previously healthy mice, while accelerating Aβ plaque formation and memory loss in Aβ-transgenic animals (Ho et al., 2004; Stranahan et al., 2008). In addition, insulin suppresses the activity of GSK-3ß, a well-known Tau kinase (Lei et al., 2011), hence the loss of insulin signaling initiates Tau hyperphosphorylation and aggregation in AD (Hong and Lee, 1997; Schubert et al., 2003, 2004). Most importantly, insulin resistance results in pronounced glucose hypometabolism in the CNS. Studies in AD patients have confirmed the reduced sensitivity of the post-mortem-derived hippocampal and cortical brain tissue toward insulin. Moreover, the rate of inhibitory serine phosphorylation of IRS-1 (as a marker of insulin resistance), independent of even T2DM or the APOEε4 allele, rose gradually from previously healthy suspects to mild cognitive impairment (MCI) to AD patients, correlated with the quantity of Aß deposits and was inversely associated with episodic and working memory (Talbot et al., 2012). Such and related investigations led to the designation of AD as “type 3 diabetes” (Steen et al., 2005; Moloney et al., 2010). Furthermore, quantitative microarray RNA studies have revealed that AD patients, prior to the appearance of other neuropathological hallmarks, including Aß plaques and Tau neurofibrillary tangles, exhibit a decline in the cerebral expression of insulin-regulated genes that drive TCA and pyruvate metabolism (Zhao et al., 2015). In accord with insulin resistance, glucose hypometabolism in the CNS, for instance within the cortex, has been linked to the transition from MCI to AD and cognitive dysfunction (Lyingtunell et al., 1981; Hoyer et al., 1988; Ogawa et al., 1996; Drzezga et al., 2003; Mosconi et al., 2008). These early impediments in the neuronal insulin and glucose metabolism have been proposed to trigger a detrimental bioenergetic shift from glucose to alternative and less efficient energy substrates (reviewed in Neth and Craft, 2017) and have been suggested to precede any other pathological alteration, including even mitochondrial dysfunction, in AD patients (Zilberter and Zilberter, 2017; Holscher, 2019). This is further exacerbated by general reductions in the rate of blood flow and, therefore, cerebral glucose delivery in AD patients (Lyingtunell et al., 1981, Eberling et al., 1992; Ogawa et al., 1996; Roher et al., 2012; de Eulate et al., 2017). The latter may be another consequence of insulin resistance, since insulin promotes the cerebral blood flow by enhancing NO-driven vasoconstriction and endothelin 1-mediated capillary recruitment (Craft, 2009).

Impaired neuronal insulin signaling has also been identified in PD patients, with post-mortem analysis indicating that the prevalence of insulin receptors is reduced in the SNpc, the amygdala and the frontal white matter. Furthermore, components of the insulin pathway were found to be deactivated by inhibitory serine phosphorylation in the SN and basal ganglia, which appeared to precede dopamine neuron death, implying that the functional deterioration of insulin signaling manifests prematurely (Moroo et al., 1994; Takahashi et al., 1996; Tong et al., 2009; Morris et al., 2014). Interestingly, various studies have indicated that PD patients also exhibit cortical glucose hypometabolism and diminished blood flow in this area (Huang et al., 2008; Hosokai et al., 2009; Liepelt et al., 2009; Borghammer et al., 2010; Berti et al., 2012), a decreased rate of glucose consumption in the frontal lobe and caudate putamen (Xu Y. Q. et al., 2015) as well as the reduced expression of glycolytic enzymes in the putamen and the cerebellum (Dunn et al., 2014). Indeed, the bioenergetic alterations, in particular when present in the cortex, were associated with cognitive decline in PD patients, thus posing a potential predictor for the onset of PD-related dementia (Huang et al., 2008; Hosokai et al., 2009; Liepelt et al., 2009). In resemblance to AD, it has been postulated that these impairments in the cerebral turnover of glucose manifest prior to the appearance of other pathologic changes, for example the formation of Lewy bodies, in the PD brain (Zilberter and Zilberter, 2017).

### Acylated Ghrelin Prevents the Pathology-Associated Development of Insulin Resistance

Intriguingly, AG appears to preserve the cerebral insulin sensitivity. For example, The Aβ_25−35_-induced mouse model of AD displayed pathologic weight loss, decreased energy expenditure, deregulated insulin secretion and elevated HOMA-IR scores, which signified the presence of peripheral insulin resistance, whereas centrally administered AG restored these metabolic alterations. In the brains of these AD-like mice, AG further suppressed glycogen synthase kinase 3β (GSK-3β) activity and Tau hyperphosphorylation (Kang et al., 2015). GSK-3β is a Tau-phosphorylating kinase whose activity is aberrantly enhanced in response to the desensitization of insulin and the associated loss of Akt-signaling during diabetes mellitus and AD (Jolivalt et al., 2008; Zhang et al., 2018). Aβ, in turn, induces insulin resistance and GSK-3β by trapping insulin receptors in the neuronal cytoplasm and promoting inhibitory serine phosphorylation of insulin pathway components, for example IRS-1. As such, Aβ weakens insulin and Akt-signaling in the CNS (Zhao et al., 2008; Najem et al., 2016). In the MSG-generated rat model of obesity and cognitive decline, the administration of the ghrelin analog GHRP-6 normalized the plasma concentrations of various hormones, decreased the abnormally elevated hippocampal pools of Aβ and acetylcholine and enhanced the spatial memory of these rodents (Kutty and Subramanian, 2014). Also, in APPSwDI mice that were fed with a high glycemic index diet, AG, in fact, promoted weight loss, motor activity and spatial memory, while decreasing the degree of Ser^636^ phosphorylated IRS-1 in the mouse hippocampus, indicating that AG prevented the desensitization of insulin (Kunath et al., 2015). Lastly, the combinatorial application of AG and the insulin-re-sensitizing drug liraglutide, a glucagon-like peptide-1 (GLP-1) analog, was tested in the R6/2 Mouse Model of Huntington's disease (HD), resulting in the normalization of the chronically raised blood glucose levels as well as improved peripheral insulin sensitivity (HOMA-IR) and pancreatic ß-cell function (HOMA-ß) (Duarte et al., 2018). Notably, the co-injection of liraglutide and AG was more beneficial than liraglutide alone and prevented hyperinsulinemia in the cortex, forestalled the accumulation of pro-inflammatory triglycerides and cholesterol, increased IGF-1 levels, decreased lactate and AMP pools and doubled the cortical energy charge (Duarte et al., 2018). Therefore, the latter *in vivo* studies suggest that, via the elevated clearance of Aβ or related amyloids, AG fosters the cerebral insulin sensitivity and prevents insulin resistance-associated bioenergetic impairments, thus elevating cognition during AD.

Mechanistically, it is likely that AG prevents the desensitization of insulin through its potent anti-inflammatory properties (see also Figure 2). In the high-fat diet *in vivo* model of obesity, which shows low-grade systemic inflammation, AG counteracted the diet-driven rise in pro-inflammatory plasma free fatty acids and attenuated the amassment of triglycerides, the nuclear translocation of NF-κB and pro-inflammatory TNF-α production (Barazzoni et al., 2011, 2014). According to Barazzoni et al. and García-Cáceres et al., the administration of AG results in a phenotype that, despite exhibiting weight gain, displays low systemic inflammation and diminished triglyceride burden (Barazzoni et al., 2011; Garcia-Caceres et al., 2014). Additional studies indicated that AG, despite its orexigenic effects, blunted the amount of circulatory cytokines, such as IL-1β and IL-6, and oxidative stress markers in T1DM/T2DM animal models (Kyoraku et al., 2009; Garcia-Caceres et al., 2014). Indeed, AG acts as a potent immunosuppressor in the periphery and in the brain (as expounded in chapter 5 and shown in Figure 2). Given that inflammation is the driving factor in the maturation of insulin resistance during obesity, T2DM and neurodegenerative diseases, including AD and PD (Tateya et al., 2013; Holscher, 2019), it is implied that AG enhances insulin sensitivity by suppressing systemic inflammation, hyperlipidemia and oxidative assault.

### The Orexigenic Effects of Ghrelin May Encourage Secondary Metabolic Deregulation

Despite the latter promising studies, the long-term metabolic effects of AG are questionable. Generally, as concluded by a recent meta-analysis in diabetic patients suffering from gastropareses, the long-term clinical use of AG seems to be safe and is well-tolerated, even in metabolically susceptible populations (Hong et al., 2019). However, clinical studies in healthy, non-obese subjects have shown that the infusion of AG impairs the glucose-stimulated secretion of insulin, diminishes glucose tolerance and worsens insulin sensitivity (Gauna et al., 2004; Vestergaard et al., 2007; Tong et al., 2010, 2014). Furthermore, GHS-R1α antagonists, GOAT inhibitors and the genetic deletion of GHS-R1α enhanced the release of insulin, glucose tolerance, insulin sensitivity and weight loss in *in vivo* models of obesity (Sun et al., 2006; Esler et al., 2007; Longo et al., 2008; Barnett et al., 2010; Qi et al., 2011). Indeed, as an appetite-stimulating hormone, the long-term administration of a ghrelin analog promoted weight gain, which led to increased fasting blood glucose levels and deteriorated insulin sensitivity in healthy, aged adults (Nass et al., 2008). On the other hand, the injection of AG worsened glucose tolerance directly after administration, yet had rather beneficial metabolic long-term consequences, including weight loss, in T1DM, AD, and HD *in vivo* models (Granado et al., 2009; Kyoraku et al., 2009; Kunath et al., 2015; Duarte et al., 2018). Likewise, chronically increased plasma ghrelin levels did not lead to any complications in adult rodents, although it might promote hyperglycemia with age (Iwakura et al., 2005; Reed et al., 2008).

In conclusion, the injection of AG is safe, ameliorates the insulin resistance-driving inflammatory pathology and does not induce metabolic deregulation per se. Nonetheless, the orexigenic effects of the hormone, which encourage weight gain, may negatively affect the systemic insulin sensitivity and glucose tolerance in the long-term, favoring AD and PD.

## Dopamine

### Acylated Ghrelin Protects Nigrostriatal Dopaminergic Neurons and Boosts Dopamine Release in Parkinson's Disease

According to a previous analysis, ~30% of the dopamine-producing neurons in the SNpc are lost at the time at which clinical motor symptoms, including tremor, rigidity and bradykinesia, manifest in PD. Furthermore, neuronal death is accompanied by the independent destruction of axonal terminals in the SNpc and the degeneration of around 50–60% of neuronal projections from the SNpc toward other brain regions, in particular the striatum. Ultimately, these adverse alterations result in an estimated 50–70% reduction of total dopamine levels in the striatum/putamen (Cheng et al., 2010). Typically, symptomatic relief is provided through the supplementation of the lost striatal dopamine, yet these medications desensitize over time (Armstrong and Okun, 2020).

In the context of PD, there is evidence that AG not only supports the survival of SNpc-located neurons, but also boosts the availability of dopamine. As demonstrated *in vitro*, AG ameliorated the neuronal viability, cell death, caspase-3 activity and Bcl-2/Bax ratio, normalized the mitochondrial membrane potential, attenuated the production of ROS and malonaldehyde, stimulated the antioxidant enzymes MnSOD and catalase and inhibited the pro-inflammatory master transcription factor NF-κB in MPTP-stressed and GHS-R1α-expressing dopaminergic MES23.5 cells (Dong et al., 2009; Liu et al., 2010). Another study in the SN-derived SN4741 cell line, as confirmed with a GHS-R1α antagonist, showed that AG suppresses the LPS-provoked secretion of the pro-inflammatory cytokine IL-6 (Beynon et al., 2013). Similar to MPTP, AG was also capable of reversing the rotenone-induced blockade of mitochondrial complex 1 and prevented the toxin-induced drop in the mitochondrial membrane potential, resulting in the lessened leakage of the apoptosis-prompting cytochrome C, reduced caspase-3 activation and diminished cell death (Yu et al., 2016). *In vivo*, multiple independent studies in the MPTP rodent model of PD have shown that AG binds to GHS-R1α and activates SNpc dopaminergic neurons, rescues from neuronal death and prevents the depletion of dopamine in the striatum (Jiang et al., 2008; Andrews et al., 2009a; Moon et al., 2009a). Intriguingly, AG preserved the neuronal projections from the SNpc toward the striatum, indicating that AG has axoprotective capabilities (Moon et al., 2009a). Mechanistically, AG is responsible for the neuroprotective effects of caloric reduction and stimulates AMPK activity in the SN of MPTP-treated rodents (Bayliss et al., 2016). Furthermore, AG strengthens the neuronal resilience toward oxidative stress in an AMPK/UCP2-mediated manner, while improving the mitochondrial respiration, ATP production and the number of functional mitochondria through the induction of biogenesis (see chapter 3.2 for further insight). On the other hand, the deletion of ghrelin or GHS-R1α potentiated the neuronal loss and striatal dopamine deprivation in the MPTP mouse model (Andrews et al., 2009a). In addition, Andrews et al. showed that AG reversed the MPTP-provoked downregulation of TH in SNpc neurons *in vivo*. This effect was presumably a combination of the improved mitochondrial ATP generation and, thus, neuronal metabolism (Andrews et al., 2009a), the shielding from ROS that are generated through mitochondrial intoxication with MPTP (Andrews et al., 2009a), the reduction of microglial activation, the decreased release of pro-inflammatory cytokines, such as TNF-α and IL-1β, the diminished formation of NO metabolites (Moon et al., 2009a) as well as the anti-apoptotic effects of AG, as evident by the normalization of the Bcl-2/Bax ratio and the inhibition of caspase-3 (Jiang et al., 2008). Moreover, the stimulation of mitophagy via AG may prevent the accumulation of defective mitochondria and the associated generation of ROS (Bayliss and Andrews, 2013). For an overview of the neuroprotective pathways of AG, please refer to Figure 1.

**Figure 3:**
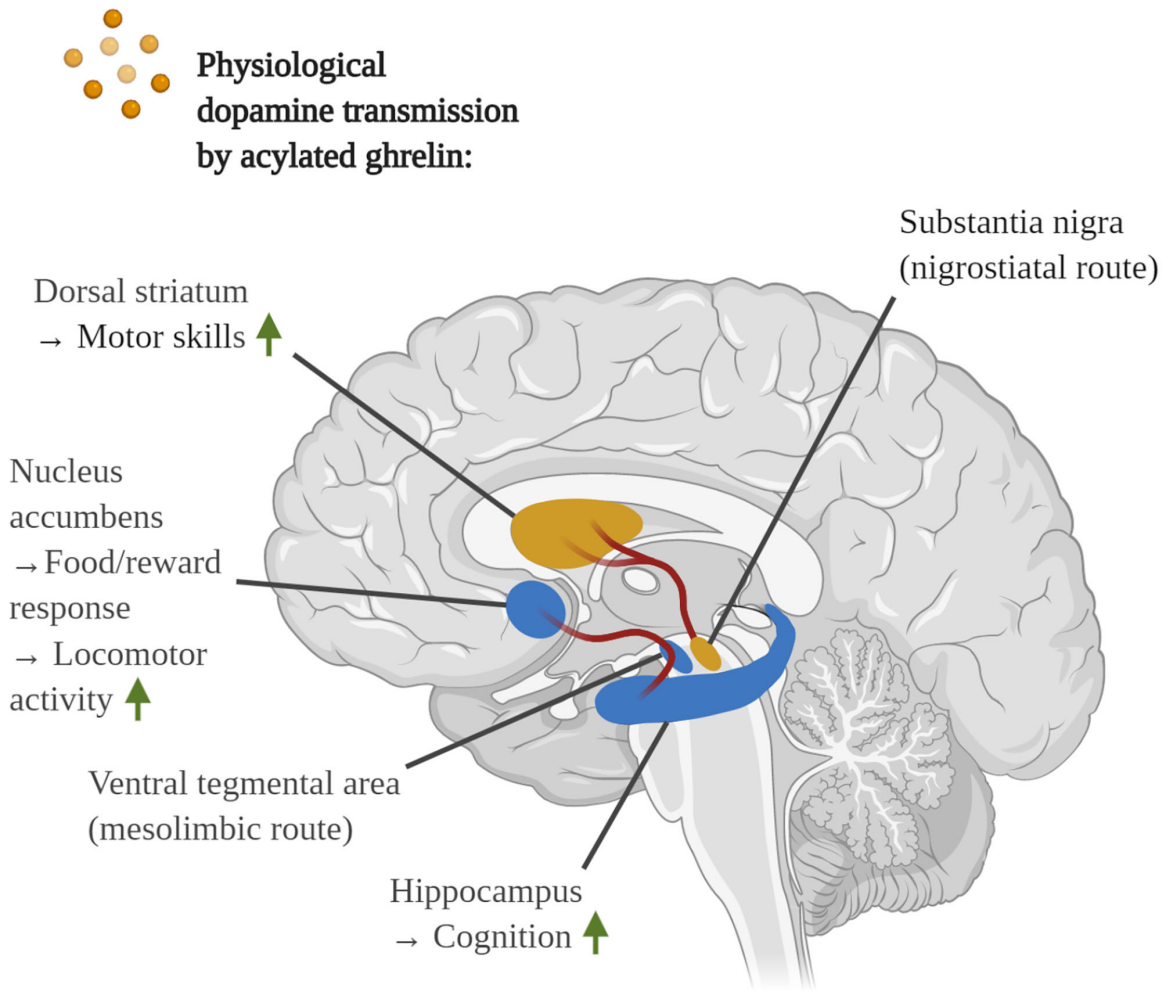
Depiction of the physiological dopamine transmission by AG from the SN to the dorsal striatum (nigrostriatal route) and the VTA to the nucleus accumbens and hippocampus (mesolimbic route). Notably, in addition to the loss of SN dopaminergic neurons and dopamine depletion in the dorsal striatum, PD patients exhibit neuronal degeneration in the VTA at a later stage, leading to bradyphrenia and dyskinesia. The mesocortical route of the VTA is not shown.

Besides safeguarding SNpc neurons, AG stimulates dopamine release in a physiological manner, as depicted in Figure 3. In an extensive study, it was demonstrated that AG binds to nigral neurons, triggers neuronal firing and enhances dopamine turnover, achieving an impressive three-fold increase in total dopamine levels in the striatum of healthy mice (Shi et al., 2013). The same group further discovered that the unilateral injection of AG into the SNpc ameliorated the cataleptic symptoms induced by the dopamine D2 receptor antagonist haloperidol, thus immediately improving the posture of the mice. The intra-SN injection of AG also improved the motor skills of haloperidol-treated DAT^SN^::DTA rodents in a recent study (Suda et al., 2018). In line with AG's role in dopamine metabolism, the injection of a GHS-R1α-antagonist into the SNpc was sufficient to cause motor disturbances and catalepsy in healthy rodents (Suda et al., 2018). Furthermore, the reduced expression of GHS-R1α was verified in induced pluripotent stem cells with a mutated or disrupted PARK2 gene, which is a mitochondrial gene implicated in mitochondrial quality control, mitophagy, and the development of early-onset PD (Pickrell and Youle, 2015), and in the SNpc of an *in vivo* model of PD (Suda et al., 2018). This implies that mitochondrial and bioenergetic deficits in dopaminergic neurons, which obviously impede the cellular gene transcription, culminate in the downregulation of GHS-R1α. Additionally, the co-emergence of insulin resistance, leading to reductions in PI3K/Akt/mTORC1-driven protein translation in dopaminergic neurons (Athauda and Foltynie, 2016; Anandhan et al., 2017; Holscher, 2019), presumably aggravates dopaminergic dysfunction and neuronal insensitivity toward AG.

As a side note, the combinatorial administration of the ghrelin agonist HM01 or AG with the dopamine-replacing drugs L-dopa or L-dopa/levodopa prevented the L-dopa- and 6-OHDA-associated slowing of the gastric motility and constipation in the 6-OHDA rodent model of PD (Wang et al., 2012; Karasawa et al., 2014). This resulted in markedly enhanced plasma levels of L-dopa and dopamine, indicating that ghrelin may improve the gastrointestinal absorption of dopamine replacement drugs (Wang et al., 2012). On the other hand, in a phase II clinical trial to investigate the constipation-mitigating effects of the ghrelin analog relamorelin, only 18 of the originally recruited 56 PD patients were able to finish the trial. In the vast majority of PD patients, relamorelin potentiated the frequency of incomplete bowel movements to a degree that the prolonged participation of the patients was impossible (Parkinson Study Group, 2017). As such, while AG can evidently improve gastrointestinal dysfunction (Hong et al., 2019), the occurrence of unwanted gastrointestinal side-effects in some PD patients may restrict the administration of ghrelin agonists in the long-term.

### Acylated Ghrelin Induces Dopamine Transmission in the Ventral Tegmental Area and Stimulates Locomotor Activity and Memory

Noteworthy, GHS-R1α was found to be expressed by ~60% of dopaminergic neurons in the VTA of rats (Abizaid et al., 2006). Collectively, animal experiments indicate that the injection of AG stimulates the mesolimbic transmission of dopamine from the VTA to the nucleus accumbens, thus heightening the release of dopamine in the nucleus accumbens and increasing food intake. When injected intracerebroventrically or intra-VTA, AG further enhanced the locomotor activity of rodents (see Figure 3) (Abizaid et al., 2006; Jerlhag et al., 2007, 2012; Quarta et al., 2009; Skibicka et al., 2011, 2012; Cornejo et al., 2018). Accordingly, knockout or pharmacological inhibition of GHS-R1α diminish mesolimbic dopamine transmission from the VTA to the nucleus accumbens, disheartening locomotion and the willingness of rodents to work for food rewards (Abizaid et al., 2006; Jerlhag et al., 2010, 2012; Skibicka et al., 2011, 2012). Given that dopamine deprivation in the VTA is linked to hypokinesia and bradyphrenia in PD patients (Yokochi, 2007), AG-associated improvements in this brain area might provide symptomatic relief. Moreover, even though the VTA predominantly projects dopamine toward the nucleus accumbens, it is also connected to the hippocampus (mesolimbic system) and the prefrontal cortex (mesocortical route) (Serrenho et al., 2019). Intriguingly, a study demonstrated that AG improved the object recognition memory of rats only when administered alone, but not when co-given with the D_1_R antagonist SKF 83566. Moreover, the injection of SKF 83566 itself did not negatively affect the behavioral performance of the rodents (Jacoby and Currie, 2011). Indeed, the presence of GHS-R1α and D_1_R heterodimers has been confirmed in the mouse hippocampus. While we will not further discuss this topic, cross-talk between ghrelin/GHS-R1α and dopamine/D_1_R-signaling was responsible for synaptic modifications as well as enhanced glutamate transmission, hippocampal plasticity and memory in rodents (Kern et al., 2015). Therefore, AG may elicit dopaminergic neurotransmission from the VTA to the hippocampus to improve cognition.

## Acylated Ghrelin Enhances Memory in Healthy Rodents and in AD Animal Models

AG also plays a major role in the retention of long-term memory. Various groups have reported that that the intracerebroventricular, intrahippocampal, or peripheral injection of AG or ghrelin agonists resulted in the binding of AG to GHS-R1α on hippocampal neurons, indicating that AG readily crosses the BBB. Furthermore, the administered AG increased the density of dendritic spines and synapses, enhanced LTP in the hippocampal CA1 region and led to improved learning and memory in healthy rodents (Carlini et al., 2002, 2004; Diano et al., 2006; Atcha et al., 2009). Impressively, the intracerebroventricular administration of AG restored the undernutrition-induced drop in the cognitive aptitude of rodents (Carlini et al., 2008), which is in line with ghrelin's physiological role as survival hormone (Mani and Zigman, 2017). On the other hand, the genetic deletion of ghrelin or knockdown of GHS-R1α lessened the numbers of hippocampal synapses, worsened LTP and impaired long-term memory (Diano et al., 2006; Davis et al., 2011), although discrepancies between spatial and contextual memory have been reported in GHS-R1α-null mice (Albarran-Zeckler et al., 2012). Albeit in the context of feeding, for a review of the physiological regulation of the hippocampal circuits via AG please see (Serrenho et al., 2019).

Additionally, by ameliorating the underlying cerebral pathology, AG and ghrelin agonists enhanced the cognitive performance of AD-like animals in multiple behavioral paradigms. In such AD *in vivo* models, AG rescued from hippocampal atrophy, synaptic damage and the degeneration of cholinergic projections, heightened brain glycogen levels, diminished Aß aggregation and deposition, blocked Aβ-imparted deficits in LTPs, normalized plasticity-associated p-CREB levels, improved insulin sensitivity, ameliorated microglial and astroglial immunoreactivity, augmented AMPK and suppressed cerebral GSK-3ß activity as well as Tau hyperphosphorylation (Moon et al., 2011; Dhurandhar et al., 2013; Kutty and Subramanian, 2014; Kang et al., 2015; Kunath et al., 2015; Ortega-Martinez, 2015; Bartolotti et al., 2016; Santos et al., 2017; Eslami et al., 2018; Jeong et al., 2018). In line with these findings, ghrelin knockout mice displayed deficits in spatial and recognition memory, worsened olfactory distinction and heightened micro- and astrogliosis in the rostral region of the hippocampus (Santos et al., 2017). The latter *in vivo* models of AD strongly imply that, at least in part, AG raises cognition by countering Aß and Tau toxicity. Direct cytoprotective properties were also observed in primary hippocampal and mHypoE-N42 hypothalamic neurons, in which AG opposed the Aß oligomer-induced cell death in a GHS-R1α-driven manner, diminished superoxide production, restored the neuronal Ca^2+^ homeostasis, rescued from mitochondrial membrane depolarization and reduced the activation of the Tau-kinase GSK-3ß (Martins et al., 2013; Gomes et al., 2014).

## Acylated Ghrelin Induces Neurogenesis in the Hippocampus

Various studies have testified that the administration of AG boosts adult hippocampal neurogenesis in healthy rodents (Zhao et al., 2014; Kent et al., 2015; Hornsby et al., 2016), dwarf rats (Li et al., 2013), in the 6-OHDA rodent model of PD, although neurogenesis was only enhanced in the non-lesioned brain hemisphere (Elabi et al., 2018), and the 5xFAD animal model of AD (Moon et al., 2014). Within the hippocampus, mice and dwarf rats were shown to possess Ki-67-positive, GHS-R1α-expressing immature neuroblasts in the granule cell layer of the DG (Moon et al., 2009b; Li et al., 2013; Hornsby et al., 2016). It was confirmed that, in an IGF-1-independent manner, AG stimulates the proliferation of neuroblasts, leading to an enlarged, doublecortin (DCX)-positive progenitor cell population in the DG of healthy mice and dwarf rats (Moon et al., 2009b; Li et al., 2013; Kent et al., 2015). There is also evidence that the injection of AG or overnight fasting raise the expression levels of the neurogenic transcription factor early growth response 1 (Hornsby et al., 2016). On the other hand, the antibody-mediated depletion of ghrelin decreased the DCX-expressing population of neuroblasts in the DG (Moon et al., 2009b). In concert, a study in depression-prone GHS-R1α^−/−^ mice revealed that the deletion of GHS-R1α exacerbated neuronal loss in response to chronic social defeat stress and diminished the proliferation of progenitor cells in the ventral DG (Walker et al., 2015). Interestingly, caloric restriction not only failed to demonstrate anti-depressive effects in these GHR-R1α knockout mice, but also provoked apoptosis within the neurogenic domain of the DG, as opposed to the growth-stimulating effects that were observed in control mice.

Notably, it has been postulated that GHS-R1α is, in fact, not present on immature neuroblasts and that AG possibly drives neurogenesis by stimulating the release of neurogenic factors by GHS-R1α-expressing adult dentate granule cells (Buntwal et al., 2019). Additional studies are needed to confirm these propositions in the hippocampus of healthy rodents and *in vivo* models of AD, however.

## Signs of Ghrelin Resistance During Obesity, Aging, and Alzheimer's Disease

Unfortunately, age- and disease-associated deteriorations in ghrelin-signaling have been implied. For instance, in elderly individuals, the plasma ghrelin levels, along with GH, were found to be decreased (Rigamonti et al., 2002). Similarly, obese individuals and patients with metabolic syndrome displayed reduced plasma ghrelin pools (Tschop et al., 2001; Rigamonti et al., 2002; Shiiya et al., 2002; Tesauro et al., 2005). While no relevant alterations in the blood levels of ghrelin have been observed in AD patients (Proto et al., 2006; Theodoropoulou et al., 2012), the basal plasma levels of ghrelin and the postprandial secretion of the hormone were diminished in PD patients (Fiszer et al., 2010; Unger et al., 2011). It has been proposed that the PD-related Lewy body pathology in the myenteric plexus of the stomach and in the DMV, which innervates the gut and navigates the gastrointestinal motility, might impair the release of ghrelin (Stoyanova, 2014). Strikingly, it was revealed that the locally synthesized levels of ghrelin, its recently discovered splicing analog ln2-ghrelin, GOAT and GHS-R1α were markedly downregulated in the temporal gyrus of AD patients. In contrast, the transcriptional levels of the GHS-R1α-trapping GHS-R1ß were significantly increased, indicating the desensitization of ghrelin in cognition-processing brain areas during AD (Gahete et al., 2010). Thus, the reduced secretion of ghrelin in PD as well as the emergence of cerebral ghrelin resistance in AD have been indicated.

Based on the latest evidence, obesity and T2DM seem to encourage the development of ghrelin resistance (Zigman et al., 2016). Interestingly, obese rodents not only display chronically lowered plasma levels of AG and total ghrelin as well as decreased gastric synthesis of ghrelin and GOAT, yet also fail to secrete the hormone post-prandially and do not respond to the appetite-stimulating effects of administered AG (Martin et al., 2004; Perreault et al., 2004; Briggs et al., 2010; Gardiner et al., 2010). Ghrelin resistance has also been postulated to blunt VTA and dopamine-regulated food/reward processing in obese rodents (Lockie et al., 2015). Indeed, diet-induced obesity has been shown to result in cerebral ghrelin resistance in rodents, which was marked by the attenuated expression of hypothalamic GHS-R1α, NPY and AgRP as well as the loss of Fos-immunoreactivity in ARC neurons in response to peripheral and central injections of AG (Briggs et al., 2010; Naznin et al., 2015). Additionally, it was unraveled that aged and overweight rodents display impaired translocation of plasma ghrelin across the BBB (Banks et al., 2008).

Since leptin functions as a physiological and anorexic counteragent to ghrelin in the hypothalamus, blocking intraneuronal AG/GHS-R1α-signaling, some studies have suggested that the elevated plasma pools of leptin during obesity weaken the sensitivity toward AG (Hewson et al., 2002; Kohno et al., 2007; Briggs et al., 2014). Thus, obesity and T2DM-associated hyperleptinemia (Maffei et al., 1995; Considine et al., 1996; Okumura et al., 2003; Pandey et al., 2015) may contribute to the desensitization of AG in the hypothalamus. It must be noted that GHS-R1α and leptin receptors strongly co-localize in >90% of neurons in the ARC of the hypothalamus, explaining the rapid neuronal desensitization in this region, whereas receptor co-synthesizing neurons are rarely found elsewhere in the CNS (Perello et al., 2012). Additionally, a high-fat diet, in an inflammation-mediated manner, promotes hypothalamic leptin resistance as well (El-Haschimi et al., 2000; Zhang et al., 2008). This implies that hyperleptinemia might initially attenuate cerebral ghrelin-signaling during obesity, yet is not sufficient to trigger chronic ghrelin resistance.

Concerningly, it has been indicated that inflammation might desensitize ghrelin signaling throughout the CNS. In this context, the systemic liberation of GH was shown to be impaired in obese mice (Briggs et al., 2010). Generally, somatotrophs manage the systemic release of GH, which is triggered by GHS-R1α-mediated mechanisms in the hypothalamus, the vagal afferent nerves and the anterior pituitary, hinting that these areas might desensitize to AG (Khatib et al., 2014). In particular, the vagal nodose ganglion has been shown to desensitize, exhibiting the reduced expression of GHS-R1α, diminished AMPK activation and reduced electric current flow upon exposure to AG (Naznin et al., 2015). Importantly, the exacerbated macrophage/microglial immunoreactivity and expression of TLR4, IL-6 and TNF-α were identified in the hypothalamus and vagal nerves of high-fat diet subjected mice, proposing a possible link between neuroinflammation and ghrelin resistance. In line with this theory, caloric restriction and the associated weight loss ameliorated these inflammatory changes, thus restoring the sensitivity toward AG (Naznin et al., 2018). As such, metabolic and cerebral inflammation possibly induce ghrelin resistance. On the other hand, it is plausible that the initial, inflammation-driven desensitization of insulin and other growth factors during obesity, T2DM, AD and PD (Maldonado-Ruiz et al., 2017; Holscher, 2019, 2020), which negatively affect the rate of cellular protein translation though the loss of Akt/mTORC1-signaling (Holscher, 2019; Liu and Sabatini, 2020), might be responsible for the reduced cerebral expression of GHS-R1α and GOAT in the cortex, hypothalamus and, potentially, further brain regions (Briggs et al., 2010; Gahete et al., 2010; Naznin et al., 2015).

Interestingly, a recent study demonstrated that klotho-deficient mice, which are *in vivo* models of accelerated aging, were unresponsive to the anorexigenic and life-extending effects of AG, indicating an age-associated development of ghrelin resistance. On the other hand, the ghrelin signaling potentiator rikkunshito, a herbal extract, was capable of enhancing the physiological function and rodent life-span (Fujitsuka et al., 2016). The decreased sensitivity toward AG could be related to an age-induced decline in the transcriptional levels of GHS-R1α, as reported for the anterior pituitary in 24-month-old Lou C/Jall rats, the vagal nerve in aged Fischer-344 rats and the brainstem in rats and dwarf rats (Katayama et al., 2000; Kappeler et al., 2004; Wu et al., 2009a). It was also verified that the age-associated decrease in the vagal expression of GHS-R1α potentiates LPS-triggered inflammation (Wu et al., 2009b). Considering the vast neuroprotective properties of AG, it is likely that the insufficient availability of ghrelin and the reduced ghrelin sensitivity in the CNS, such as the temporal lobe (Gahete et al., 2010), contribute to age-related cognitive decline.

## Conclusion

AG is a multi-talented hormone that has demonstrated great therapeutic potential. Synoptically, AG is neuroprotective, anti-oxidative, enhances the mitochondrial function, prevents mitochondrial hyperfission, induces autophagy and, possibly, mitophagy to dispose of amyloids and defective, ROS-generating mitochondria, suppresses systemic inflammatory responses and, possibly, the inflammasome, defies inflammation- and Aβ-triggered insulin resistance and the associated bioenergetic impairments, heightens the production of dopamine, promotes hippocampal neurogenesis and strengthens cognition in a direct and indirect manner in AD and PD. Notably, deacetylation half-life times (t_1/2_) of 4 h or 6.4 h, respectively, have been reported for AG in human plasma and ~27 min in rat serum, whereas the degradation t_1/2_ of total circulatory ghrelin has been estimated to be as short as ~9-11 min (De Vriese et al., 2004; Liu et al., 2008; Tong et al., 2013). Fortunately, synthetic ghrelin analogs offer prolonged stability, significant plasma release for up to 24 h, oral bioavailability and the ability to co-bind CD36, which may be useful to diminish Aβ-driven microglial inflammation (Bulgarelli et al., 2009; Muller et al., 2015; Berlanga-Acosta et al., 2017). To further enhance efficacy, the combination of ghrelin agonists with other growth factors, such as EGF (Barco et al., 2011, del Barco et al., 2011, del Barco-Herrera et al., 2013; Subiros et al., 2016), GH (Wu et al., 2009b; Zhou et al., 2017), insulin (Granado et al., 2011) and GLP-1 (Duarte et al., 2018), or DAG, to prevent AG-encouraged glucose intolerance and hypoinsulinemia (Gauna et al., 2004; Kiewiet et al., 2009), may be profitable. On the other hand, due to the possible development of ghrelin resistance in AD and reports of gastrointestinal complications in some PD patients, clinical studies are warranted to monitor the long-term effectiveness of AG. Considering the many intertwined pathologic processes in AD and PD, the varying clinical profile as well as the many historical failures of monotherapies, especially for Aβ-based therapies in AD, multi-targeted therapies, such as the application of the powerful hormone AG and related growth factors, deserve higher recognition. “Perhaps there is a field of treasures right there, waiting to be discovered” (Gault and Holscher, 2018).

## Contribution to the Field

Over the last decades, strategies to reduce the cerebral load of harmful monomeric, oligomeric, or insoluble amyloid deposits, such as Amyloid Beta, have repeatedly failed to produce any cognitive or motor improvements in patients of Alzheimer's (AD) and Parkinson's disease (PD). The many failures of these amyloid monotherapies indicate that novel therapeutic strategies are necessary. In addition, the multi-factorial pathology of AD and PD, ranging far beyond amyloid toxicity, suggests that targeting multiple pathologic factors might be more a more promising strategy to achieve clinical success. Interestingly, ghrelin, a peptide hormone that is released during fasting, has been shown to activate an impressive range of neuroprotective pathways that have the potential to ameliorate the majority of these pathologic alterations in AD and PD. Therefore, the review compiles the existing evidence, integrates information from other disease models to illustrate less discussed pathologic matters in AD and PD, outlines the neuroprotective functions of ghrelin and describes the underlying molecular mechanisms in great detail. Additionally, the manuscript evaluates the often disregarded clinical challenges, adverse effects and limitations of a possible pharmacological intervention with ghrelin analogs in AD and PD patients. Given the manifold promising and neuroprotective effects of ghrelin in the brain, but also to monitor the possible loss of effectiveness and the frequency and severity of undesirable side-effects, long-term clinical studies in AD and PD patients are warranted.

## Author Contributions

The manuscript was written and the figures were made by NR. CH reviewed and revised the manuscript. All authors contributed to the article and approved the submitted version.

## Conflict of Interest

The authors declare that the research was conducted in the absence of any commercial or financial relationships that could be construed as a potential conflict of interest.

## References

[B1] Abdalla M. M. I. (2015). Ghrelin – physiological functions and regulation. Eur. Endocrinol. 11, 90–95. 10.17925/EE.2015.11.02.90 29632576 PMC5819073

[B2] Abizaid A. Liu Z. W. Andrews Z. B. Shanabrough M. Borok E. Elsworth J. D.. (2006). Ghrelin modulates the activity and synaptic input organization of midbrain dopamine neurons while promoting appetite. J. Clin. Invest. 116, 3229–3239. 10.1172/JCI29867 17060947 PMC1618869

[B3] Adams B. (2020). Roche, AC Immune's Tau-Blocking Drug Flops in Alzheimer's as Biotech's Shares Halved. Available online at: https://www.fiercebiotech.com/biotech/roche-ac-immune-s-tau-blocking-drug-flops-alzheimer-s-as-biotech-s-shares-halved (accessed Octobar 01, 2020).

[B4] Afonina I. S. Zhong Z. Y. Karin M. Beyaert R. (2017). Limiting inflammation-the negative regulation of NF-kappa B and the NLRP3 inflammasome. Nat. Immunol. 18, 861–869. 10.1038/ni.3772 28722711

[B5] Albarran-Zeckler R. G. Brantley A. F. Smith R. G. (2012). Growth hormone secretagogue receptor (GHS-R1a) knockout mice exhibit improved spatial memory and deficits in contextual memory. Behav. Brain Res. 232, 13–19. 10.1016/j.bbr.2012.03.012 22484009 PMC3361606

[B6] Alvarez S. Munoz-Fernandez M. A. (2013). TNF-A may mediate inflammasome activation in the absence of bacterial infection in more than one way. PLoS ONE 8:e71477. 10.1371/journal.pone.0071477 23940760 PMC3737100

[B7] Alvarez-Erviti L. Rodriguez-Oroz M. C. Cooper J. M. Caballero C. Ferrer I. Obeso J. A.. (2010). Chaperone-mediated autophagy markers in parkinson disease brains. Arch. Neurol. 67, 1464–1472. 10.1001/archneurol.2010.198 20697033

[B8] Alves S. Churlaud G. Audrain M. Michaelsen-Preusse K. Fol R. Souchet B.. (2017). Interleukin-2 improves amyloid pathology, synaptic failure and memory in alzheimer's disease mice. Brain 140, 826–842. 10.1093/brain/aww330 28003243

[B9] Ambrosini Y. M. Borcherding D. Kanthasamy A. Kim H. J. Willette A. A. Jergens A.. (2019). The gut-brain axis in neurodegenerative diseases and relevance of the canine model: a review. Front. Aging Neurosci. 11:130. 10.3389/fnagi.2019.00130 31275138 PMC6591269

[B10] Amor S. Woodroofe M. N. (2014). Innate and adaptive immune responses in neurodegeneration and repair. Immunology 141, 287–291. 10.1111/imm.12134 23758741 PMC3930367

[B11] Anandhan A. Jacome M. S. Lei S. Hernandez-Franco P. Pappa A. Panayiotidis M. I.. (2017). Metabolic dysfunction in parkinson's disease: bioenergetics, redox homeostasis and central carbon metabolism. Brain Res. Bull. 133, 12–30. 10.1016/j.brainresbull.2017.03.009 28341600 PMC5555796

[B12] Anderson K. A. Ribar T. J. Lin F. M. Noeldner P. K. Green M. F. Muehlbauer M. J.. (2008). Hypothalamic CaMKK2 contributes to the regulation of energy balance. Cell Metab. 7, 377–388. 10.1016/j.cmet.2008.02.011 18460329

[B13] Anderson K. M. Olson K. E. Estes K. A. Flanagan K. Gendelman H. E. Mosley R. L. (2014). Dual destructive and protective roles of adaptive immunity in neurodegenerative disorders. Transl. Neurodegener. 3:25. 10.1186/2047-9158-3-25 25671101 PMC4323229

[B14] Andrews Z. B. Erion D. Beiler R. Liu Z. W. Abizaid A. Zigman J.. (2009a). Ghrelin promotes and protects nigrostriatal dopamine function via a UCP2-dependent mitochondrial mechanism. J. Neurosci. 29, 14057–14065. 10.1523/JNEUROSCI.3890-09.2009 19906954 PMC2845822

[B15] Andrews Z. B. Horvath B. Barnstable C. J. Elseworth J. Yang L. C. Beal M. F.. (2005). Uncoupling protein-2 is critical for nigral dopamine cell survival in a mouse model of parkinson's disease. J. Neurosci. 25, 184–191. 10.1523/JNEUROSCI.4269-04.2005 15634780 PMC6725213

[B16] Andrews Z. B. Liu Z. W. Walllingford N. Erion D. M. Borok E. Friedman J. M.. (2009b). UCP2 mediates ghrelin's action on NPY/AgRP neurons by lowering free radicals. Nature 459:736. 10.1038/nature08132 18668043 PMC4101536

[B17] Armstrong M. J. Okun M. S. (2020). Diagnosis and treatment of parkinson disease a review. JAMA 323, 548–560. 10.1001/jama.2019.22360 32044947

[B18] Arun S. Liu L. Donmez G. (2016). Mitochondrial biology and neurological diseases. Curr. Neuropharmacol. 14, 143–154. 10.2174/1570159X13666150703154541 26903445 PMC4825945

[B19] Atcha Z. Chen W. S. Ong A. B. Wong F. K. Neo A. Browne E. R.. (2009). Cognitive enhancing effects of ghrelin receptor agonists. Psychopharmacology 206, 415–427. 10.1007/s00213-009-1620-6 19652956

[B20] Athauda D. Foltynie T. (2016). Insulin resistance and parkinson's disease: a new target for disease modification? Progr. Neurobiol. 145, 98–120. 10.1016/j.pneurobio.2016.10.001 27713036

[B21] Avallone R. Demers A. Rodrigue-Way A. Bujold K. Harb D. Anghel S.. (2006). A growth hormone-releasing peptide that binds scavenger receptor CD36 and ghrelin receptor up-regulates sterol transporters and cholesterol efflux in macrophages through a peroxisome proliferator-activated receptor gamma- dependent pathway. Mol. Endocrinol. 20, 3165–3178. 10.1210/me.2006-0146 16959872

[B22] Baatar D. Patel K. Taub D. D. (2011). The effects of ghrelin on inflammation and the immune system. Mol. Cell. Endocrinol. 340, 44–58. 10.1016/j.mce.2011.04.019 21565248

[B23] Baek S. H. Park S. J. Jeong J. I. Kim S. H. Han J. Kyung J. W.. (2017). Inhibition of Drp1 ameliorates synaptic depression, a beta deposition, and cognitive impairment in an alzheimer's disease model. J. Neurosci. 37, 5099–5110. 10.1523/JNEUROSCI.2385-16.2017 28432138 PMC6596467

[B24] Balistreri C. R. Colonna-Romano G. Lio D. Candore G. Caruso C. (2009). TLR4 polymorphisms and ageing: implications for the pathophysiology of age-related diseases. J. Clin. Immunol. 29, 406–415. 10.1007/s10875-009-9297-5 19459036

[B25] Bamberger M. E. Harris M. E. McDonald D. R. Husemann J. Landreth G. E. (2003). A cell surface receptor complex for fibrillar beta-amyloid mediates microglial activation. J. Neurosci. 23, 2665–2674. 10.1523/JNEUROSCI.23-07-02665.2003 12684452 PMC6742111

[B26] Banks W. A. Burney B. O. Robinson S. M. (2008). Effects of triglycerides, obesity, and starvation on ghrelin transport across the blood-brain barrier. Peptides 29, 2061–2065. 10.1016/j.peptides.2008.07.001 18682266 PMC2586070

[B27] Banks W. A. Tschop M. Robinson S. M. Heiman M. L. (2002). Extent and direction of ghrelin transport across the blood-brain barrier is determined by its unique primary structure. J. Pharmacol. Exp. Ther. 302, 822–827. 10.1124/jpet.102.034827 12130749

[B28] Bansal V. Ryu S. Y. Blow C. Costantini T. Loomis W. Eliceiri B.. (2010). The hormone ghrelin prevents traumatic brain injury induced intestinal dysfunction. J. Neurotrauma 27, 2255–2260. 10.1089/neu.2010.1372 20858122 PMC3304249

[B29] Bansal V. Ryu S. Y. Lopez N. Allexan S. Krzyzaniak M. Eliceiri B.. (2012). Vagal stimulation modulates inflammation through a ghrelin mediated mechanism in traumatic brain injury. Inflammation 35, 214–220. 10.1007/s10753-011-9307-7 21360048 PMC3282000

[B30] Barazzoni R. Semolic A. Cattin M. R. Zanetti M. Guarnieri G. (2014). Acylated ghrelin limits fat accumulation and improves redox state and inflammation markers in the liver of high-fat-fed rats. Obesity 22, 170–177. 10.1002/oby.20454 23512916

[B31] Barazzoni R. Zanetti M. Semolic A. Cattin M. R. Pirulli A. Cattin L.. (2011). High-fat diet with acyl-ghrelin treatment leads to weight gain with low inflammation, high oxidative capacity and normal triglycerides in rat muscle. PLoS ONE 6:e26224. 10.1371/journal.pone.0026224 22039445 PMC3198460

[B32] Barcia C. Ros C. M. Annese V. Gomez A. Ros-Bernal F. Aguado-Yera D.. (2011). IFN-gamma signaling, with the synergistic contribution of TNF-alpha, mediates cell specific microglial and astroglial activation in experimental models of parkinson's disease. Cell Death Dis. 2:e142. 10.1038/cddis.2011.17 21472005 PMC3122054

[B33] Barco D. G. Montero E. Coro-Antich R. M. Brown E. Suarez-Alba J. Lopez L.. (2011). Coadministration of epidermal growth factor and growth hormone releasing peptide-6 improves clinical recovery in experimental autoimmune encephalitis. Restor. Neurol. Neurosci. 29, 243–252. 10.3233/RNN-2011-0595 21697595

[B34] Barnett B. P. Hwang Y. S. Taylor M. S. Kirchner H. Pfluger P. T. Bernard V.. (2010). Glucose and weight control in mice with a designed ghrelin O-acyltransferase inhibitor. Science 330, 1689–1692. 10.1126/science.1196154 21097901 PMC3068526

[B35] Bartolotti N. Segura L. Lazarov O. (2016). Diminished CRE-induced plasticity is linked to memory deficits in familial alzheimer's disease mice. J. Alzheimers Dis. 50, 477–489. 10.3233/JAD-150650 26682682 PMC4927858

[B36] Bayliss J. A. Andrews Z. B. (2013). Ghrelin is neuroprotective in parkinson's disease: molecular mechanisms of metabolic neuroprotection. Ther. Adv. Endocrinol. Metab. 4, 25–36. 10.1177/2042018813479645 23515333 PMC3593299

[B37] Bayliss J. A. Lemus M. B. Stark R. Santos V. V. Thompson A. Rees D. J.. (2016). Ghrelin-AMPK signaling mediates the neuroprotective effects of calorie restriction in parkinson's disease. J. Neurosci. 36, 3049–3063. 10.1523/JNEUROSCI.4373-15.2016 26961958 PMC4783502

[B38] Benner E. J. Banerjee R. Reynolds A. D. Sherman S. Pisarev V. M. Tsiperson V.. (2008). Nitrated alpha-synuclein immunity accelerates degeneration of nigral dopaminergic neurons. PLoS ONE 3:e1376. 10.1371/journal.pone.0001376 18167537 PMC2147051

[B39] Berlanga-Acosta J. Abreu-Cruz A. Herrera D. G. D. Mendoza-Mari Y. Rodriguez-Ulloa A. Garcia-Ojalvo A.. (2017). Synthetic growth hormone-releasing peptides (GHRPs): a historical appraisal of the evidences supporting their cytoprotective effects. Clin. Med. Insights Cardiol. 11:1179546817694558. 10.1177/1179546817694558 28469491 PMC5392015

[B40] Berthet A. Margolis E. B. Zhang J. Hsieh I. Zhang J. S. Hnasko T. S.. (2014). Loss of mitochondrial fission depletes axonal mitochondria in midbrain dopamine neurons. J. Neurosci. 34, 14304–14317. 10.1523/JNEUROSCI.0930-14.2014 25339743 PMC4205554

[B41] Berti V. C. Polito P. Borghammer S. Ramat L. Mosconi E. Vanzi M. T.. (2012). Alternative normalization methods demonstrate widespread cortical hypometabolism in untreated de novo Parkinson's disease. Q. J. Nuclear Med. Mol. Imag. 56, 299–308. Available online at: https://www.minervamedica.it/en/journals/nuclear-med-molecular-imaging/article.php?cod=R39Y2012N03A0299 22695340 PMC3846292

[B42] Beynon A. L. Brown M. R. Wright R. Rees M. I. Sheldon I. M. Davies J. S. (2013). Ghrelin inhibits LPS-induced release of IL-6 from mouse dopaminergic neurones. J. Neuroinflammation 10:40. 10.1186/1742-2094-10-40 23509933 PMC3614890

[B43] Bezzi P. Domercq M. Brambilla L. Galli R. Schols D. De Clercq E.. (2001). CXCR4-activated astrocyte glutamate release via TNFa: amplification by microglia triggers neurotoxicity. Nat. Neurosci. 4, 702–710. 10.1038/89490 11426226

[B44] Blazquez E. Velazquez E. Hurtado-Carneiro V. Ruiz-Albusac J. M. (2014). Insulin in the brain: its pathophysiological implications for states related with central insulin resistance, type 2 diabetes and alzheimer's disease. Front. Endocrinol. 5:161. 10.3389/fendo.2014.00161 25346723 PMC4191295

[B45] Boche D. Perry V. H. Nicoll J. A. R. (2013). Review: activation patterns of microglia and their identification in the human brain. Neuropathol. Appl. Neurobiol. 39, 3–18. 10.1111/nan.12011 23252647

[B46] Boland B. Kumar A. Lee S. Platt F. M. Wegiel J. Yu W. H.. (2008). Autophagy induction and autophagosome clearance in neurons: relationship to autophagic pathology in alzheimer's disease. J. Neurosci. 28, 6926–6937. 10.1523/JNEUROSCI.0800-08.2008 18596167 PMC2676733

[B47] Bonaz B. Sinniger V. Pellissier S. (2016). Anti-inflammatory properties of the vagus nerve: potential therapeutic implications of vagus nerve stimulation. J. Physiol. Lond. 594, 5781–5790. 10.1113/JP271539 27059884 PMC5063949

[B48] Borghammer P. Chakravarty M. Jonsdottir K. Y. Sato N. Matsuda H. Ito K.. (2010). Cortical hypometabolism and hypoperfusion in parkinson's disease is extensive: probably even at early disease stages. Brain Struct. Funct. 214, 303–317. 10.1007/s00429-010-0246-0 20361208

[B49] Bose A. Beal M. F. (2016). Mitochondrial dysfunction in parkinson's disease. J. Neurochem. 139, 216–231. 10.1111/jnc.13731 27546335

[B50] Briggs D. I. Enriori P. J. Lemus M. B. Cowley M. A. Andrews Z. B. (2010). Diet-induced obesity causes ghrelin resistance in arcuate NPY/AgRP neurons. Endocrinology 151, 4745–4755. 10.1210/en.2010-0556 20826561

[B51] Briggs D. I. Lockie S. H. Benzler J. L. sss Q. L. Stark R. Reichenbach A.. (2014). Evidence that diet-induced hyperleptinemia, but not hypothalamic gliosis, causes ghrelin resistance in NPY/AgRP neurons of male mice. Endocrinology 155, 2411–2422. 10.1210/en.2013-1861 24742194

[B52] Brochard V. Combadiere B. Prigent A. Laouar Y. Perrin A. Beray-Berthat V.. (2009). Infiltration of CD4^+^ lymphocytes into the brain contributes to neurodegeneration in a mouse model of parkinson disease. J. Clin. Invest. 119, 182–192. 10.1172/JCI36470 19104149 PMC2613467

[B53] Browne T. C. McQuillan K. McManus R. M. O'Reilly J. A. Mills K. H. Lynch M. A. (2013). IFN-gamma production by amyloid beta-specific Th1 cells promotes microglial activation and increases plaque burden in a mouse model of alzheimer's disease. J. Immunol. 190, 2241–2251. 10.4049/jimmunol.1200947 23365075

[B54] Bulgarelli I. Tamiazzo L. Bresciani E. Rapetti D. Caporali S. Lattuada D.. (2009). Desacyl-ghrelin and synthetic GH-secretagogues modulate the production of inflammatory cytokines in mouse microglia cells stimulated by beta-amyloid fibrils. J. Neurosci. Res. 87, 2718–2727. 10.1002/jnr.22088 19382238

[B55] Buntwal L. Sassi M. Morgan A. H. Andrews Z. B. Davies J. S. (2019). Ghrelin-mediated hippocampal neurogenesis: implications for health and disease. Trends Endocrinol. Metab. 30, 844–859. 10.1016/j.tem.2019.07.001 31445747

[B56] Cannavino J. Brocca L. Sandri M. Grassi B. Bottinelli R. Pellegrino M. A. (2015). The role of alterations in mitochondrial dynamics and PGC-1 alpha over-expression in fast muscle atrophy following hindlimb unloading. J. Physiol. Lond. 593, 1981–1995. 10.1113/jphysiol.2014.286740 25565653 PMC4405755

[B57] Canto C. Gerhart-Hines Z. Feige J. N. Lagouge M. Noriega L. Milne J. C.. (2009). AMPK regulates energy expenditure by modulating NAD^+^ metabolism and SIRT1 activity. Nature 458, 1056–1140. 10.1038/nature07813 19262508 PMC3616311

[B58] Carlini V. P. Martini A. C. Schioth H. B. Ruiz R. D. De Cuneom M. F. De Barioglio S. R. (2008). Decreased memory for novel object recognition in chronically food-restricted mice is reversed by acute ghrelin administration. Neuroscience 153, 929–934. 10.1016/j.neuroscience.2008.03.015 18434026

[B59] Carlini V. P. Monzon M. E. Varas M. M. Cragnolini A. B. Schioth H. B. Scimonelli T. N.. (2002). Ghrelin increases anxiety-like behavior and memory retention in rats. Biochem. Biophys. Res. Commun. 299, 739–743. 10.1016/S0006-291X(02)02740-7 12470640

[B60] Carlini V. P. Varas M. M. Cragnolini A. B. Schioth H. B. Scimonelli T. N. de Barioglio S. R. (2004). Differential role of the hippocampus, amygdala, and dorsal raphe nucleus in regulating feeding, memory, and anxiety-like behavioral responses to ghrelin. Biochem. Biophys. Res. Commun. 313, 635–641. 10.1016/j.bbrc.2003.11.150 14697239

[B61] Cataldo A. M. Hamilton D. J. Nixon R. A. (1994). Lysosomal abnormalities in degenerating neurons link neuronal compromise to senile plaque development in alzheimer-disease. Brain Res. 640, 68–80. 10.1016/0006-8993(94)91858-9 8004466

[B62] Cecarini V. Bonfili L. Cuccioloni M. Keller J. N. Bruce-Keller A. J. Eleuteri A. M. (2016). Effects of ghrelin on the proteolytic pathways of alzheimer's disease neuronal cells. Mol. Neurobiol. 53, 3168–3178. 10.1007/s12035-015-9227-x 26033219

[B63] Cenini G. Lloret A. Cascella R. (2019). Oxidative stress in neurodegenerative diseases: from a mitochondrial point of view. Oxid. Med. Cell. Longev. 2019:2105607. 10.1155/2019/2105607 31210837 PMC6532273

[B64] Ceranowicz P. Warzecha Z. Cieszkowski J. Ceranowicz D. Kusnierz-Cabala B. Bonior J.. (2017). Essential role of growth hormone and IGF-1 in therapeutic effect of ghrelin in the course of acetic acid-induced colitis. Int. J. Mol. Sci. 18:1118. 10.3390/ijms18061118 28538694 PMC5485942

[B65] Chang C. Su H. Zhang D. Wang Y. Shen Q. Liu B.. (2015). AMPK-dependent phosphorylation of GAPDH triggers sirt1 activation and is necessary for autophagy upon glucose starvation. Mol. Cell 60, 930–940. 10.1016/j.molcel.2015.10.037 26626483

[B66] Chao Y. X. He B. P. Tay S. S. W. (2009). Mesenchymal stem cell transplantation attenuates blood brain barrier damage and neuroinflammation and protects dopaminergic neurons against MPTP toxicity in the substantia nigra in a model of parkinson's disease. J. Neuroimmunol. 216, 39–50. 10.1016/j.jneuroim.2009.09.003 19819031

[B67] Chen C. Zhou M. Ge Y. C. Wang X. B. (2020). SIRT1 and aging related signaling pathways. Mech. Ageing Dev. 187:111215. 10.1016/j.mad.2020.111215 32084459

[B68] Chen W. W. Zhang X. Huang W. J. (2016). Role of neuroinflammation in neurodegenerative diseases. Mol. Med. Rep. 13, 3391–3396. 10.3892/mmr.2016.4948 26935478 PMC4805095

[B69] Cheng H. C. Ulane C. M. Burke R. E. (2010). Clinical progression in parkinson disease and the neurobiology of axons. Ann. Neurol. 67, 715–725. 10.1002/ana.21995 20517933 PMC2918373

[B70] Cheyuo C. Wu R. Q. Zhou M. A. Jacob A. Coppa G. Wang P. (2011). Ghrelin suppresses inflammation and neuronal nitric oxide synthase in focal cerebral ischemia via the vagus nerve. Shock 35, 258–265. 10.1097/SHK.0b013e3181f48a37 20720512

[B71] Cho D. H. Nakamura T. Fang J. G. Cieplak P. Godzik A. Gu Z.. (2009). S-Nitrosylation of Drp1 mediates beta-amyloid-related mitochondrial fission and neuronal injury. Science 324, 102–105. 10.1126/science.1171091 19342591 PMC2823371

[B72] Chu Y. P. Dodiya H. Aebischer P. Olanow C. W. Kordower J. H. (2009). Alterations in lysosomal and proteasomal markers in Parkinson's disease: relationship to alpha-synuclein inclusions. Neurobiol. Dis. 35, 385–398. 10.1016/j.nbd.2009.05.023 19505575

[B73] Chung H. Kim E. Lee D. H. Seo S. Ju S. Lee D.. (2007). Ghrelin inhibits apoptosis in hypothalamic neuronal cells during oxygen-glucose deprivation. Endocrinology 148, 148–159. 10.1210/en.2006-0991 17053024

[B74] Chung Y. C. Kim Y. S. Bok E. Yune T. Y. Maeng S. Jin B. K. (2013). MMP-3 contributes to nigrostriatal dopaminergic neuronal loss, BBB damage, and neuroinflammation in an MPTP mouse model of parkinson's disease. Mediators Inflamm. 2013:370526. 10.1155/2013/370526 23853428 PMC3703803

[B75] Cohen H. Y. Miller C. Bitterman K. J. Wall N. R. Hekking B. Kessler B.. (2004). Calorie restriction promotes mammalian cell survival by inducing the SIRT1 deacetylase. Science 305, 390–392. 10.1126/science.1099196 15205477

[B76] Considine R. V. Sinha M. K. Heiman M. L. Kriauciunas A. Stephens T. W. Nyce M. R.. (1996). Serum immunoreactive leptin concentrations in normal-weight and obese humans. N. Engl. J. Med. 334, 292–295. 10.1056/NEJM199602013340503 8532024

[B77] Conti B. Sugama S. Lucero J. Winsky-Sommerer R. Wirz S. A. Maher P.. (2005). Uncoupling protein 2 protects dopaminergic neurons from acute 1,2,3,6-methyl-phenyl-tetrahydropyridine toxicity. J. Neurochem. 93, 493–501. 10.1111/j.1471-4159.2005.03052.x 15816872

[B78] Coraci I. S. Husemann J. Berman J. W. Hulette C. Dufour J. H. Campanella G. K. (2002). CD36, a class B scavenger receptor, is expressed on microglia in alzheimer's disease brains and can mediate production of reactive oxygen species in response to beta-amyloid fibrils. Am. J. Pathol. 160, 101–112. 10.1016/S0002-9440(10)64354-4 11786404 PMC1867121

[B79] Cornejo M. P. Barrile F. De Francesco P. N. Portiansky E. L. Reynaldo M. Perello M. (2018). Ghrelin recruits specific subsets of dopamine and GABA neurons of different ventral tegmental area sub-nuclei. Neuroscience 392, 107–120. 10.1016/j.neuroscience.2018.09.027 30268780

[B80] Cortes N. Andrade V. Guzman-Martinez L. Estrella M. Maccioni R. B. (2018). Neuroimmune tau mechanisms: their role in the progression of neuronal degeneration. Int. J. Mol. Sci. 19:956. 10.3390/ijms19040956 29570615 PMC5979395

[B81] Costales J. Kolevzon A. (2016). The therapeutic potential of insulin-like growth factor-1 in central nervous system disorders. Neurosci. Biobehav. Rev. 63, 207–222. 10.1016/j.neubiorev.2016.01.001 26780584 PMC4790729

[B82] Cottrell D. A. Borthwick G. M. Johnson M. A. Ince P. G. Turnbull D. M. (2002). The role of cytochrome c oxidase deficient hippocampal neurones in alzheimer's disease. Neuropathol. Appl. Neurobiol. 28, 390–396. 10.1046/j.1365-2990.2002.00414.x 12366820

[B83] Craft S. (2009). The role of metabolic disorders in alzheimer disease and vascular dementia: two roads converged. Arch. Neurol. 66, 300–305. 10.1001/archneurol.2009.27 19273747 PMC2717716

[B84] Cummings D. E. Purnell J. Q. Frayo R. S. Schmidova K. Wisse B. E. Weigle D. S. (2001). A preprandial rise in plasma ghrelin levels suggests a role in meal initiation in humans. Diabetes 50, 1714–1719. 10.2337/diabetes.50.8.1714 11473029

[B85] Curry D. W. Stutz B. Andrews Z. B. Elsworth J. D. (2018). Targeting AMPK signaling as a neuroprotective strategy in parkinson's disease. J. Parkinsons Dis. 8, 161–181. 10.3233/JPD-171296 29614701 PMC6004921

[B86] Daitoku H. Yamagata K. Matsuzaki H. Hatta M. Fukamizu A. (2003). Regulation of PGC-1 promoter activity by protein kinase B and the forkhead transcription factor FKHR. Diabetes 52, 642–649. 10.2337/diabetes.52.3.642 12606503

[B87] Date Y. (2012). Ghrelin and the vagus nerve. Ghrelin 514, 261–269. 10.1016/B978-0-12-381272-8.00016-7 22975058

[B88] Date Y. Kojima M. Hosoda H. Sawaguchi A. Mondal M. S. Suganuma T.. (2000). Ghrelin, a novel growth hormone-releasing acylated peptide, is synthesized in a distinct endocrine cell type in the gastrointestinal tracts of rats and humans. Endocrinology 141, 4255–4261. 10.1210/endo.141.11.7757 11089560

[B89] Datta S. R. Dudek H. Tao X. Masters S. Fu H. A. Gotoh Y.. (1997). Akt phosphorylation of BAD couples survival signals to the cell-intrinsic death machinery. Cell 91, 231–241. 10.1016/S0092-8674(00)80405-5 9346240

[B90] Daulatzai M. A. (2012). Dysfunctional nucleus tractus solitarius: its crucial role in promoting neuropathogentic cascade of alzheimer's dementia-a novel hypothesis. Neurochem. Res. 37, 846–868. 10.1007/s11064-011-0680-2 22219130

[B91] Davis J. F. Choi D. L. Clegg D. J. Benoit S. C. (2011). Signaling through the ghrelin receptor modulates hippocampal function and meal anticipation in mice. Physiol. Behav. 103, 39–43. 10.1016/j.physbeh.2010.10.017 21036184 PMC3041863

[B92] de Eulate R. G. Goni I. Galiano A. Vidorreta M. Recio M. Riverol M.. (2017). Reduced cerebral blood flow in mild cognitive impairment assessed using phase-contrast MRI. J. Alzheimers Dis. 58, 585–595. 10.3233/JAD-161222 28453476

[B93] De Vriese C. Gregoire F. Lema-Kisoka R. Waelbroeck M. Robberecht P. Delporte C. (2004). Ghrelin degradation by serum and tissue homogenates: identification of the cleavage sites. Endocrinology 145, 4997–5005. 10.1210/en.2004-0569 15256494

[B94] Deboer M. D. (2011). Use of ghrelin as a treatment for inflammatory bowel disease: mechanistic considerations. Int. J. Pept. 2011:189242. 10.1155/2011/189242 21845198 PMC3154487

[B95] Dehay B. Bove J. Rodriguez-Muela N. Perier C. Recasens A. Boya P.. (2010). Pathogenic lysosomal depletion in parkinson's disease. J. Neurosci. 30, 12535–12544. 10.1523/JNEUROSCI.1920-10.2010 20844148 PMC6633458

[B96] del Barco D. G. Perez-Saad H. Rodriguez V. Marin J. Falcon V. Martin J.. (2011). Therapeutic effect of the combined use of growth hormone releasing peptide-6 and epidermal growth factor in an axonopathy model. Neurotox. Res. 19, 195–209. 10.1007/s12640-010-9160-8 20169434

[B97] del Barco-Herrera D. G. Martinez N. S. Coro-Antich M. Machado J. M. Alba J. S. Salgueiro S. R. (2013). Epidermal growth factor and growth hormone-releasing peptide-6: combined therapeutic approach in experimental stroke. Restor. Neurol. Neurosci. 31, 213–223. 10.3233/RNN-120262 23314006

[B98] Demers A. McNicoll N. Febbraio M. Servant M. Marleau S. Silverstein R.. (2004). Identification of the growth hormone-releasing peptide binding site in CD36: a photoaffinity cross-linking study. Biochem. J. 382, 417–424. 10.1042/BJ20040036 15176951 PMC1133797

[B99] Demetriades C. Plescher M. Teleman A. A. (2016). Lysosomal recruitment of TSC2 is a universal response to cellular stress. Nat. Commun. 7:10662. 10.1038/ncomms10662 26868506 PMC4754342

[B100] Depboylu C. Stricker S. Ghobril J. P. Oertel W. H. Priller J. Hoglinger G. U. (2012). Brain-resident microglia predominate over infiltrating myeloid cells in activation, phagocytosis and interaction with T-lymphocytes in the MPTP mouse model of parkinson disease. Exp. Neurol. 238, 183–191. 10.1016/j.expneurol.2012.08.020 22964486

[B101] Dhurandhar E. J. Allison D. B. van Groen T. Kadish I. (2013). Hunger in the absence of caloric restriction improves cognition and attenuates alzheimer's disease pathology in a mouse model. PLoS ONE 8:e60437. 10.1371/journal.pone.0060437 23565247 PMC3614512

[B102] Diano S. Farr S. A. Benoit S. C. McNay E. C. da Silva I. Horvath B.. (2006). Ghrelin controls hippocampal spine synapse density and memory performance. Nat. Neurosci. 9, 381–388. 10.1038/nn1656 16491079

[B103] Dixit V. D. Schaffer E. M. Pyle R. S. Collins G. D. Sakthivel S. K. Palaniappan R.. (2004). Ghrelin inhibits leptin- and activation-induced proinflammatory cytokine expression by human monocytes and T cells. J. Clin. Invest. 114, 57–66. 10.1172/JCI200421134 15232612 PMC437970

[B104] Dixit V. D. Yang H. W. Cooper-Jenkins A. Giri B. B. Patel K. Taub D. D. (2009). Reduction of T cell-derived ghrelin enhances proinflammatory cytokine expression: implications for age-associated increases in inflammation. Blood 113, 5202–5205. 10.1182/blood-2008-09-181255 19324904 PMC2686189

[B105] Donadelli M. Dando I. Fiorini C. Palmieri M. (2014). UCP2, a mitochondrial protein regulated at multiple levels. Cell. Mol. Life Sci. 71, 1171–1190. 10.1007/s00018-013-1407-0 23807210 PMC11114077

[B106] Dong J. J. Song N. Xie J. X. Jiang H. (2009). Ghrelin antagonized 1-methyl-4-phenylpyridinium (MPP+)-induced apoptosis in MES23.5 cells. J. Mol. Neurosci. 37, 182–189. 10.1007/s12031-008-9162-7 19052922

[B107] Drzezga A. Lautenschlager N. Siebner H. Riemenschneider M. Willoch F. Minoshima S.. (2003). Cerebral metabolic changes accompanying conversion of mild cognitive impairment into alzheimer's disease: a PET follow-up study. Eur. J. Nucl. Med. Mol. Imaging 30, 1104–1113. 10.1007/s00259-003-1194-1 12764551

[B108] Duarte A. I. Sjogren M. Santos M. S. Oliveira C. R. Moreira P. I. Bjorkqvist M. (2018). Dual therapy with liraglutide and ghrelin promotes brain and peripheral energy metabolism in the R6/2 mouse model of huntington's disease. Sci. Rep. 8:8961. 10.1038/s41598-018-27121-w 29895889 PMC5997749

[B109] Dunn L. Allen G. F. G. Mamais A. Ling H. L. Li A. Duberley K. E.. (2014). Dysregulation of glucose metabolism is an early event in sporadic parkinson's disease. Neurobiol. Aging 35, 1111–1115. 10.1016/j.neurobiolaging.2013.11.001 24300239 PMC3969149

[B110] Eberling J. L. Jagust W. J. Reed B. R. Baker M. G. (1992). Reduced temporal-lobe blood-flow in alzheimers-disease. Neurobiol. Aging 13, 483–491. 10.1016/0197-4580(92)90076-A 1508299

[B111] El Khoury J. B. Moore K. J. Means T. K. Leung J. Terada K. Toft M.. (2003). CD36 mediates the innate host response to beta-amyloid. J. Exp. Med. 197, 1657–1666. 10.1084/jem.20021546 12796468 PMC2193948

[B112] Elabi O. F. Duskova K. Davies J. S. Lane E. L. (2018). The impact of ghrelin on the survival and efficacy of dopaminergic fetal grafts in the 6-OHDA-lesioned rat. Neuroscience 395, 13–21. 10.1016/j.neuroscience.2018.10.045 30414880

[B113] El-Haschimi K. Pierroz D. D. Hileman S. M. Bjorbaek C. Flier J. S. (2000). Two defects contribute to hypothalamic leptin resistance in mice with diet-induced obesity. J. Clin. Invest. 105, 1827–1832. 10.1172/JCI9842 10862798 PMC378516

[B114] Ersahin M. Toklu H. Z. Erzik C. Akakin D. Tetik S. Sener G.. (2011). Ghrelin alleviates spinal cord injury in rats via its anti-inflammatory effects. Turk. Neurosurg. 21, 599–605. 10.5137/1019-5149.JTN.4736-11.0 22194122

[B115] Ersahin M. Toklu H. Z. Erzik C. Cetinel S. Akakin D. Velioglu-Ogunc A.. (2010). The anti-inflammatory and neuroprotective effects of ghrelin in subarachnoid hemorrhage-induced oxidative brain damage in rats. J. Neurotrauma 27, 1143–1155. 10.1089/neu.2009.1210 20205513

[B116] Eslami M. Sadeghi B. Goshadrou F. (2018). Chronic ghrelin administration restores hippocampal long-term potentiation and ameliorates memory impairment in rat model of alzheimer's disease. Hippocampus 28, 724–734. 10.1002/hipo.23002 30009391

[B117] Esler W. P. Rudolph J. Claus T. H. Tang W. F. Barucci N. Brown S. E.. (2007). Small-molecule ghrelin receptor antagonists improve glucose tolerance, suppress appetite, and promote weight loss. Endocrinology 148, 5175–5185. 10.1210/en.2007-0239 17656463

[B118] Ezquerro S. Mendez-Gimenez L. Becerril S. Moncada R. Valenti V. Catalan V.. (2016). Acylated and desacyl ghrelin are associated with hepatic lipogenesis, beta-oxidation and autophagy: role in NAFLD amelioration after sleeve gastrectomy in obese rats. Sci. Rep. 6:39942. 10.1038/srep39942 28008992 PMC5180230

[B119] Fang E. F. Hou Y. J. Palikaras K. Adriaanse B. A. Kerr J. S. Yang B. M.. (2019). Mitophagy inhibits amyloid-beta and tau pathology and reverses cognitive deficits in models of alzheimer's disease. Nat. Neurosci. 22, 401–412. 10.1038/s41593-018-0332-9 30742114 PMC6693625

[B120] Fang W. Y. Qu X. K. Yuan F. Wang W. G. Fei J.. (2013). AMPK activity is down-regulated in endothelial cells of GHS-R^−/−^ mice. Int. J. Clin. Exp. Pathol. 6, 1770–1780. Available online at: http://europepmc.org/article/PMC/3759483 24040441 PMC3759483

[B121] Farokhnia M. Portelli J. Lee M. R. McDiarmid G. R. Munjal V. Abshire K. M.. (2020). Effects of exogenous ghrelin administration and ghrelin receptor blockade, in combination with alcohol, on peripheral inflammatory markers in heavy-drinking individuals: Results from two human laboratory studies. Brain Res 1740:146851. 10.1016/j.brainres.2020.146851 32339499 PMC8715722

[B122] Ferens D. M. Yin L. Bron R. Hunne B. Ohashi-Doi K. Kitchener P. D.. (2010). Functional and *in situ* Hybridization Evidence That Preganglionic Sympathetic Vasoconstrictor Neurons express ghrelin receptors. Neuroscience 166, 671–679. 10.1016/j.neuroscience.2010.01.001 20060438

[B123] Ferreira-Marques M. Aveleira C. A. Carmo-Silva S. Botelho M. de Almeida L. P. Cavadas C. (2016). Caloric restriction stimulates autophagy in rat cortical neurons through neuropeptide Y and ghrelin receptors activation. Aging-Us 8, 1470–1484. 10.18632/aging.100996 27441412 PMC4993343

[B124] Ferrini F. Salio C. Lossi L. Merighi A. (2009). Ghrelin in central neurons. Curr. Neuropharmacol. 7, 37–49. 10.2174/157015909787602779 19721816 PMC2724662

[B125] Filichia E. Hoffer B. Qi X. Luo Y. (2016). Inhibition of Drp1 mitochondrial translocation provides neural protection in dopaminergic system in a parkinson's disease model induced by MPTP. Sci. Rep. 6:32656. 10.1038/srep32656 27619562 PMC5020318

[B126] Fisher Y. Nemirovsky A. Baron R. Monsonego A. (2011). Dendritic cells regulate amyloid-beta-specific T-cell entry into the brain: the role of perivascular amyloid-beta. J. Alzheimers. Dis. 27, 99–111. 10.3233/JAD-2011-102034 21765208

[B127] Fiszer U. Michalowska M. Baranowska B. Wolinska-Witort E. Jeske W. Jethon M.. (2010). Leptin and ghrelin concentrations and weight loss in parkinson's disease. Acta Neurol. Scand. 121, 230–236. 10.1111/j.1600-0404.2009.01185.x 20028343

[B128] Frago L. M. Baquedano E. Argente J. Chowen J. A. (2011). Neuroprotective actions of ghrelin and growth hormone secretagogues. Front. Mol. Neurosci. 4:23. 10.3389/fnmol.2011.00023 21994488 PMC3182030

[B129] Frago L. M. Paneda C. Dickson S. L. Hewson A. K. Argente J. Chowen J. A. (2002). Growth hormone (GH) and GH-releasing peptide-6 increase brain insulin-like growth factor-I expression and activate intracellular signaling pathways involved in neuroprotection. Endocrinology 143, 4113–4122. 10.1210/en.2002-220261 12239123

[B130] Frescas D. Valenti L. Accili D. (2005). Nuclear trapping of the forkhead transcription factor FoxO1 via sirt-dependent deacetylation promotes expression of glucogenetic genes. J. Biol. Chem. 280, 20589–20595. 10.1074/jbc.M412357200 15788402

[B131] Fuente-Martin E. Garcia-Caceres C. Argente-Arizon P. Diaz F. Granado M. Freire-Regatillo A.. (2016). Ghrelin regulates glucose and glutamate transporters in hypothalamic astrocytes. Sci. Rep. 6:23673. 10.1038/srep23673 27026049 PMC4812252

[B132] Fujikake N. Shin M. Shimizu S. (2018). Association between autophagy and neurodegenerative diseases. Front. Neurosci. 12:255. 10.3389/fnins.2018.00255 29872373 PMC5972210

[B133] Fujimura K. Wakino S. Minakuchi H. Hasegawa K. Hosoya K. Komatsu M.. (2014). Ghrelin protects against renal damages induced by angiotensin-II via an antioxidative stress mechanism in mice. PLoS ONE 9:e94373. 10.1371/journal.pone.0094373 24747517 PMC3991592

[B134] Fujitsuka N. Asakawa A. Morinaga A. Amitani M. S. Amitani H. Katsuura G.. (2016). Increased ghrelin signaling prolongs survival in mouse models of human aging through activation of sirtuin1. Mol. Psychiatry 21, 1613–1623. 10.1038/mp.2015.220 26830139 PMC5078860

[B135] Gagnon J. Anini Y. (2012). Insulin and norepinephrine regulate ghrelin secretion from a rat primary stomach cell culture. Endocrinology 153, 3646–3656. 10.1210/en.2012-1040 22691550

[B136] Gahete M. D. Rubio A. Cordoba-Chacon J. Gracia-Navarro F. Kineman R. D. Avila J.. (2010). Expression of the ghrelin and neurotensin systems is altered in the temporal lobe of alzheimer's disease patients. J. Alzheimers Dis. 22, 819–828. 10.3233/JAD-2010-100873 20858966

[B137] Gao S. Casals N. Keung W. Moran T. H. Lopaschuk G. D. (2013). Differential effects of central ghrelin on fatty acid metabolism in hypothalamic ventral medial and arcuate nuclei. Physiol. Behav. 118, 165–170. 10.1016/j.physbeh.2013.03.030 23680429

[B138] Garcia-Caceres C. Fuente-Martin E. Diaz F. Granado M. Argente-Arizon P. Frago L. M.. (2014). The opposing effects of ghrelin on hypothalamic and systemic inflammatory processes are modulated by its acylation status and food intake in male rats. Endocrinology 155, 2868–2880. 10.1210/en.2014-1074 24848869

[B139] Gardiner J. V. Campbell D. Patterson M. Kent A. Ghatei M. A. Bloom S. R.. (2010). The hyperphagic effect of ghrelin is inhibited in mice by a diet high in fat. Gastroenterology 138, 2468–2476. 10.1053/j.gastro.2010.02.012 20178795

[B140] Gault V. A. Holscher C. (2018). GLP-1 receptor agonists show neuroprotective effects in animal models of diabetes. Peptides 100, 101–107. 10.1016/j.peptides.2017.11.017 29412810

[B141] Gauna C. Meyler F. M. Janssen J. A. M. J. L. Delhanty P. J. D. Abribat T. Van Koetsveld P.. (2004). Administration of acylated ghrelin reduces insulin sensitivity, whereas the combination of acylated plus unacylated ghrelin strongly improves insulin sensitivity. J Clin. Endocrinol. Metab. 89, 5035–5042. 10.1210/jc.2004-0363 15472202

[B142] Gnanapavan S. Kola B. Bustin S. A. Morris D. G. McGee P. Fairclough P.. (2002). The tissue distribution of the mRNA of ghrelin and subtypes of its receptor, GHS-R, in humans. J. Clin. Endocrinol. Metab. 87, 2988–2991. 10.1210/jcem.87.6.8739 12050285

[B143] Goebel-Stengel M. Hofmann T. Elbelt U. Teuffel P. Ahnis A. Kobelt P.. (2013). The ghrelin activating enzyme ghrelin-O-acyltransferase (GOAT) is present in human plasma and expressed dependent on body mass index. Peptides 43, 13–19. 10.1016/j.peptides.2013.02.011 23454172

[B144] Gomes S. Martins I. Fonseca A. C. R. G. Oliveira C. R. Resende R. Pereira C. M. F. (2014). Protective effect of leptin and ghrelin against toxicity induced by amyloid- beta oligomers in a hypothalamic cell line. J. Neuroendocrinol. 26, 176–185. 10.1111/jne.12138 24528254

[B145] Gonzalez H. Elgueta D. Montoya A. Pacheco R. (2014). Neuroimmune regulation of microglial activity involved in neuroinflammation and neurodegenerative diseases. J. Neuroimmunol. 274, 1–13. 10.1016/j.jneuroim.2014.07.012 25091432

[B146] Gonzalez-Rey E. Chorny A. Delgado M. (2006). Therapeutic action of ghrelin in a mouse model of colitis. Gastroenterology 130, 1707–1720. 10.1053/j.gastro.2006.01.041 16697735

[B147] Granado M. Chowen J. A. Garcia-Caceres C. Delgado-Rubin A. Barrios V. Castillero E.. (2009). Ghrelin treatment protects lactotrophs from apoptosis in the pituitary of diabetic rats. Mol. Cell. Endocrinol. 309, 67–75. 10.1016/j.mce.2009.06.006 19540304

[B148] Granado M. Garcia-Caceres C. Tuda M. Frago L. M. Chowen J. A. Argente J. (2011). Insulin and growth hormone-releasing peptide-6 (GHRP-6) have differential beneficial effects on cell turnover in the pituitary, hypothalamus and cerebellum of streptozotocin (STZ)-induced diabetic rats. Mol. Cell. Endocrinol. 337, 101–113. 10.1016/j.mce.2011.02.002 21352888

[B149] Guan X. M. Yu H. Palyha O. C. McKee K. K. Feighner S. D. Sirinathsinghji D. J. S.. (1997). Distribution of mRNA encoding the growth hormone secretagogue receptor in brain and peripheral tissues. Mol. Brain Res. 48, 23–29. 10.1016/S0169-328X(97)00071-5 9379845

[B150] Guardia-Laguarta C. Area-Gomez E. Rub C. Liu Y. H. Magrane J. Becker D.. (2014). alpha-synuclein is localized to mitochondria-associated ER membranes. J. Neurosci. 34, 249–259. 10.1523/JNEUROSCI.2507-13.2014 24381286 PMC3866487

[B151] Gurney K. J. Estrada E. Y. Rosenberg G. A. (2006). Blood-brain barrier disruption by stromelysin-1 facilitates neutrophil infiltration in neuroinflammation. Neurobiol. Dis. 23, 87–96. 10.1016/j.nbd.2006.02.006 16624562

[B152] Hara T. Nakamura K. Matsui M. Yamamoto A. Nakahara Y. Suzuki-Migishima R.. (2006). Suppression of basal autophagy in neural cells causes neurodegenerative disease in mice. Nature 441, 885–889. 10.1038/nature04724 16625204

[B153] Hawley S. A. Pan D. A. Mustard K. J. Ross L. Bain J. Edelman A. M.. (2005). Calmodulin-dependent protein kinase kinase-beta is an alternative upstream kinase for AMP-activated protein kinase. Cell Metab. 2, 9–19. 10.1016/j.cmet.2005.05.009 16054095

[B154] Herzig S. Shaw R. J. (2018). AMPK: guardian of metabolism and mitochondrial homeostasis. Nat. Rev. Mol. Cell Biol. 19, 121–135. 10.1038/nrm.2017.95 28974774 PMC5780224

[B155] Heshmati M. Soltani A. Sanaei M. J. Nahid-Samiei M. Shirzad H. Jami M. S.. (2020). Ghrelin induces autophagy and CXCR4 expression via the SIRT1/AMPK axis in lymphoblastic leukemia cell lines. Cell. Signal. 66:109492. 10.1016/j.cellsig.2019.109492 31809874

[B156] Hewson A. K. Tung L. Y. C. Connell D. W. Tookman L. Dickson S. L. (2002). The rat arcuate nucleus integrates peripheral signals provided by leptin, insulin, and a ghrelin mimetic. Diabetes 51, 3412–3419. 10.2337/diabetes.51.12.3412 12453894

[B157] Ho L. Qin W. P. Pompl P. N. Xiang Z. M. Wang J. Zhao Z.. (2004). Diet-induced insulin resistance promotes amyloidosis in a transgenic mouse model of alzheimer's disease. Faseb J. 18, 902–924. 10.1096/fj.03-0978fje 15033922

[B158] Holscher C. (2019). Insulin signaling impairment in the brain as a risk factor in alzheimer's disease. Front. Aging Neurosci. 11:88. 10.3389/fnagi.2019.00088 31068799 PMC6491455

[B159] Holscher C. (2020). Brain insulin resistance: role in neurodegenerative disease and potential for targeting. Expert Opin. Investig. Drugs 29, 333–348. 10.1080/13543784.2020.1738383 32175781

[B160] Hong M. Lee V. M. Y. (1997). Insulin and insulin-like growth factor-1 regulate tau phosphorylation in cultured human neurons. J. Biol. Chem. 272, 19547–19553. 10.1074/jbc.272.31.19547 9235959

[B161] Hong S. W. Chun J. Kim J. Lee J. Lee H. J. Chung H.. (2019). Efficacy and safety of ghrelin agonists in patients with diabetic gastroparesis: a systematic review and meta-analysis. Gut Liver. 14, 589–600. 10.5009/gnl19103 31816671 PMC7492501

[B162] Hornsby A. K. E. Redhead Y. T. Rees D. J. Ratcliff M. S. G. Reichenbach A. Wells T.. (2016). Short-term calorie restriction enhances adult hippocampal neurogenesis and remote fear memory in a Ghsr-dependent manner. Psychoneuroendocrinology 63, 198–207. 10.1016/j.psyneuen.2015.09.023 26460782 PMC4686051

[B163] Horvath T. L. Andrews Z. B. Diano S. (2009). Fuel utilization by hypothalamic neurons: roles for ROS. Trends Endocrinol. Metab. 20, 78–87. 10.1016/j.tem.2008.10.003 19084428

[B164] Hosoda H. Kojima M. Matsuo H. Kangawa K. (2000). Ghrelin and des-acyl ghrelin: Two major forms of rat ghrelin peptide in gastrointestinal tissue. Biochem. Biophys. Res. Commun. 279, 909–913. 10.1006/bbrc.2000.4039 11162448

[B165] Hosokai Y. Nishio Y. Hirayama K. Takeda A. Ishioka T. Sawada Y.. (2009). Distinct patterns of regional cerebral glucose metabolism in parkinson's disease with and without mild cognitive impairment. Mov. Disord. 24, 854–862. 10.1002/mds.22444 19199357

[B166] Howard A. D. Feighner S. D. Cully D. F. Arena J. P. Liberator P. A. Rosenblum C. I.. (1996). A receptor in pituitary and hypothalamus that functions in growth hormone release. Science 273, 974–977. 10.1126/science.273.5277.974 8688086

[B167] Hoyer S. Oesterreich K. Wagner O. (1988). Glucose-metabolism as the site of the primary abnormality in early-onset dementia of alzheimer type. J. Neurol. 235, 143–148. 10.1007/BF00314304 3367161

[B168] Hsu C. P. Zhai P. Y. Yamamoto T. Maejima Y. Matsushima S. Hariharan N.. (2010). Silent information regulator 1 protects the heart from ischemia/reperfusion. Circulation 122, 2170–U193. 10.1161/CIRCULATIONAHA.110.958033 21060073 PMC3003297

[B169] Hu W. (2011). Parkinson's disease is a TH17 dominant autoimmune disorder against accumulated alpha-synuclein. Nat. Procedings 10.1038/npre.2011.6176.1. Available online at: https://www.nature.com/articles/npre.2011.6176.1#rightslink

[B170] Huang C. Mattis P. Perrine K. Brown N. Dhawan V. Eidelberg D. (2008). Metabolic abnormalities associated with mild cognitive impairment in parkinson disease. Neurology 70, 1470–1477. 10.1212/01.wnl.0000304050.05332.9c 18367705 PMC4454398

[B171] Huang J. Liu W. Doycheva D. M. Gamdzyk M. Lu W. T. Tang J. P.. (2019). Ghrelin attenuates oxidative stress and neuronal apoptosis via GHSR-1 alpha/AMPK/Sirt1/PGC-1 alpha/UCP2 pathway in a rat model of neonatal HIE. Free Radical Biol. Med. 141, 322–337. 10.1016/j.freeradbiomed.2019.07.001 31279091 PMC6718314

[B172] Huang J. Manning B. D. (2008). The TSC1-TSC2 complex: a molecular switchboard controlling cell growth. Biochem. J. 412, 179–190. 10.1042/BJ20080281 18466115 PMC2735030

[B173] Huang L. K. Chao S. P. Hu C. J. (2020). Clinical trials of new drugs for alzheimer disease. J. Biomed. Sci. 27:18. 10.1186/s12929-019-0609-7 31906949 PMC6943903

[B174] Iglesias M. A. Furler S. M. Cooney G. J. Kraegen E. W. Ye J. M. (2004). AMP-activated protein kinase activation by AICAR increases both muscle fatty acid and glucose uptake in white muscle of insulin-resistant rats *in vivo*. Diabetes 53, 1649–1654. 10.2337/diabetes.53.7.1649 15220186

[B175] Inoki K. Zhu T. Q. Guan K. L. (2003). TSC2 mediates cellular energy response to control cell growth and survival. Cell 115, 577–590. 10.1016/S0092-8674(03)00929-2 14651849

[B176] Iqbal K. Grundke-Iqbal I. (2010). Alzheimer's disease, a multifactorial disorder seeking multitherapies. Alzheimers Dementia 6, 420–424. 10.1016/j.jalz.2010.04.006 20813343 PMC2946155

[B177] Ising C. Venegas C. Zhang S. S. Scheiblich H. Schmidt S. V. Vieira-Saecker A.. (2019). NLRP3 inflammasome activation drives tau pathology. Nature 575, 669–673. 10.1038/s41586-019-1769-z 31748742 PMC7324015

[B178] Iwakura H. Hosoda K. Son C. Fujikura J. Tomita T. Noguchi M.. (2005). Analysis of rat insulin II promoter-ghrelin transgenic mice and rat glucagon promoter-ghrelin transgenic mice. J. Biol. Chem. 280, 15247–15256. 10.1074/jbc.M411358200 15701644

[B179] Jacoby S. M. Currie P. J. (2011). SKF 83566 attenuates the effects of ghrelin on performance in the object location memory task. Neurosci. Lett. 504, 316–320. 10.1016/j.neulet.2011.09.056 21982806

[B180] Jaeger P. A. Pickford F. Sun C. H. Lucin K. M. Masliah E. Wyss-Coray T. (2010). Regulation of amyloid precursor protein processing by the beclin 1 complex. PLoS ONE 5:e11103. 10.1371/journal.pone.0011102 20559548 PMC2886067

[B181] Jager S. Handschin C. Pierre J. Spiegelman B. M. (2007). AMP-activated protein kinase (AMPK) action in skeletal muscle via direct phosphorylation of PGC-1 alpha. Proc. Natl. Acad. Sci. U.S.A. 104, 12017–12022. 10.1073/pnas.0705070104 17609368 PMC1924552

[B182] Jeong Y. O. Shin S. J. Park J. Y. Ku B. K. Song J. S. Kim J. J.. (2018). MK-0677, a ghrelin agonist, alleviates amyloid beta-related pathology in 5XFAD mice, an animal model of alzheimer's disease. Int. J. Mol. Sci. 19:1800. 10.3390/ijms19061800 29912176 PMC6032329

[B183] Jerlhag E. Egecioglu E. Dickson S. L. Douhan A. Svensson L. Engel J. A. (2007). Ghrelin administration into tegmental areas stimulates locomotor activity and increases extracellular concentration of dopamine in the nucleus accumbens. Addict. Biol. 12, 6–16. 10.1111/j.1369-1600.2006.00041.x 17407492

[B184] Jerlhag E. Egecioglu E. Dickson S. L. Engel J. A. (2010). Ghrelin receptor antagonism attenuates cocaine- and amphetamine-induced locomotor stimulation, accumbal dopamine release, and conditioned place preference. Psychopharmacology 211, 415–422. 10.1007/s00213-010-1907-7 20559820 PMC2908453

[B185] Jerlhag E. Janson A. C. Waters S. Engel J. A. (2012). Concomitant release of ventral tegmental acetylcholine and accumbal dopamine by ghrelin in rats. PLoS ONE 7:e49557. 10.1371/journal.pone.0049557 23166710 PMC3498203

[B186] Jezek P. Holendova B. Garlid K. D. Jaburek M. (2018). Mitochondrial uncoupling proteins: subtle regulators of cellular redox signaling. Antioxid. Redox Signal. 29, 667–714. 10.1089/ars.2017.7225 29351723 PMC6071544

[B187] Jiang H. Betancourt L. Smith R. G. (2006). Ghrelin amplifies dopamine signaling by cross talk involving formation of growth hormone secretagogue receptor/dopamine receptor subtype 1 heterodimers. Mol. Endocrinol. 20, 1772–1785. 10.1210/me.2005-0084 16601073

[B188] Jiang H. Li L. J. Wang J. Xie J. X. (2008). Ghrelin antagonizes MPTP-induced neurotoxicity to the dopaminergic neurons in mouse substantia nigra. Exp. Neurol. 212, 532–537. 10.1016/j.expneurol.2008.05.006 18577498

[B189] Jiang H. S. Kang S. U. Zhang S. R. Karuppagounder S. Xu J. C. Lee Y. K.. (2016). Adult conditional knockout of PGC-1 alpha leads to loss of dopamine neurons. Eneuro 3:Eneuro.0183-16.2016. 10.1523/ENEURO.0183-16.2016 27622213 PMC5011687

[B190] Johnstone M. Gearing A. J. H. Miller K. M. (1999). A central role for astrocytes in the inflammatory response to beta-amyloid; chemokines, cytokines and reactive oxygen species are produced. J. Neuroimmunol. 93, 182–193. 10.1016/S0165-5728(98)00226-4 10378882

[B191] Jolivalt C. G. Lee C. A. Beiswenger K. K. Smith J. L. Orlov M. Torrance M. A.. (2008). Defective insulin signaling pathway and increased glycogen synthase kinase-3 activity in the brain of diabetic mice: parallels with alzheimer's disease and correction by insulin. J. Neurosci. Res. 86, 3265–3274. 10.1002/jnr.21787 18627032 PMC4937800

[B192] Kaleli H. N. Ozer E. Kaya V. O. Kutlu O. (2020). Protein kinase C isozymes and autophagy during neurodegenerative disease progression. Cells 9:553. 10.3390/cells9030553 32120776 PMC7140419

[B193] Kamp F. Exner N. Lutz A. K. Wender N. Hegermann J. Brunner B.. (2010). Inhibition of mitochondrial fusion by alpha-synuclein is rescued by PINK1, Parkin and DJ-1. Embo J. 29, 3571–3589. 10.1038/emboj.2010.223 20842103 PMC2964170

[B194] Kandan N. M. Pigino G. F. Brady S. T. Lazarov O. Binder L. I. Morfini G. A. (2013). Axonal degeneration in alzheimer's disease: when signaling abnormalities meet the axonal transport system. Exp. Neurol. 246, 44–53. 10.1016/j.expneurol.2012.06.003 22721767 PMC3465504

[B195] Kang D. Hamasaki N. (2005). Mitochondrial transcription factor A in the maintenance of mitochondrial DNA - Oeverview of its multiple roles. Ann. N. Y. Acad. Sci. 1042, 101–108. 10.1196/annals.1338.010 15965051

[B196] Kang S. Moon N. R. Kim D. S. Kim S. H. Park S. (2015). Central acylated ghrelin improves memory function and hippocampal AMPK activation and partly reverses the impairment of energy and glucose metabolism in rats infused with beta-amyloid. Peptides 71, 84–93. 10.1016/j.peptides.2015.07.005 26188171

[B197] Kappeler L. Zizzari P. Alliot J. Epelbaum J. Bluet-Pajot M. T. (2004). Delayed age-associated decrease in growth hormone pulsatile secretion and increased orexigenic peptide expression in the Lou C/Jall rat. Neuroendocrinology 80, 273–283. 10.1159/000083610 15677878

[B198] Karasawa H. Pietra C. Giuliano C. Garcia-Rubio S. Xu X. Yakabi S.. (2014). New ghrelin agonist, HM01 alleviates constipation and L-dopa-delayed gastric emptying in 6-hydroxydopamine rat model of parkinson's disease. Neurogastroenterol Motil. 26, 1771–1782. 10.1111/nmo.12459 25327342 PMC4457321

[B199] Katayama M. Nogami H. Nishiyama J. Kawase T. Kawamura K. (2000). Developmentally and regionally regulated expression of growth hormone secretagogue receptor mRNA in rat brain and pituitary gland. Neuroendocrinology 72, 333–340. 10.1159/000054602 11146416

[B200] Katugampola S. D. Pallikaros Z. Davenport A. P. (2001). [125I-His(9)]-ghrelin, a novel radioligand for localizing GHS orphan receptors in human and rat tissue: up-regulation of receptors with athersclerosis. Br. J. Pharmacol. 134, 143–149. 10.1038/sj.bjp.0704228 11522606 PMC1572927

[B201] Kenny R. Cai G. H. Bayliss J. A. Clarke M. Choo Y. L. Miller A. A.. (2013). Endogenous ghrelin's role in hippocampal neuroprotection after global cerebral ischemia: does endogenous ghrelin protect against global stroke? Am. J. Physiol. Regul. Integr. Comp. Physiol. 304, R980–R990. 10.1152/ajpregu.00594.2012 23576609

[B202] Kent B. A. Beynon A. L. Hornsby A. K. E. Bekinschtein P. Bussey T. J. Davies J. S.. (2015). The orexigenic hormone acyl-ghrelin increases adult hippocampal neurogenesis and enhances pattern separation. Psychoneuroendocrinology 51, 431–439. 10.1016/j.psyneuen.2014.10.015 25462915 PMC4275579

[B203] Kern A. Mavrikaki M. Ullrich C. Albarran-Zeckler R. Brantley A. F. Smith R. G. (2015). Hippocampal dopamine/DRD1 signaling dependent on the ghrelin receptor. Cell 163, 1176–1190. 10.1016/j.cell.2015.10.062 26590421 PMC4937825

[B204] Kerr J. S. Adriaanse B. A. Greig N. H. Mattson M. P. Cader M. Z. Bohr V. A.. (2017). Mitophagy and alzheimer's disease: cellular and molecular mechanisms. Trends Neurosci. 40, 151–166. 10.1016/j.tins.2017.01.002 28190529 PMC5341618

[B205] Khatib N. Gaidhane S. Gaidhane A. M. Khatib M. Simkhada P. Gode D.. (2014). Ghrelin: ghrelin as a regulatory peptide in growth hormone secretion. J. Clin. Diagn. Res. 8, MC13–7. 10.7860/JCDR/2014/9863.4767 25302229 PMC4190751

[B206] Khraiwesh H. Lopez-Dominguez J. A. Lopez-Lluch G. Navas P. de Cabo R. Ramsey J. J.. (2013). Alterations of ultrastructural and fission/fusion markers in hepatocyte mitochondria from mice following calorie restriction with different dietary fats. J. Gerontol. A Biol. Sci. Med. Sci. 68, 1023–1034. 10.1093/gerona/glt006 23403066 PMC3738026

[B207] Kiewiet R. M. van Aken M. O. K. van der Weerd U. P. Themmen A. P. N. Hofland L. J. de Rijke Y. B.. (2009). Effects of acute administration of acylated and unacylated ghrelin on glucose and insulin concentrations in morbidly obese subjects without overt diabetes. Eur. J. Endocrinol. 161, 567–573. 10.1530/EJE-09-0339 19628651

[B208] Kim C. Ho D. H. Suk J. E. You S. Michael S. Kang J.. (2013). Neuron-released oligomeric alpha-synuclein is an endogenous agonist of TLR2 for paracrine activation of microglia. Nat. Commun. 4:2534. 10.1038/ncomms2534 23463005 PMC4089961

[B209] Kim E. M. Hwang O. (2011). Role of matrix metalloproteinase-3 in neurodegeneration. J. Neurochem. 116, 22–32. 10.1111/j.1471-4159.2010.07082.x 21044079

[B210] Kim J. Kundu M. Viollet B. Guan K. L. (2011). AMPK and mTOR regulate autophagy through direct phosphorylation of Ulk1. Nat. Cell Biol. 13, 132–71. 10.1038/ncb2152 21258367 PMC3987946

[B211] Kim Y. S. Kim S. S. Cho J. J. Choi D. H. Hwang O. Shin D. H.. (2005). Matrix metalloproteinase-3: a novel signaling proteinase from apoptotic neuronal cells that activates microglia. J. Neurosci. 25, 3701–3711. 10.1523/JNEUROSCI.4346-04.2005 15814801 PMC6725382

[B212] Kisler K. Nelson A. R. Montagne A. Zlokovic B. V. (2017). Cerebral blood flow regulation and neurovascular dysfunction in alzheimer disease. Nat. Rev. Neurosci. 18, 419–434. 10.1038/nrn.2017.48 28515434 PMC5759779

[B213] Klatzmann D. Abbas A. K. (2015). The promise of low-dose interleukin-2 therapy for autoimmune and inflammatory diseases. Nat. Rev. Immunol. 15, 283–294. 10.1038/nri3823 25882245

[B214] Kleinz M. J. Maguire J. J. Skepper J. N. Davenport A. P. (2006). Functional and immunocytochemical evidence for a role of ghrelin and des-octanoyl ghrelin in the regulation of vascular tone in man. Cardiovasc. Res. 69, 227–235. 10.1016/j.cardiores.2005.09.001 16226234

[B215] Kodama T. Ashitani J. I. Matsumoto N. Kangawa K. Nakazato M. (2008). Ghrelin treatment suppresses neutrophil-dominant inflammation in airways of patients with chronic respiratory infection. Pulm. Pharmacol. Ther. 21, 774–779. 10.1016/j.pupt.2008.05.001 18571961

[B216] Kohno D. Gao H. Z. Muroya S. Kikuyama S. Yada T. (2003). Ghrelin directly interacts with neuropeptide-Y-containing neurons in the rat arcuate nucleus Ca2+ signalling via protein kinase A and N-type channel-dependent mechanisms and cross-talk with leptin and orexin. Diabetes 52, 948–956. 10.2337/diabetes.52.4.948 12663466

[B217] Kohno D. Nakata M. Maekawa F. Fujiwara K. Maejima Y. Kuramochi M.. (2007). Leptin suppresses ghrelin-induced activation of neuropeptide y neurons in the arcuate nucleus via phosphatidylinositol 3-kinase- and phosphodiesterase 3-mediated pathway. Endocrinology 148, 2251–2263. 10.1210/en.2006-1240 17303662

[B218] Komatsu M. Waguri S. Chiba T. Murata S. Iwata J. Tanida I.. (2006). Loss of autophagy in the central nervous system causes neurodegeneration in mice. Nature 441, 880–884. 10.1038/nature04723 16625205

[B219] Konturek P. C. Brzozowski T. Engel M. Burnat G. Gaca P. Kwiecien S.. (2009). Ghrelin ameliorates colonic inflammation. role of nitric oxide and sensory nerves. J. Physiol. Pharmacol. 60, 41–47. 19617644

[B220] Kortekaas R. Leenders K. L. van Oostrom J. C. H. Vaalburg W. Bart J. Willemsen A. T. M.. (2005). Blood-brain barrier dysfunction in parkinsonian midbrain *in vivo*. Ann. Neurol 57, 176–179. 10.1002/ana.20369 15668963

[B221] Kraft E. N. Cervone D. T. Dyck D. J. (2019). Ghrelin stimulates fatty acid oxidation and inhibits lipolysis in isolated muscle from male rats. Physiol. Rep. 7:e14028. 10.14814/phy2.14028 30963694 PMC6453820

[B222] Ku J. M. Andrews Z. B. Barsby T. Reichenbach A. Lemus M. B. Drummond G. R.. (2015). Ghrelin-related peptides exert protective effects in the cerebral circulation of male mice through a nonclassical ghrelin receptor(s). Endocrinology 156, 280–290. 10.1210/en.2014-1415 25322462 PMC4272401

[B223] Ku J. M. Taher M. Chin K. Y. Barsby T. Austin V. Wong C. H. Y.. (2016). Protective actions of des-acylated ghrelin on brain injury and blood-brain barrier disruption after stroke in mice. Clin. Sci. 130, 1545–1558. 10.1042/CS20160077 27303049

[B224] Kunath N. van Groen T. Allison D. B. Kumar A. Dozier-Sharpe M. Kadish I. (2015). Ghrelin agonist does not foster insulin resistance but improves cognition in an alzheimer's disease mouse model. Sci. Rep. 5:11452. 10.1038/srep11452 26090621 PMC4473679

[B225] Kurkowska-Jastrzebska I. Wronska A. Kohutnicka M. Czlonkowski A. Czlonkowska A. (1999). MHC class II positive microglia and lymphocytic infiltration are present in the substantia nigra and striatum in mouse model of parkinson's disease. Acta Neurobiol. Exp. 59, 1–8. 10230070 10.55782/ane-1999-1289

[B226] Kutty B. M. Subramanian S. (2014). Amyloid beta lowering and cognition enhancing effects of ghrelin receptor analog [D-Lys (3)] GHRP-6 in rat model of obesity. Biochem. Biophys. 51, 257–262. Available online at: https://www.semanticscholar.org/paper/Amyloid-beta-lowering-and-cognition-enhancing-of-in-Madhavadas-Kutty/709c2106abfeee0d1d6c8477b8ded7e630ad0997?p2df 25296496

[B227] Kyoraku I. Shiomi K. Kangawa K. Nakazato M. (2009). Ghrelin reverses experimental diabetic neuropathy in mice. Biochem. Biophys. Res. Commun. 389, 405–408. 10.1016/j.bbrc.2009.08.171 19733151

[B228] Lage R. Vazquez M. J. Varela L. Saha A. K. Vidal-Puig A. Nogueiras R.. (2010). Ghrelin effects on neuropeptides in the rat hypothalamus depend on fatty acid metabolism actions on BSX but not on gender. Faseb J. 24, 2670–2679. 10.1096/fj.09-150672 20335227 PMC3230529

[B229] Lagouge M. Argmann C. Gerhart-Hines Z. Meziane H. Lerin C. Daussin F. (2006). Resveratrol improves mitochondrial function and protects against metabolic disease by activating SIRT1 and PGC-1 alpha. Cell 127, 1109–1122. 10.1016/j.cell.2006.11.013 17112576

[B230] Lakhan S. E. Kirchgessner A. Tepper D. Leonard A. (2013). Matrix metalloproteinases and blood-brain barrier disruption in acute ischemic stroke. Front. Neurol. 4:32. 10.3389/fneur.2013.00032 23565108 PMC3615191

[B231] Lan A. P. Chen J. Zhao Y. L. Chai Z. F. Hu Y. (2017). mTOR signaling in parkinson's disease. Neuromol. Med. 19, 1–10. 10.1007/s12017-016-8417-7 27263112

[B232] Lan F. Cacicedo J. M. Ruderman N. Ido Y. (2008). SIRT1 modulation of the acetylation status, cytosolic localization, and activity of LKB1 - possible role in AMP-activated protein kinase activation. J. Biol. Chem. 283, 27628–27635. 10.1074/jbc.M805711200 18687677 PMC2562073

[B233] Langston J. W. Ballard P. Tetrud J. W. Irwin I. (1983). Chronic parkinsonism in humans due to a product of meperidine-analog synthesis. Science 219, 979–980. 10.1126/science.6823561 6823561

[B234] Larbi A. Pawelec G. Witkowski J. M. Schipper H. M. Derhovanessian E. Goldeck D.. (2009). Dramatic shifts in circulating CD4 but not CD8 T cell subsets in mild Alzheimer's disease. J. Alzheimers. Dis. 17, 91–103. 10.3233/JAD-2009-1015 19494434

[B235] Lee H. J. Suk J. E. Patrick C. Bae E. J. Cho J. H. Rho S.. (2010). Direct transfer of alpha-synuclein from neuron to astroglia causes inflammatory responses in synucleinopathies. J. Biol. Chem. 285, 9262–9272. 10.1074/jbc.M109.081125 20071342 PMC2838344

[B236] Lee J. Lim E. Kim Y. Li E. Park S. (2010). Ghrelin attenuates kainic acid-induced neuronal cell death in the mouse hippocampus. J. Endocrinol. 205, 262–269. 10.1677/JOE-10-0040 20351014

[B237] Lee J. Y. Choi H. Y. Ahn H. J. Ju B. G. Yune T. Y. (2014a). Matrix metalloproteinase-3 promotes early blood-spinal cord barrier disruption and hemorrhage and impairs long-term neurological recovery after spinal cord injury. Am. J. Pathol. 184, 2985–3000. 10.1016/j.ajpath.2014.07.016 25325922

[B238] Lee J. Y. Choi H. Y. Na W. H. Ju B. G. Yune T. Y. (2014b). Ghrelin inhibits BSCB disruption/hemorrhage by attenuating MMP-9 and SUR1/TrpM4 expression and activation after spinal cord injury. Biochim. Biophys. Acta Mol. Basis Dis. 1842, 2403–2412. 10.1016/j.bbadis.2014.09.006 25261791

[B239] Lee J. Y. Choi H. Y. Yune T. Y. (2015). MMP-3 secreted from endothelial cells of blood vessels after spinal cord injury activates microglia, leading to oligodendrocyte cell death. Neurobiol. Dis. 82, 141–151. 10.1016/j.nbd.2015.06.002 26079709

[B240] Lee J. Y. Chung H. Yoo Y. S. Oh Y. J. Oh T. H. Park S.. (2010). Inhibition of apoptotic cell death by ghrelin improves functional recovery after spinal cord injury. Endocrinology 151, 3815–3826. 10.1210/en.2009-1416 20444938

[B241] Lee J. Y. Oh T. H. Yune T. Y. (2011). Ghrelin inhibits hydrogen peroxide-induced apoptotic cell death of oligodendrocytes via ERK and p38MAPK signaling. Endocrinology 152, 2377–2386. 10.1210/en.2011-0090 21467197

[B242] Lee J. Y. Yune T. Y. (2014). Ghrelin inhibits oligodendrocyte cell death by attenuating microglial activation. Endocrinol. Metab. 29, 371–378. 10.3803/EnM.2014.29.3.371 25309797 PMC4192814

[B243] Lee S. Kim Y. Li E. Park S. (2012). Ghrelin protects spinal cord motoneurons against chronic glutamate excitotoxicity by inhibiting microglial activation. Korean J. Physiol. Pharmacol. 16, 43–48. 10.4196/kjpp.2012.16.1.43 22416219 PMC3298825

[B244] Lei P. Ayton S. Bush A. I. Adlard P. A. (2011). GSK-3 in neurodegenerative diseases. Int. J. Alzheimers. Dis. 2011:189246. 10.4061/2011/189246 21629738 PMC3100544

[B245] Letiembre M. Liu Y. Walter S. Hao W. L. Pfander T. Wrede A.. (2009). Screening of innate immune receptors in neurodegenerative diseases: a similar pattern. Neurobiol. Aging 30, 759–768. 10.1016/j.neurobiolaging.2007.08.018 17905482

[B246] Leung P. K. Chow K. B. S. Lau P. N. Chu K. M. Chan C. B. Cheng C. H. K.. (2007). The truncated ghrelin receptor polypeptide (GHS-R1b) acts as a dominant-negative mutant of the ghrelin receptor. Cell. Signal. 19, 1011–1022. 10.1016/j.cellsig.2006.11.011 17229547

[B247] Li C. Wu X. Liu S. Zhao Y. Zhu J. Liu K. (2019). Roles of neuropeptide y in neurodegenerative and neuroimmune diseases. Front. Neurosci. 13:869. 10.3389/fnins.2019.00869 31481869 PMC6710390

[B248] Li E. Kim Y. Kim S. Park S. (2013). Ghrelin-induced hippocampal neurogenesis and enhancement of cognitive function are mediated independently of GH/IGF-1 axis: lessons from the spontaneous dwarf rats. Endocr. J. 60, 1065–1075. 10.1507/endocrj.EJ13-0045 23774069

[B249] Li W. G. Gavrila D. Liu X. B. Wang L. X. Gunnlaugsson S. Stoll L. L.. (2004). Ghrelin inhibits proinflammatory responses and nuclear factor-kappa B activation in human endothelial cells. Circulation 109, 2221–2226. 10.1161/01.CIR.0000127956.43874.F2 15117840

[B250] Liao P. Z. Yang D. Liu D. Zheng Y. H. (2017). GLP-1 and ghrelin attenuate high glucose/high lipid-induced apoptosis and senescence of human microvascular endothelial cells. Cell. Physiol. Biochem. 44, 1842–1855. 10.1159/000485820 29224011

[B251] Liepelt I. Relmold M. Maetzler W. Godau J. Reischl G. Gaenslen A.. (2009). Cortical hypometabolism assessed by a metabolic ratio in parkinson's disease primarily reflects cognitive deterioration-[F-18]FDG-PET. Mov. Disord. 24, 1504–1511. 10.1002/mds.22662 19489069

[B252] Liu F. Li Z. J. He X. Yu H. Y. Feng J. (2019). Ghrelin attenuates neuroinflammation and demyelination in experimental autoimmune encephalomyelitis involving NLRP3 inflammasome signaling pathway and pyroptosis. Front. Pharmacol 10:1320. 10.3389/fphar.2019.01320 31780940 PMC6851267

[B253] Liu G. Y. Sabatini D. M. (2020). mTOR at the nexus of nutrition, growth, ageing and disease. Nat. Rev. Mol. Cell Biol. 21, 246–246. 10.1038/s41580-020-0219-y 32005970

[B254] Liu J. Liu W. J. Li R. L. Yang H. (2019). Mitophagy in parkinson's disease: from pathogenesis to treatment. Cells 8:712. 10.3390/cells8070712 31336937 PMC6678174

[B255] Liu J. H. Prudom C. E. Nass R. Pezzoli S. S. Oliveri M. C. Johnson M. L.. (2008). Novel ghrelin assays provide evidence for independent regulation of ghrelin acylation and secretion in healthy young men. J. Clin. Endocrinol. Metab. 93, 1980–1987. 10.1210/jc.2007-2235 18349056 PMC2386282

[B256] Liu L. Xu H. M. Jiang H. Wang J. Song N. Xie J. X. (2010). Ghrelin prevents 1-methyl-4-phenylpyridinium ion-induced cytotoxicity through antioxidation and NF-kappa B modulation in MES23.5 cells. Exp. Neurol. 222, 25–29. 10.1016/j.expneurol.2009.11.009 19931250

[B257] Liu S. W. Chen S. Ren J. Li B. J. Qin B. (2018). Ghrelin protects retinal ganglion cells against rotenone via inhibiting apoptosis, restoring mitochondrial function, and activating AKT-mTOR signaling. Neuropeptides 67, 63–70. 10.1016/j.npep.2017.11.007 29174113

[B258] Liu Y. J. McIntyre R. L. Janssens G. E. Houtkooper R. H. (2020). Mitochondrial fission and fusion: a dynamic role in aging and potential target for age-related disease. Mech. Ageing Dev. 186:111212. 10.1016/j.mad.2020.111212 32017944

[B259] Lockie S. H. Dinan T. Lawrence A. J. Spencer S. J. Andrews Z. B. (2015). Diet-induced obesity causes ghrelin resistance in reward processing tasks. Psychoneuroendocrinology 62, 114–120. 10.1016/j.psyneuen.2015.08.004 26292268

[B260] Longo K. A. Charoenthongtrakul S. Giuliana D. J. Govek E. K. McDonagh T. Qi Y.. (2008). Improved insulin sensitivity and metabolic flexibility in ghrelin receptor knockout mice. Regul. Pept. 150, 55–61. 10.1016/j.regpep.2008.03.011 18453014

[B261] Lopez M. Lage R. Saha A. K. Perez-Tilve D. Vazquez M. J. Varela L.. (2008). Hypothalamic fatty acid metabolism mediates the orexigenic action of ghrelin. Cell Metab. 7, 389–399. 10.1016/j.cmet.2008.03.006 18460330

[B262] Lopez N. E. Gaston L. Lopez K. R. Coimbra R. C. Hageny A. Putnam J.. (2012a). Early ghrelin treatment attenuates disruption of the blood brain barrier and apoptosis after traumatic brain injury through a UCP-2 mechanism. Brain Res. 1489, 140–148. 10.1016/j.brainres.2012.10.031 23099053

[B263] Lopez N. E. Krzyzaniak M. J. Blow C. Putnam J. Ortiz-Pomales Y. Hageny A. M.. (2012b). Ghrelin prevents disruption of the blood-brain barrier after traumatic brain injury. J. Neurotrauma 29, 385–393. 10.1089/neu.2011.2053 21939391 PMC3279718

[B264] Lopez-Lluch G. Hunt N. Jones B. Zhu M. Jamieson H. Hilmer S.. (2006). Calorie restriction induces mitochondrial biogenesis and bioenergetic efficiency. Proc. Natl. Acad. Sci. U.S.A. 103, 1768–1773. 10.1073/pnas.0510452103 16446459 PMC1413655

[B265] Lu X. P. Zhao X. L. Feng J. Y. Liou A. P. Anthony S. Pechhold S.. (2012). Postprandial inhibition of gastric ghrelin secretion by long-chain fatty acid through GPR120 in isolated gastric ghrelin cells and mice. Am. J. Physiol. Gastrointest. Liver Physiol. 303, G367–G376. 10.1152/ajpgi.00541.2011 22678998 PMC3774249

[B266] Lutz A. K. Exner N. Fett M. E. Schlehe J. S. Kloos K. Lammermann K. (2009). Loss of parkin or pink1 function increases Drp1-dependent mitochondrial fragmentation. J. Biol. Chem. 284, 22938–22951. 10.1074/jbc.M109.035774 19546216 PMC2755701

[B267] Lyingtunell U. Lindblad B. S. Malmlund H. O. Persson B. (1981). Cerebral blood-flow and metabolic-rate of oxygen, glucose, lactate, pyruvate, ketone-bodies and amino-acids. 2. Presenile-dementia and normal-pressure hydrocephalus. Acta Neurol. Scand. 63, 337–350 10.1111/j.1600-0404.1981.tb00788.x 7324866

[B268] Ma D. Jin S. J. Li E. D. Doi Y. Parajuli B. Noda M. (2013). The neurotoxic effect of astrocytes activated with toll-like receptor ligands. J. Neuroimmunol. 254, 10–18. 10.1016/j.jneuroim.2012.08.010 22999806

[B269] Ma X. J. M. Blenis J. (2009). Molecular mechanisms of mTOR-mediated translational control. Nat. Rev. Mol. Cell Biol. 10, 307–318. 10.1038/nrm2672 19339977

[B270] Maffei M. Halaas J. Ravussin E. Pratley R. E. Lee G. H. Zhang Y.. (1995). Leptin levels in human and rodent - measurement of plasma leptin and Ob Rna in obese and weight-reduced subjects. Nat. Med. 1, 1155–1161. 10.1038/nm1195-1155 7584987

[B271] Majumder S. Richardson A. Strong R. Oddo S. (2011). Inducing autophagy by rapamycin before, but not after, the formation of plaques and tangles ameliorates cognitive deficits. PLoS ONE 6:e25416. 10.1371/journal.pone.0025416 21980451 PMC3182203

[B272] Maldonado-Ruiz R. Montalvo-Martinez L. Fuentes-Mera L. Camacho A. (2017). Microglia activation due to obesity programs metabolic failure leading to type two diabetes. Nutr. Diabetes 7:e254. 10.1038/nutd.2017.10 28319103 PMC5380893

[B273] Mamik M. K. Power C. (2017). Inflammasomes in neurological diseases: emerging pathogenic and therapeutic concepts. Brain 140, 2273–2285. 10.1093/brain/awx133 29050380

[B274] Mani B. K. Shankar K. Zigman J. M. (2019). Ghrelin's relationship to blood glucose. Endocrinology 160, 1247–1261. 10.1210/en.2019-00074 30874792 PMC6482034

[B275] Mani B. K. Zigman J. M. (2017). Ghrelin as a survival hormone. Trends Endocrinol Metab. 28, 843–854. 10.1016/j.tem.2017.10.001 29097101 PMC5777178

[B276] Manning B. D. Cantley L. C. (2003). Rheb fills a GAP between TSC and TOR. Trends Biochem. Sci. 28, 573–576. 10.1016/j.tibs.2003.09.003 14607085

[B277] Mao Y. Q. Cheng J. Yu F. J. Li H. Q. Guo C. Y. Fan X. N. (2015). Ghrelin attenuated lipotoxicity via autophagy induction and nuclear factor-kappa B inhibition. Cell. Physiol. Biochem. 37, 563–576. 10.1159/000430377 26329041

[B278] Marleau S. Harb D. Bujold K. Avallone R. Iken K. Wang Y.. (2005). EP 80317, a ligand of the CD36 scavenger receptor, protects apolipoprotein E-deficient mice from developing atherosclerotic lesions. FASEB J. 19, 1869–1871. 10.1096/fj.04-3253fje 16123174

[B279] Martin N. M. Small C. J. Sajedi A. Patterson M. Ghatei M. A. Bloom S. R. (2004). Pre-obese and obese agouti mice are sensitive to the anorectic effects of peptide YY3-36 but resistant to ghrelin. Int. J. Obes. 28, 886–893. 10.1038/sj.ijo.0802646 15148507

[B280] Martinez-Reyes I. Chandel N. S. (2020). Mitochondrial TCA cycle metabolites control physiology and disease. Nat. Commun. 11:102. 10.1038/s41467-019-13668-3 31900386 PMC6941980

[B281] Martins I. Gomes S. Costa R. O. Otvos L. Oliveira C. R. Resende R.. (2013). Leptin and ghrelin prevent hippocampal dysfunction induced by a beta oligomers. Neuroscience 241, 41–51. 10.1016/j.neuroscience.2013.02.062 23506735

[B282] Martorana A. Koch G. (2014). Is dopamine involved in alzheimer's disease? Front. Aging Neurosci. 6:252. 10.3389/fnagi.2014.00252 25309431 PMC4174765

[B283] Matuszyk A. Ceranowicz D. Warzecha Z. Ceranowicz P. Fyderek K. Galadzka K.. (2015). The influence of ghrelin on the development of dextran sodium sulfate-induced colitis in rats. Biomed. Res. Int. 2015:718314. 10.1155/2015/718314 26713317 PMC4680107

[B284] McKenzie B. A. Mamik M. K. Saito L. B. Boghozian R. Monaco M. C. Major E. O.. (2018). Caspase-1 inhibition prevents glial inflammasome activation and pyroptosis in models of multiple sclerosis. Proc. Natl. Acad. Sci. U.S.A. 115, E6065–E6074. 10.1073/pnas.1722041115 29895691 PMC6042136

[B285] Menges S. Minakaki G. Schaefer P. M. Meixner H. Prots I. Schlotzer-Schrehardt U.. (2017). Alpha-synuclein prevents the formation of spherical mitochondria and apoptosis under oxidative stress. Sci. Rep. 7:42942. 10.1038/srep42942 28224980 PMC5320486

[B286] Meredith G. E. Rademacher D. J. (2011). MPTP mouse models of Parkinson's disease: an update. J. Parkinsons Dis. 1, 19–33. 10.3233/JPD-2011-11023 23275799 PMC3530193

[B287] Miki Y. Tanji K. Mori F. Utsumi J. Sasaki H. Kakita A.. (2016). Alteration of upstream autophagy-related proteins (ULK1, ULK2, Beclin1, VPS34 and AMBRA1) in lewy body disease. Brain Pathol. 26, 359–370. 10.1111/bpa.12297 26260450 PMC8029392

[B288] Min S. W. Sohn P. D. Li Y. Q. Devidze N. Johnson J. R. Krogan N. J.. (2018). SIRT1 deacetylates tau and reduces pathogenic tau spread in a mouse model of tauopathy. J. Neurosci. 38, 3680–3688. 10.1523/JNEUROSCI.2369-17.2018 29540553 PMC5895994

[B289] Minogue A. M. Jones R. S. Kelly R. J. McDonald C. L. Connor T. J. Lynch M. A. (2014). Age-associated dysregulation of microglial activation is coupled with enhanced blood-brain barrier permeability and pathology in APP/PS1 mice. Neurobiol. Aging 35, 1442–1452. 10.1016/j.neurobiolaging.2013.12.026 24439957

[B290] Mir J. F. Zagmutt S. Lichtenstein M. P. Garcia-Villoria J. Weber M. Gracia A.. (2018). Ghrelin causes a decline in GABA release by reducing fatty acid oxidation in cortex. Mol. Neurobiol. 55, 7216–7228. 10.1007/s12035-018-0921-3 29396649 PMC6096967

[B291] Mir M. Tolosa L. Asensio V. J. Llado J. Olmos G. (2008). Complementary roles of tumor necrosis factor alpha and interferon gamma in inducible microglial nitric oxide generation. J. Neuroimmunol. 204, 101–109. 10.1016/j.jneuroim.2008.07.002 18703234

[B292] Mizushima N. Yoshimori T. (2007). How to interpret LC3 immunoblotting. Autophagy 3, 542–545. 10.4161/auto.4600 17611390

[B293] Mohaddes G. Abdolalizadeh J. Babri S. Hossienzadeh F. (2017). Ghrelin ameliorates blood-brain barrier disruption during systemic hypoxia. Exp. Physiol. 102, 376–382. 10.1113/EP086068 28078800

[B294] Moloney A. M. Griffin R. J. Timmons S. O'Connor R. Ravid R. O'Neill C. (2010). Defects in IGF-1 receptor, insulin receptor and IRS-1/2 in alzheimer's disease indicate possible resistance to IGF-1 and insulin signalling. Neurobiol. Aging 31, 224–243. 10.1016/j.neurobiolaging.2008.04.002 18479783

[B295] Moon M. Cha M. Y. Mook-Jung I. (2014). Impaired hippocampal neurogenesis and its enhancement with ghrelin in 5XFAD mice. J. Alzheimers Dis. 41, 233–241. 10.3233/JAD-132417 24583405

[B296] Moon M. Choi J. G. Nam D. W. Hong H. S. Choi Y. J. Oh M. S. Mook-Jung I. (2011). Ghrelin ameliorates cognitive dysfunction and neurodegeneration in intrahippocampal amyloid-beta(1-42) oligomer-injected mice. J. Alzheimers Dis. 23, 147–159. 10.3233/JAD-2010-101263 20930280

[B297] Moon M. Kim H. G. Hwang L. Seo J. H. Kim S. Hwang S.. (2009a). Neuroprotective effect of ghrelin in the 1-methyl-4-phenyl-1,2,3,6-tetrahydropyridine mouse model of parkinson's disease by blocking microglial activation. Neurotox. Res. 15, 332–347. 10.1007/s12640-009-9037-x 19384567

[B298] Moon M. Kim S. Hwang L. Park S. (2009b). Ghrelin regulates hippocampal neurogenesis in adult mice. Endocr. J. 56, 525–531. 10.1507/endocrj.K09E-089 19506321

[B299] Morales I. Guzman-Martinez L. Cerda-Troncoso C. Farias G. A. Maccioni R. B. (2014). Neuroinflammation in the pathogenesis of alzheimer's disease. A rational framework for the search of novel therapeutic approaches. Front. Cell. Neurosci. 8:112. 10.3389/fncel.2014.00112 24795567 PMC4001039

[B300] Morgan A. H. Rees D. J. Andrews Z. B. Davies J. S. (2018). Ghrelin mediated neuroprotection - a possible therapy for parkinson's disease? Neuropharmacology 136, 317–326. 10.1016/j.neuropharm.2017.12.027 29277488

[B301] Moroo I. Yamada T. Makino H. Tooyama I. Mcgeer P. L. Mcgeer E. G.. (1994). Loss of insulin-receptor immunoreactivity from the substantia-nigra pars-compacta neurons in parkinsons-disease. Acta Neuropathol. 87, 343–348 10.1007/BF00313602 8017169

[B302] Morris J. K. Vidoni E. D. Perea R. D. Rada R. Johnson D. K. Lyons K.. (2014). Insulin resistance and gray matter volume in neurodegenerative disease. Neuroscience 270, 139–147. 10.1016/j.neuroscience.2014.04.006 24735819 PMC4211112

[B303] Morrow V. A. Foufelle F. Connell J. M. C. Petrie J. R. Gould G. W. Salt I. P. (2003). Direct activation of AMP-activated protein kinase stimulates nitric-oxide synthesis in human aortic endothelial cells. J. Biol. Chem. 278, 31629–31639. 10.1074/jbc.M212831200 12791703

[B304] Mosconi L. Pupi A. De Leon M. J. (2008). Brain glucose hypometabolism and oxidative stress in preclinical alzheimer's disease. Mitochondria Oxidat. Stress. Neurodegener. Disord. 1147, 180–195. 10.1196/annals.1427.007 19076441 PMC2661241

[B305] Muddapu V. R. Dharshini S. A. P. Chakravarthy V. S. Gromiha M. M. (2020). Neurodegenerative diseases - is metabolic deficiency the root cause? Front. Neurosci. 14:213. 10.3389/fnins.2020.00213 32296300 PMC7137637

[B306] Mudo G. Makela J. Di Liberto V. Tselykh T. V. Olivieri M. Piepponen P.. (2012). Transgenic expression and activation of PGC-1 alpha protect dopaminergic neurons in the MPTP mouse model of parkinson's disease. Cell. Mol. Life Sci. 69, 1153–1165. 10.1007/s00018-011-0850-z 21984601 PMC11114858

[B307] Muller T. D. Nogueiras R. Andermann M. L. Andrews Z. B. Anker S. D. Argente J. (2015). Ghrelin. Mol. Metab. 4, 437–460. 10.1016/j.molmet.2015.03.005 26042199 PMC4443295

[B308] Murtuza M. I. Isokawa M. (2018). Endogenous ghrelin-O-acyltransferase (GOAT) acylates local ghrelin in the hippocampus. J. Neurochem. 144, 58–67. 10.1111/jnc.14244 29063591 PMC5832437

[B309] Mussa B. M. Verberne A. J. M. (2013). The dorsal motor nucleus of the vagus and regulation of pancreatic secretory function. Exp. Physiol. 98, 25–37. 10.1113/expphysiol.2012.066472 22660814

[B310] Nagaya N. Kojima M. Uematsu M. Yamagishi M. Hosoda H. Oya H.. (2001). Hemodynamic and hormonal effects of human ghrelin in healthy volunteers. Am. J. Physiol. Regul. Integr. Comp. Physiol. 280, R1483–R1487 10.1152/ajpregu.2001.280.5.R1483 11294772

[B311] Nagaya N. Moriya J. Yasumura Y. Uematsu M. Ono F. Shimizu W.. (2004). Effects of ghrelin administration on left ventricular function, exercise capacity, and muscle wasting in patients with chronic heart failure. Circulation 110, 3674–3679. 10.1161/01.CIR.0000149746.62908.BB 15569841

[B312] Najem D. Bamji-Mirza M. Chang N. N. Liu Q. Y. Zhang W. D. (2014). Insulin resistance, neuroinflammation, and alzheimer's disease. Rev. Neurosci. 25, 509–525. 10.1515/revneuro-2013-0050 24622783

[B313] Najem D. Bamji-Mirza M. Yang Z. Zhang W. (2016). Abeta-induced insulin resistance and the effects of insulin on the cholesterol synthesis pathway and abeta secretion in neural cells. Neurosci. Bull. 32, 227–238. 10.1007/s12264-016-0034-9 27207326 PMC5563777

[B314] Nakae J. Oki M. Cao Y. H. (2008). The FoxO transcription factors and metabolic regulation. FEBS Lett. 582, 54–67. 10.1016/j.febslet.2007.11.025 18022395

[B315] Nakamura K. Nemani V. M. Azarbal F. Skibinski G. Levy J. M. Egami K.. (2011). Direct membrane association drives mitochondrial fission by the parkinson disease-associated protein alpha-synuclein. J. Biol. Chem. 286, 20710–20726. 10.1074/jbc.M110.213538 21489994 PMC3121472

[B316] Nass R. Gaylinn B. D. Thorner M. O. (2011). The role of ghrelin in GH secretion and GH disorders. Mol. Cell. Endocrinol. 340, 10–14. 10.1016/j.mce.2011.03.021 21459126 PMC4205082

[B317] Nass R. Pezzoli S. S. Oliveri M. C. Patrie J. T. Harrell F. E. Jr. Clasey J. L.. (2008). Effects of an oral ghrelin mimetic on body composition and clinical outcomes in healthy older adults: a randomized trial. Ann. Intern. Med. 149, 601–611. 10.7326/0003-4819-149-9-200811040-00003 18981485 PMC2757071

[B318] Naznin F. Toshinai K. Waise T. M. Z. NamKoong C. Moin A. S. M. Sakoda H.. (2015). Diet-induced obesity causes peripheral and central ghrelin resistance by promoting inflammation. J. Endocrinol. 226, 81–92. 10.1530/JOE-15-0139 26016745 PMC4485401

[B319] Naznin F. Toshinai K. Waise T. M. Z. Okada T. Sakoda H. Nakazato M. (2018). Restoration of metabolic inflammation-related ghrelin resistance by weight loss. J. Mol. Endocrinol. 60, 109–118. 10.1530/JME-17-0192 29233861 PMC5793712

[B320] Neth B. J. Craft S. (2017). Insulin resistance and alzheimer's disease: bioenergetic linkages. Front. Aging Neurosci. 9:345. 10.3389/fnagi.2017.00345 29163128 PMC5671587

[B321] Nixon R. A. Wegiel J. Kumar A. Yu W. H. Peterhoff C. Cataldo A.. (2005). Extensive involvement of autophagy in alzheimer disease: an immuno-electron microscopy study. J. Neuropathol. Exp. Neurol. 64, 113–122. 10.1093/jnen/64.2.113 15751225

[B322] Ogawa M. Fukuyama H. Ouchi Y. Yamauchi H. Kimura J. (1996). Altered energy metabolism in alzheimer's disease. J. Neurol. Sci. 139, 78–82. 10.1016/0022-510X(96)00033-0 8836976

[B323] Okumura T. Taniguchi A. Nagasaka S. Sakai M. Fukushima M. Kuroe A.. (2003). Relationship of regional adiposity to serum leptin level in nonobese Japanese type 2 diabetic male patients. Diabetes Metab. 29, 15–18. 10.1016/S1262-3636(07)70002-2 12629443

[B324] Onyango I. G. Dennis J. Khan S. M. (2016). Mitochondrial dysfunction in alzheimer's disease and the rationale for bioenergetics based therapies. Aging Dis. 7, 201–214. 10.14336/AD.2015.1007 27114851 PMC4809610

[B325] Ortega-Martinez S. (2015). A new perspective on the role of the CREB family of transcription factors in memory consolidation via adult hippocampal neurogenesis. Front. Mol. Neurosci. 8:46. 10.3389/fnmol.2015.00046 26379491 PMC4549561

[B326] Pamukcu O. Kumral Z. N. O. Ercan F. Yegen B. C. Ertem D. (2013). Anti-inflammatory effect of obestatin and ghrelin in dextran sulfate sodium-induced colitis in rats. J. Pediatr. Gastroenterol. Nutr. 57, 211–218. 10.1097/MPG.0b013e318294711e 23549326

[B327] Pan T. H. Kondo S. Zhu W. Xie W. J. Jankovic J. Le W. D. (2008). Neuroprotection of rapamycin in lactacystin-induced neurodegeneration via autophagy enhancement. Neurobiol. Dis. 32, 16–25. 10.1016/j.nbd.2008.06.003 18640276

[B328] Pan W. H. Tu H. Kastin A. J. (2006). Differential BBB interactions of three ingestive peptides: obestatin, ghrelin, and adiponectin. Peptides 27, 911–916. 10.1016/j.peptides.2005.12.014 16476508

[B329] Panaro M. A. Lofrumento D. D. Saponaro C. De Nuccio F. Cianciulli A. Mitolo V.. (2008). Expression of TLR4 and CD14 in the central nervous system (CNS) in a MPTP mouse model of parkinson's-like disease. Immunopharmacol. Immunotoxicol. 30, 729–740. 10.1080/08923970802278557 18686098

[B330] Pandey G. Shihabudeen M. S. David H. P. Thirumurugan E. Thirumurugan K. (2015). Association between hyperleptinemia and oxidative stress in obese diabetic subjects. J. Diabetes Metab. Disord. 14:24. 10.1186/s40200-015-0159-9 25897417 PMC4404074

[B331] Paneda C. Arroba A. I. Frago L. M. Holm A. M. Romer J. Argente J.. (2003). Growth hormone-releasing peptide-6 inhibits cerebellar cell death in aged rats. Neuroreport 14, 1633–1635. 10.1097/00001756-200308260-00018 14502090

[B332] Parkinson Study Group Electronic address pfeiffro@ohsu.edu. (2017). A randomized trial of relamorelin for constipation in parkinson's disease (MOVE-PD): trial results and lessons learned. Parkinsonism Relat. Disord. 37, 101–105. 10.1016/j.parkreldis.2017.02.003 28237854

[B333] Pedros I. Petrov D. Allgaier M. Sureda F. Barroso E. Beas-Zarate C.. (2014). Early alterations in energy metabolism in the hippocampus of APPswe/PS1dE9 mouse model of alzheimer's disease. Biochim. Biophys. Acta Mol. Basis Dis. 1842, 1556–1566. 10.1016/j.bbadis.2014.05.025 24887203

[B334] Peixoto C. A. de Oliveira W. H. Araujo S. M. D. Nunes A. K. S. (2017). AMPK activation: role in the signaling pathways of neuroinflammation and neurodegeneration. Exp. Neurol. 298, 31–41. 10.1016/j.expneurol.2017.08.013 28844606

[B335] Peng K. G. Yang L. K. Wang J. Ye F. Dan G. R. Zhao Y. P. (2017). The interaction of mitochondrial biogenesis and fission/fusion mediated by PGC-1 alpha regulates rotenone-induced dopaminergic neurotoxicity. Mol. Neurobiol. 54, 3783–3797. 10.1007/s12035-016-9944-9 27271125

[B336] Penicaud L. Leloup C. Lorsignol A. Alquier T. Guillod E. (2002). Brain glucose sensing mechanism and glucose homeostasis. Curr. Opin. Clin. Nutr. Metab. Care 5, 539–543. 10.1097/00075197-200209000-00013 12172478

[B337] Perello M. Scott M. M. Sakata I. Lee C. E. Chuang J. C. Osborne-Lawrence S.. (2012). Functional implications of limited leptin receptor and ghrelin receptor coexpression in the brain. J. Comp. Neurol. 520, 281–294. 10.1002/cne.22690 21674492 PMC3282302

[B338] Perez-Tilve D. Heppner K. Kirchner H. Lockie S. H. Woods S. C. Smiley D. L.. (2011). Ghrelin-induced adiposity is independent of orexigenic effects. Faseb J. 25, 2814–2822. 10.1096/fj.11-183632 21543764 PMC3136335

[B339] Perreault M. Istrate N. Wang L. Nichols A. J. Tozzo E. Stricker-Krongrad A. (2004). Resistance to the orexigenic effect of ghrelin in dietary-induced obesity in mice: reversal upon weight loss. Int. J. Obes. 28, 879–885. 10.1038/sj.ijo.0802640 15111983

[B340] Pickford F. Masliah E. Britschgi M. Lucin K. Narasimhan R. Jaeger P. A.. (2008). The autophagy-related protein beclin 1 shows reduced expression in early alzheimer disease and regulates amyloid beta accumulation in mice. J. Clin. Invest. 118, 2190–2199. 10.1172/JCI33585 18497889 PMC2391284

[B341] Pickrell A. M. Youle R. J. (2015). The roles of PINK1, parkin, and mitochondrial fidelity in parkinson's disease. Neuron 85, 257–273. 10.1016/j.neuron.2014.12.007 25611507 PMC4764997

[B342] Pisani V. Stefani A. Pierantozzi M. Natoli S. Stanzione P. Franciotta D.. (2012). Increased blood-cerebrospinal fluid transfer of albumin in advanced parkinson's disease. J. Neuroinflammation 9:188. 10.1186/1742-2094-9-188 22870899 PMC3441323

[B343] Poewe W. Seppi K. Tanner C. M. Halliday G. M. Brundin P. Volkmann J. (2017). Parkinson disease. Nat. Rev. Dis. Primers 3:17013 10.1038/nrdp.2017.13 28332488

[B344] Price C. J. Hoyda T. D. Ferguson A. V. (2008). The area postrema: a brain monitor and integrator of systemic autonomic state. Neuroscientist 14, 182–194. 10.1177/1073858407311100 18079557

[B345] Proto C. Romualdi D. Cento R. M. Spada R. S. Di Mento G. Ferri R.. (2006). Plasma levels of neuropeptides in alzheimer's disease. Gynecol. Endocrinol. 22, 213–218. 10.1080/09513590500519385 16723308

[B346] Qi Y. Longo K. A. Giuliana D. J. Gagne S. McDonagh T. Govek E.. (2011). Characterization of the insulin sensitivity of ghrelin receptor KO mice using glycemic clamps. BMC Physiol 11:1. 10.1186/1472-6793-11-1 21211044 PMC3024223

[B347] Quan Y. Xin Y. Tian G. Zhou J. Liu X. (2020). Mitochondrial ROS-modulated mtDNA: a potential target for cardiac aging. Oxid. Med. Cell. Longev. 2020:9423593. 10.1155/2020/9423593 32308810 PMC7139858

[B348] Quarta D. Di Francesco C. Melotto S. Mangiarini L. Heidbreder C. Hedou G. (2009). Systemic administration of ghrelin increases extracellular dopamine in the shell but not the core subdivision of the nucleus accumbens. Neurochem. Int. 54, 89–94. 10.1016/j.neuint.2008.12.006 19118592

[B349] Reed J. A. Benoit S. C. Pfluger P. T. Tschoep M. H. D'Alessio D. A. Seeley R. J. (2008). Mice with chronically increased circulating ghrelin develop age-related glucose intolerance. Am. J. Physiol. Endocrinol. Metab. 294, E752–E760. 10.1152/ajpendo.00463.2007 18270302

[B350] Reed-Geaghan E. G. Savage J. C. Hise A. G. Landreth G. E. (2009). CD14 and toll-like receptors 2 and 4 are required for fibrillar A{beta}-stimulated microglial activation. J. Neurosci. 29, 11982–11992. 10.1523/JNEUROSCI.3158-09.2009 19776284 PMC2778845

[B351] Reynolds A. D. Stone D. K. Hutter J. A. L. Benner E. J. Mosley R. L. Gendelman H. E. (2010). Regulatory T cells attenuate Th17 cell-mediated nigrostriatal dopaminergic neurodegeneration in a model of parkinson's disease. J. Immunol. 184, 2261–2271. 10.4049/jimmunol.0901852 20118279 PMC2824790

[B352] Richartz-Salzburger E. Batra A. Stransky E. Laske C. Kohler N. Bartels M.. (2007). Altered lymphocyte distribution in alzheimer's disease. J. Psychiatr. Res. 41, 174–178. 10.1016/j.jpsychires.2006.01.010 16516234

[B353] Rigamonti A. E. Pincelli A. I. Corra B. Viarengo R. Bonomo S. M. Galimberti D.. (2002). Plasma ghrelin concentrations in elderly subjects: comparison with anorexic and obese patients. J. Endocrinol. 175, R1–R5. 10.1677/joe.0.175r001 12379512

[B354] Roher A. E. Debbins J. P. Malek-Ahmadi M. Chen K. Pipe J. G. Maze S.. (2012). Cerebral blood flow in alzheimer's disease. Vasc. Health Risk Manag. 8, 599–611. 10.2147/VHRM.S34874 23109807 PMC3481957

[B355] Ruozi G. Bortolotti F. Falcione A. Dal Ferro M. Ukovich L. Macedo A.. (2015). AAV-mediated *in vivo* functional selection of tissue-protective factors against ischaemia. Nat. Commun. 6:8388. 10.1038/ncomms8388 26066847 PMC4477044

[B356] Sakata I. Park W. M. Walker A. K. Piper P. K. Chuang J. C. Osborne-Lawrence S.. (2012). Glucose-mediated control of ghrelin release from primary cultures of gastric mucosal cells. Am. J. Physiol. Endocrinol. Metab. 302, E1300–E1310. 10.1152/ajpendo.00041.2012 22414807 PMC3361986

[B357] Saleiro D. Platanias L. C. (2015). Intersection of mTOR and STAT signaling in immunity. Trends Immunol. 36, 21–29. 10.1016/j.it.2014.10.006 25592035 PMC4297313

[B358] Sankowski R. Mader S. Valdes-Ferrer S. I. (2015). Systemic inflammation and the brain: novel roles of genetic, molecular, and environmental cues as drivers of neurodegeneration. Front. Cell. Neurosci. 9:28. 10.3389/fncel.2015.00028 25698933 PMC4313590

[B359] Santos V. V. Stark R. Rial D. Silva H. B. Bayliss J. A. Lemus M. B.. (2017). Acyl ghrelin improves cognition, synaptic plasticity deficits and neuroinflammation following amyloid beta (A beta 1-40) administration in mice. J. Neuroendocrinol. 29, 1–11. 10.1111/jne.12476 28380673

[B360] Saresella M. Calabrese E. Marventano I. Piancone F. Gatti A. Alberoni M.. (2011). Increased activity of Th-17 and Th-9 lymphocytes and a skewing of the post-thymic differentiation pathway are seen in alzheimer's disease. Brain Behav. Immun. 25, 539–547. 10.1016/j.bbi.2010.12.004 21167930

[B361] Satou M. Nishi Y. Yoh J. Hattori Y. Sugimoto H. (2010). Identification and characterization of acyl-protein thioesterase 1/lysophospholipase i as a ghrelin deacylation/lysophospholipid hydrolyzing enzyme in fetal bovine serum and conditioned medium. Endocrinology 151, 4765–4775. 10.1210/en.2010-0412 20685872

[B362] Saunders J. A. H. Estes K. A. Kosloski L. M. Allen H. E. Dempsey K. M. Torres-Russotto D. R.. (2012). CD4+regulatory and effector/memory t cell subsets profile motor dysfunction in parkinson's disease. J. Neuroimmune Pharmacol. 7, 927–938. 10.1007/s11481-012-9402-z 23054369 PMC3515774

[B363] Scarpulla R. C. (2002). Transcriptional activators and coactivators in the nuclear control of mitochondrial function in mammalian cells. Gene 286, 81–89. 10.1016/S0378-1119(01)00809-5 11943463

[B364] Scarpulla R. C. (2006). Nuclear control of respiratory gene expression in mammalian cells. J. Cell. Biochem. 97, 673–683. 10.1002/jcb.20743 16329141

[B365] Schlaepfer I. R. Joshi M. (2020). CPT1A-mediated fat oxidation, mechanisms, and therapeutic potential. Endocrinology 161:bqz046. 10.1210/endocr/bqz046 31900483

[B366] Schopfer L. M. Lockridge O. Brimijoin S. (2015). Pure human butyrylcholinesterase hydrolyzes octanoyl ghrelin to desacyl ghrelin. Gen. Comp. Endocrinol. 224, 61–68. 10.1016/j.ygcen.2015.05.017 26073531

[B367] Schubert M. Brazil D. P. Burks D. J. Kushner J. A. Ye J. Flint C. L.. (2003). Insulin receptor substrate-2 deficiency impairs brain growth and promotes tau phosphorylation. J. Neurosci. 23, 7084–7092. 10.1523/JNEUROSCI.23-18-07084.2003 12904469 PMC6740672

[B368] Schubert M. Gautam D. Surjo D. Ueki K. Baudler S. Schubert D.. (2004). Role for neuronal insulin resistance in neurodegenerative diseases. Proc. Natl. Acad. Sci. U.S.A. 101, 3100–3105. 10.1073/pnas.0308724101 14981233 PMC365750

[B369] Schurr A. West C. A. Rigor B. M. (1988). Lactate-supported synaptic function in the rat hippocampal slice preparation. Science 240, 1326–1328. 10.1126/science.3375817 3375817

[B370] Serrenho D. Santos S. D. Carvalho A. L. (2019). The role of ghrelin in regulating synaptic function and plasticity of feeding-associated circuits. Front. Cell. Neurosci. 13:205. 10.3389/fncel.2019.00205 31191250 PMC6546032

[B371] Shalit F. Sredni B. Brodie C. Kott E. Huberman M. (1995). T-lymphocyte subpopulations and activation markers correlate with severity of alzheimers-disease. Clin. Immunol. Immunopathol. 75, 246–250. 10.1006/clin.1995.1078 7768042

[B372] Shi L. M. Bian X. L. Qu Z. Q. Ma Z. G. Zhou Y. Wang K. W.. (2013). Peptide hormone ghrelin enhances neuronal excitability by inhibition of Kv7/KCNQ channels. Nat. Commun. 4 :2439. 10.1038/ncomms2439 23385580

[B373] Shi L. M. Du X. X. Jiang H. Xie J. X. (2017). Ghrelin and neurodegenerative disorders-a review. Mol. Neurobiol. 54, 1144–1155. 10.1007/s12035-016-9729-1 26809582

[B374] Shiiya T. Nakazato M. Mizuta M. Date Y. Mondal M. S. Tanaka M.. (2002). Plasma ghrelin levels in lean and obese humans and the effect of glucose on ghrelin secretion. J. Clin. Endocrinol. Metab. 87, 240–244. 10.1210/jcem.87.1.8129 11788653

[B375] Shimizu Y. Nagaya N. Teranishi Y. Imazu M. Yamamoto H. Shokawa T.. (2003). Ghrelin improves endothelial dysfunction through growth hormone-independent mechanisms in rats. Biochem. Biophys. Res. Commun. 310, 830–835. 10.1016/j.bbrc.2003.09.085 14550279

[B376] Shin J. H. Ko H. S. Kang H. Lee Y. Lee Y. I. Pletinkova O. (2011). PARIS (ZNF746) repression of PGC-1 alpha contributes to neurodegeneration in parkinson's disease. Cell 144, 689–702. 10.1016/j.cell.2011.02.010 21376232 PMC3063894

[B377] Sirago G. Conte E. Fracasso F. Cormio A. Fehrentz J. A. Martinez J.. (2017). Growth hormone secretagogues hexarelin and JMV2894 protect skeletal muscle from mitochondrial damages in a rat model of cisplatin-induced cachexia. Sci. Rep. 7:13017. 10.1038/s41598-017-13504-y 29026190 PMC5638899

[B378] Skibicka K. P. Hansson C. Alvarez-Crespo M. Friberg P. A. Dickson S. L. (2011). Ghrelin directly targets the ventral tegmental area to increase food motivation. Neuroscience 180, 129–137. 10.1016/j.neuroscience.2011.02.016 21335062

[B379] Skibicka K. P. Hansson C. Egecioglu E. Dickson S. L. (2012). Role of ghrelin in food reward: impact of ghrelin on sucrose self-administration and mesolimbic dopamine and acetylcholine receptor gene expression. Addict. Biol. 17, 95–107. 10.1111/j.1369-1600.2010.00294.x 21309956 PMC3298643

[B380] Slupecka M. Wolinski J. Pierzynowski S. G. (2012). The effects of enteral ghrelin administration on the remodeling of the small intestinal mucosa in neonatal piglets. Regul. Pept. 174, 38–45. 10.1016/j.regpep.2011.11.007 22137939

[B381] Sochocka M. Zwolinska K. Leszek J. (2017). The infectious etiology of alzheimer's disease. Curr. Neuropharmacol. 15, 996–1009. 10.2174/1570159X15666170313122937 28294067 PMC5652018

[B382] Soriano F. X. Liesa M. Bach D. Chan D. C. Palacin M. Zorzano A. (2006). Evidence for a mitochondrial regulatory pathway defined by peroxisome proliferator-activated receptor-gamma coactivator-1 alpha, estrogen-related receptor-alpha, and mitofusin 2. Diabetes 55, 1783–1791. 10.2337/db05-0509 16731843

[B383] Souza-Moreira L. Delgado-Maroto V. Morell M. O'Valle F. Del Moral R. G. Gonzalez-Rey E. (2013). Therapeutic effect of ghrelin in experimental autoimmune encephalomyelitis by inhibiting antigen-specific Th1/Th17 responses and inducing regulatory T cells. Brain Behav. Immun. 30, 54–60. 10.1016/j.bbi.2013.01.080 23376169

[B384] Speciale L. Calabrese E. Saresella M. Tinelli C. Mariani C. Sanvito L.. (2007). Lymphocyte subset patterns and cytokine production in alzheimer's disease patients. Neurobiol. Aging 28, 1163–1169. 10.1016/j.neurobiolaging.2006.05.020 16814429

[B385] Spencer B. Potkar R. Trejo M. Rockenstein E. Patrick C. Gindi R.. (2009). Beclin 1 gene transfer activates autophagy and ameliorates the neurodegenerative pathology in alpha-synuclein models of parkinson's and lewy body diseases. J. Neurosci. 29, 13578–13588. 10.1523/JNEUROSCI.4390-09.2009 19864570 PMC2812014

[B386] Spencer S. J. Miller A. A. Andrews Z. B. (2013). The role of ghrelin in neuroprotection after ischemic brain injury. Brain Sci. 3, 344–359. 10.3390/brainsci3010344 24961317 PMC4061836

[B387] Spilman P. Podlutskaya N. Hart M. J. Debnath J. Gorostiza O. Bredesen D. (2010). Inhibition of mTOR by rapamycin abolishes cognitive deficits and reduces amyloid-beta levels in a mouse model of alzheimer's disease. PLoS ONE 5:e9979 10.1371/journal.pone.0009979 20376313 PMC2848616

[B388] St.-Pierre J. Drori S. Uldry M. Silvaggi J. M. Rhee J. Jager S.. (2006). Suppression of reactive oxygen species and neurodegeneration by the PGC-1 transcriptional coactivators. Cell 127, 397–408. 10.1016/j.cell.2006.09.024 17055439

[B389] Stancu I. C. Cremers N. Vanrusselt H. Couturier J. Vanoosthuyse A. Kessels S.. (2019). Aggregated Tau activates NLRP3-ASC inflammasome exacerbating exogenously seeded and non-exogenously seeded Tau pathology *in vivo*. Acta Neuropathol. 137, 599–617. 10.1007/s00401-018-01957-y 30721409 PMC6426830

[B390] Steen E. Terry B. M. Rivera E. J. Cannon J. L. Neely T. R. Tavares R.. (2005). Impaired insulin and insulin-like growth factor expression and signaling mechanisms in alzheimer's disease - is this type 3 diabetes? J. Alzheimers Dis. 7, 63–80. 10.3233/JAD-2005-7107 15750215

[B391] Stephenson J. Nutma E. van der Valk P. Amor S. (2018). Inflammation in CNS neurodegenerative diseases. Immunology 154, 204–219. 10.1111/imm.12922 29513402 PMC5980185

[B392] Stoyanova I. I. (2014). Ghrelin: a link between ageing, metabolism and neurodegenerative disorders. Neurobiol. Dis. 72, 72–83. 10.1016/j.nbd.2014.08.026 25173805

[B393] Stranahan A. M. Norman E. D. Lee K. Cutler R. G. Telljohann R. S. Egan J. M.. (2008). Diet-induced insulin resistance impairs hippocampal synaptic plasticity and cognition in middle-aged rats. Hippocampus 18, 1085–1088. 10.1002/hipo.20470 18651634 PMC2694409

[B394] Subiros N. Perez-Saad H. M. Berlanga J. A. Aldana L. Garcia-Illera G. Gibson C. L.. (2016). Assessment of dose-effect and therapeutic time window in preclinical studies of rhEGF and GHRP-6 coadministration for stroke therapy. Neurol. Res. 38, 187–195. 10.1179/1743132815Y.0000000089 26311576

[B395] Suda Y. Kuzumaki N. Narita M. Hamada Y. Shibasaki M. Tanaka K. (2018). Effect of ghrelin on the motor deficit caused by the ablation of nigrostriatal dopaminergic cells or the inhibition of striatal dopamine receptors. Biochem. Biophys. Res. Commun. 496, 1102–1108. 10.1016/j.bbrc.2018.01.145 29378186

[B396] Sulzer D. Alcalay R. N. Garretti F. Cote L. Kanter E. Agin-Liebes J. (2017). T cells from patients with parkinson's disease recognize alpha-synuclein peptides. Nature 550, 292–292. 10.1038/nature23896 PMC10204083 28905919

[B397] Sun Y. Asnicar M. Saha P. K. Chan L. Smith R. G. (2006). Ablation of ghrelin improves the diabetic but not obese phenotype of ob/ob mice. Cell Metab. 3, 379–386. 10.1016/j.cmet.2006.04.004 16679295

[B398] Suzuki K. Uchida K. Nakanishi N. Hattori Y. (2008). Cilostazol activates AMP-activated protein kinase and restores endothelial function in diabetes. Am. J. Hypertens. 21, 451–457. 10.1038/ajh.2008.6 18369362

[B399] Sweeney M. D. Sagare A. P. Zlokovic B. V. (2018). Blood-brain barrier breakdown in alzheimer disease and other neurodegenerative disorders. Nat. Rev. Neurol. 14, 133–150. 10.1038/nrneurol.2017.188 29377008 PMC5829048

[B400] Takahashi M. Yamada T. Tooyama I. Moroo I. Kimura H. Yamamoto T.. (1996). Insulin receptor mRNA in the substantia nigra in parkinson's disease. Neurosci. Lett. 204, 201–204. 10.1016/0304-3940(96)12357-0 8938265

[B401] Takata A. Takiguchi S. Miyazaki Y. Miyata H. Takahashi T. Kurokawa Y.. (2015). Randomized phase II study of the anti-inflammatory effect of ghrelin during the postoperative period of esophagectomy. Ann. Surg. 262, 230–236. 10.1097/SLA.0000000000000986 25361222

[B402] Talbot K. Wang H. Y. Kazi H. Han L. Y. Bakshi K. P. Stucky A.. (2012). Demonstrated brain insulin resistance in alzheimer's disease patients is associated with IGF-1 resistance, IRS-1 dysregulation, and cognitive decline. J. Clin. Invest. 122, 1316–1338. 10.1172/JCI59903 22476197 PMC3314463

[B403] Tamaki M. Hagiwara A. Miyashita K. Wakino S. Inoue H. Fujii K.. (2015). Improvement of physical decline through combined effects of muscle enhancement and mitochondrial activation by a gastric hormone ghrelin in male 5/6Nx CKD model mice. Endocrinology 156, 3638–3648. 10.1210/en.2015-1353 26241123

[B404] Tanji K. Mori F. Kakita A. Takahashi H. Wakabayashi K. (2011). Alteration of autophagosomal proteins (LC3, GABARAP and GATE-16) in lewy body disease. Neurobiol. Dis. 43, 690–697. 10.1016/j.nbd.2011.05.022 21684337

[B405] Tanno M. Sakamoto J. Miura T. Shimamoto K. Horio Y. (2007). Nucleocytoplasmic shuttling of the NAD+-dependent histone deacetylase SIRT1. J. Biol. Chem. 282, 6823–6832. 10.1074/jbc.M609554200 17197703

[B406] Tateya S. Kim F. Tamori Y. (2013). Recent advances in obesity-induced inflammation and insulin resistance. Front. Endocrinol. 4:93. 10.3389/fendo.2013.00093 23964268 PMC3737462

[B407] Tesauro M. Schinzari F. Iantorno M. Rizza S. Melina D. Lauro D.. (2005). Ghrelin improves endothelial function in patients with metabolic syndrome. Circulation 112, 2986–2992. 10.1161/CIRCULATIONAHA.105.553883 16260640

[B408] Theander-Carrillo C. Wiedmer P. Cettour-Rose P. Nogueiras R. Perez-Tilve D. Pfluger P.. (2006). Ghrelin action in the brain controls adipocyte metabolism. J. Clin. Invest. 116, 1983–1993. 10.1172/JCI25811 16767221 PMC1474815

[B409] Theil M. M. Miyake S. Mizuno M. Tomi C. Croxford J. L. Hosoda H.. (2009). Suppression of experimental autoimmune encephalomyelitis by ghrelin. J. Immunol. 183, 2859–2866. 10.4049/jimmunol.0803362 19620309

[B410] TheodoropoulouA.MetallinosI. C.PsyrogiannisA.VagenakisG. A.KyriazopoulouV. (2012). Ghrelin and leptin secretion in patients with moderate alzheimer's disease. J. Nutr. Health Aging 16, 472–477. 10.1007/s12603-012-0058-422555794

[B411] Tong J. Dave N. Mugundu G. M. Davis H. W. Gaylinn B. D. Thorner M. O.. (2013). The pharmacokinetics of acyl, des-acyl, and total ghrelin in healthy human subjects. Eur. J. Endocrinol. 168, 821–828. 10.1530/EJE-13-0072 23482590 PMC3740531

[B412] Tong J. Davis H. W. Summer S. Benoit S. C. Haque A. Bidlingmaier M. (2014). Acute administration of unacylated ghrelin has no effect on basal or stimulated insulin secretion in healthy humans. Diabetes 63, 2309–2319. 10.2337/db13-1598 24550190 PMC4066344

[B413] Tong J. Prigeon R. L. Davis H. W. Bidlingmaier M. Kahn S. E. Cummings D. E.. (2010). Ghrelin suppresses glucose-stimulated insulin secretion and deteriorates glucose tolerance in healthy humans. Diabetes 59, 2145–2151. 10.2337/db10-0504 20584998 PMC2927935

[B414] Tong M. Dong M. de la Monte S. M. (2009). Brain insulin-like growth factor and neurotrophin resistance in parkinson's disease and dementia with lewy bodies: potential role of manganese neurotoxicity. J. Alzheimers Dis. 16, 585–599. 10.3233/JAD-2009-0995 19276553 PMC2852260

[B415] Tong X. X. Wu D. Wang X. Chen H. L. Chen J. X. Wang X. X.. (2012). Ghrelin protects against cobalt chloride-induced hypoxic injury in cardiac H9c2 cells by inhibiting oxidative stress and inducing autophagy. Peptides 38, 217–227. 10.1016/j.peptides.2012.06.020 23000094

[B416] Toyama E. Q. Herzig S. Courchet J. Lewis T. L. Loson O. C. Hellberg K.. (2016). AMP-activated protein kinase mediates mitochondrial fission in response to energy stress. Science 351, 275–281. 10.1126/science.aab4138 26816379 PMC4852862

[B417] Tracey T. J. Steyn F. J. Wolvetang E. J. Ngo S. T. (2018). Neuronal lipid metabolism: multiple pathways driving functional outcomes in health and disease. Front. Mol. Neurosci. 11:10. 10.3389/fnmol.2018.00010 29410613 PMC5787076

[B418] Tschop M. Weyer C. Tataranni P. A. Devanarayan V. Ravussin E. Heiman M. L. (2001). Circulating ghrelin levels are decreased in human obesity. Diabetes 50, 707–709. 10.2337/diabetes.50.4.707 11289032

[B419] Unger M. M. Moller J. C. Mankel K. Eggert K. M. Bohne K. Bodden M.. (2011). Postprandial ghrelin response is reduced in patients with parkinson's disease and idiopathic REM sleep behaviour disorder: a peripheral biomarker for early parkinson's disease? J. Neurol. 258, 982–990. 10.1007/s00415-010-5864-1 21181542

[B420] Velasquez D. A. Martinez G. Romero A. Vazquez M. J. Boit K. D. Dopeso-Reyes I. G.. (2011). The central sirtuin 1/p53 pathway is essential for the orexigenic action of ghrelin. Diabetes 60, 1177–1185. 10.2337/db10-0802 21386086 PMC3064091

[B421] Vestergaard E. T. Hansen T. K. Gormsen L. C. Jakobsen P. Moller N. Christiansen J. S.. (2007). Constant intravenous ghrelin infusion in healthy young men: clinical pharmacokinetics and metabolic effects. Am. J. Physiol. Endocrinol. Metab. 292, E1829–E1836. 10.1152/ajpendo.00682.2006 17311892

[B422] Virdis A. Duranti E. Colucci R. Ippolito C. Tirotta E. Lorenzini G.. (2015). Ghrelin restores nitric oxide availability in resistance circulation of essential hypertensive patients: role of NAD(P)H oxidase. Eur. Heart J. 36, 3023–3030. 10.1093/eurheartj/ehv365 26224075

[B423] Walker A. K. Rivera P. D. Wang Q. Chuang J. C. Tran S. Osborne-Lawrence S.. (2015). The P7C3 class of neuroprotective compounds exerts antidepressant efficacy in mice by increasing hippocampal neurogenesis. Mol. Psychiatry 20, 500–508. 10.1038/mp.2014.34 24751964 PMC4206684

[B424] Wan S. X. Shi B. Lou X. L. Liu J. Q. Mae G. G. Liang D. Y.. (2016). Ghrelin protects small intestinal epithelium against sepsis-induced injury by enhancing the autophagy of intestinal epithelial cells. Biomed. Pharmacother. 83, 1315–1320. 10.1016/j.biopha.2016.08.048 27571874

[B425] Wang J. T. Song Y. T. Chen Z. Leng S. A. X. (2018). Connection between systemic inflammation and neuroinflammation underlies neuroprotective mechanism of several phytochemicals in neurodegenerative diseases. Oxid. Med. Cell. Longev. 2018:1972714. 10.1155/2018/1972714 30402203 PMC6196798

[B426] Wang L. Murphy N. P. Stengel A. Goebel-Stengel M. St Pierre D. H. Maidment N. T.. (2012). Ghrelin prevents levodopa-induced inhibition of gastric emptying and increases circulating levodopa in fasted rats. Neurogastroenterol. Motil. 24, e235–e245. 10.1111/j.1365-2982.2012.01904.x 22443313 PMC3345891

[B427] Wang S. S. Zhang Z. Zhu T. B. Chu S. F. He W. B. Chen N. H. (2018). Myelin injury in the central nervous system and alzheimer's disease. Brain Res. Bull. 140, 162–168. 10.1016/j.brainresbull.2018.05.003 29730417

[B428] Wang X. L. Su B. Lee H. G. Li X. Y. Perry G. Smith M. A.. (2009). Impaired balance of mitochondrial fission and fusion in alzheimer's disease. J. Neurosci. 29, 9090–9103. 10.1523/JNEUROSCI.1357-09.2009 19605646 PMC2735241

[B429] Wang X. L. Su B. Siedlak S. L. Moreira P. I. Fujioka H. Wang Y.. (2008). Amyloid-beta overproduction causes abnormal mitochondrial dynamics via differential modulation of mitochondrial fission/fusion proteins. Proc. Natl. Acad. Sci. U.S.A. 105, 19318–19323. 10.1073/pnas.0804871105 19050078 PMC2614759

[B430] Waseern T. Duxbury M. Ito H. Ashley S. W. Robinson M. K. (2008). Exogenous ghrelin modulates release of pro-inflammatory and anti-inflammatory cytokines in LPS-stimulated macrophages through distinct signaling pathways. Surgery 143, 334–342. 10.1016/j.surg.2007.09.039 18291254 PMC2278045

[B431] Werner H. LeRoith D. (2014). Insulin and insulin-like growth factor receptors in the brain: physiological and pathological aspects. Eur. Neuropsychopharmacol. 24, 1947–1953. 10.1016/j.euroneuro.2014.01.020 24529663

[B432] Wren A. M. Small C. J. Fribbens C. V. Neary N. M. Ward H. L. Seal L. J.. (2002). The hypothalamic mechanisms of the hypophysiotropic action of ghrelin. Neuroendocrinology 76, 316–324. 10.1159/000066629 12457042

[B433] Wu R. Q. Dong W. F. Cui X. X. Zhou M. Simms H. H. Ravikumar T. S.. (2007). Ghrelin down-regulates proinflammatory cytokines in sepsis through activation of the vagus nerve. Ann. Surg. 245, 480–486. 10.1097/01.sla.0000251614.42290.ed 17435556 PMC1877017

[B434] Wu R. Q. Dong W. F. Ji Y. X. Zhou M. Marini C. P. Ravikumar T. S.. (2008). Orexigenic hormone ghrelin attenuates local and remote organ injury after intestinal ischemia-reperfusion. PLoS ONE 3:e2026. 10.1371/journal.pone.0002026 18431503 PMC2295264

[B435] Wu R. Q. Dong W. F. Qiang X. L. Wang H. C. Blau S. A. Ravikumar T. S.. (2009a). Orexigenic hormone ghrelin ameliorates gut barrier dysfunction in sepsis in rats. Crit. Care Med. 37, 2421–2426. 10.1097/CCM.0b013e3181a557a2 19531942 PMC2742951

[B436] Wu R. Q. Zhou M. Dong W. F. Ji Y. X. Miksa M. Marini C. P.. (2009b). Ghrelin hyporesponsiveness contributes to age-related hyperinflammation in septic shock. Ann. Surg. 250, 126–133. 10.1097/SLA.0b013e3181ad85d6 19561473 PMC2797311

[B437] Xia S. J. Zhang X. Y. Zheng S. B. Khanabdali R. Kalionis B. Wu J. Z.. (2016). An update on inflamm-aging: mechanisms, prevention, and treatment. J. Immunol. Res. 2016:8426874. 10.1155/2016/8426874 27493973 PMC4963991

[B438] Xu H. Y. Li Y. Liu R. Q. Wu L. Zhang C. L. Ding N.. (2019). Protective effects of ghrelin on brain mitochondria after cardiac arrest and resuscitation. Neuropeptides 76:101936. 10.1016/j.npep.2019.05.007 31155149

[B439] Xu M. M. Liu L. Song C. F. Chen W. Gui S. Y. (2017). Ghrelin improves vascular autophagy in rats with vascular calcification. Life Sci. 179, 23–29. 10.1016/j.lfs.2016.11.025 27916732

[B440] Xu Y. H. Li Z. R. Yin Y. Lan H. Wang J. Zhao J.. (2015). Ghrelin inhibits the differentiation of T helper 17 cells through mTOR/STAT3 signaling pathway. PLoS ONE 10:e117081. 10.1371/journal.pone.0117081 25658305 PMC4319964

[B441] Xu Y. Q. Wei X. B. Liu X. Liao J. C. Lin J. P. Zhu C. S.. (2015). Low cerebral glucose metabolism: a potential predictor for the severity of vascular parkinsonism and parkinson's disease. Aging Dis. 6, 426–436. 10.14336/AD.2015.0204 26618044 PMC4657814

[B442] Yanagi S. Sato T. Kangawa K. Nakazato M. (2018). The homeostatic force of ghrelin. Cell Metab. 27, 786–804. 10.1016/j.cmet.2018.02.008 29576534

[B443] Yang H. H. Yang L. H. (2016). Targeting cAMP/PKA pathway for glycemic control and type 2 diabetes therapy. J. Mol. Endocrinol. 57, R93–R108. 10.1530/JME-15-0316 27194812

[B444] Yang S. Y. Lin S. L. Chen Y. M. Wu V. C. Yang W. S. Wu K. D. (2016). A low-salt diet increases the expression of renal sirtuin 1 through activation of the ghrelin receptor in rats. Sci. Rep. 6:32787. 10.1038/srep32787 27600292 PMC5013391

[B445] Yeung F. Hoberg J. E. Ramsey C. S. Keller M. D. Jones D. R. Frye R. A.. (2004). Modulation of NF-kappaB-dependent transcription and cell survival by the SIRT1 deacetylase. EMBO J. 23, 2369–2380. 10.1038/sj.emboj.7600244 15152190 PMC423286

[B446] Yin Y. Li Y. Zhang W. Z. (2014). The growth hormone secretagogue receptor: its intracellular signaling and regulation. Int. J. Mol. Sci. 15, 4837–4855. 10.3390/ijms15034837 24651458 PMC3975427

[B447] Yokochi M. (2007). Mesolimbic and mesocortical pathways in parkinson disease. Brain Nerve 59, 943–951. 10.11477/mf.1416100129 17886476

[B448] Yu J. H. Xu H. M. Shen X. L. Jiang H. (2016). Ghrelin protects MES23.5 cells against rotenone via inhibiting mitochondrial dysfunction and apoptosis. Neuropeptides 56, 69–74. 10.1016/j.npep.2015.09.011 26459609

[B449] Zhang J. Ke K. F. Liu Z. Qiu Y. H. Peng Y. P. (2013). Th17 cell-mediated neuroinflammation is involved in neurodegeneration of a beta(1-42)-induced alzheimer's disease model rats. PLoS ONE 8:e75786. 10.1371/journal.pone.0075786 24124514 PMC3790825

[B450] Zhang J. B. Ng S. K. Wang J. G. Zhou J. Tan S. H. Yang N. D.. (2015). Histone deacetylase inhibitors induce autophagy through FOXO1-dependent pathways. Autophagy 11, 629–642. 10.1080/15548627.2015.1023981 25919885 PMC4502718

[B451] Zhang R. L. (2017). Ghrelin suppresses inflammation in HUVECs by inhibiting ubiquitin-mediated uncoupling protein 2 degradation. Int. J. Mol. Med. 39, 1421–1427. 10.3892/ijmm.2017.2977 28487946 PMC5428956

[B452] Zhang X. Zhang G. Zhang H. Karin M. Bai H. Cai D. (2008). Hypothalamic IKKbeta/NF-kappaB and ER stress link overnutrition to energy imbalance and obesity. Cell 135, 61–73. 10.1016/j.cell.2008.07.043 18854155 PMC2586330

[B453] Zhang Y. Huang N. Q. Yan F. Jin H. Zhou S. Y. Shi J. S.. (2018). Diabetes mellitus and alzheimer's disease: GSK-3 beta as a potential link. Behav. Brain Res. 339, 57–65. 10.1016/j.bbr.2017.11.015 29158110

[B454] Zhao C. Ling Z. Newman M. B. Bhatia A. Carvey P. M. (2007). TNF-alpha knockout and minocycline treatment attenuates blood-brain barrier leakage in MPTP-treated mice. Neurobiol. Dis. 26, 36–46. 10.1016/j.nbd.2006.11.012 17234424 PMC1892817

[B455] Zhao W. Wang J. Varghese M. Ho L. Mazzola P. Haroutunian V.. (2015). Impaired mitochondrial energy metabolism as a novel risk factor for selective onset and progression of dementia in oldest-old subjects. Neuropsychiatr. Dis. Treat. 11, 565–574. 10.2147/NDT.S74898 25784811 PMC4356684

[B456] Zhao W. Q. De Felice F. G. Fernandez S. Chen H. Lambert M. P. Quon M. J.. (2008). Amyloid beta oligomers induce impairment of neuronal insulin receptors. Faseb J. 22, 246–260. 10.1096/fj.06-7703com 17720802

[B457] Zhao Z. Liu H. Xiao K. Yu M. Cui L. Zhu Q.. (2014). Ghrelin administration enhances neurogenesis but impairs spatial learning and memory in adult mice. Neuroscience 257, 175–185. 10.1016/j.neuroscience.2013.10.063 24211302

[B458] Zhou M. Yang W. L. Aziz M. Ma G. F. Wang P. (2017). Therapeutic effect of human ghrelin and growth hormone: attenuation of immunosuppression in septic aged rats. Biochim. Biophys. Acta Mol. Basis Dis. 1863, 2584–2593. 10.1016/j.bbadis.2017.01.014 28115288 PMC5519455

[B459] Zigman J. M. Bouret S. G. Andrews Z. B. (2016). Obesity impairs the action of the neuroendocrine ghrelin system. Trends Endocrinol. Metab. 27, 54–63. 10.1016/j.tem.2015.09.010 26542050 PMC4814209

[B460] Zilberter Y. Zilberter M. (2017). The vicious circle of hypometabolism in neurodegenerative diseases: ways and mechanisms of metabolic correction. J. Neurosci. Res. 95, 2217–2235. 10.1002/jnr.24064 28463438

